# Artificial intelligence for fracture detection on computed tomography: a comprehensive systematic review and meta-analysis of diagnostic test accuracy in non-commercial and commercial solutions

**DOI:** 10.1007/s10140-026-02437-7

**Published:** 2026-02-07

**Authors:** Julius Husarek, Anika M. C. Fuchss, Thomas D. Ruder, Stavroula Mougiakakou, Aristomenis Exadaktylos, Katharina Wahedi, Martin Müller

**Affiliations:** 1 Department of Emergency Medicine, Inselspital, Bern University Hospital, University of Bern, Rosenbühlgasse 27, 3010 Bern, Switzerland; 2 University of Bern, Bern, Switzerland; 3 Department of Diagnostic, Interventional and Pediatric Radiology, Inselspital, Bern University Hospital, University of Bern, Bern, Switzerland; 4 ARTORG Center for Biomedical Engineering Research, University of Bern, Bern, Switzerland

**Keywords:** Artificial intelligence, Computer-aided diagnosis, Computed tomography, Diagnostic test accuracy, Systematic review and meta-analysis

## Abstract

**Abstract:**

Rising patient volumes, the increasing use of computed tomography (CT) imaging in emergency departments and the resulting prolonged waiting times highlight the urgent need for efficient and accurate diagnostic tools, especially given that the number of experienced healthcare professionals is not increasing at the same pace. Artificial intelligence (AI) has emerged as a promising tool to support fracture detection on CT scans, with the potential to streamline diagnostic workflows in emergency care. However, concerns exist regarding dataset bias, limited external testing, and methodological variability. This systematic review and diagnostic test accuracy (DTA) meta-analysis aimed to comprehensively assess the diagnostic accuracy of AI-driven fracture detection solutions, with a particular focus on the effect of the testing strategy, cohort composition and commercial availability on diagnostic accuracy. The Cochrane Handbook for Systematic Reviews of DTA and reported according to PRISMA-DTA guidelines were followed. We systematically searched Embase, MEDLINE, Cochrane Library, Web of Science, and Google Scholar for studies published from January 2010 onward, complemented by citation chasing and manual searches for commercial AI fracture detection solutions (CAAI-FDS). Two reviewers independently conducted study selection, data extraction, and risk of bias assessment using a modified QUADAS-2 tool. Statistical analysis was conducted using STATA 18.1 and the –metadta– command. Primary analyses evaluated diagnostic accuracy (sensitivity and specificity) of stand-alone AI based on (1) cohort type (selected vs. unselected), (2) test dataset origin (internal vs. external), and (3) level of analysis (patient-wise, vertebra-wise, rib-wise). Secondary analyses explored accuracy differences according to (1) CAAI-FDS, (2) anatomical region and (3) reader type (stand-alone AI, human unaided, human aided by AI). Forest plots visualized results, and heterogeneity was measured using generalized I^2^ statistics. Out of 7683 identified articles, 44 studies were included for meta-analysis. 14 CAAI-FDS were identified. Primary analyses of stand-alone AI showed moderate sensitivity (0.85, 95% CI: 0.77, 0.90) and good specificity (0.92, 95% CI: 0.87, 0.95) in unselected patient cohorts, whereas selected cohorts achieved slightly higher sensitivity (0.89, 95% CI: 0.80, 0.94). Diagnostic accuracy was higher when studies used internal test datasets (sensitivity 0.94, 95% CI: 0.88, 0.97; specificity 0.91, 95% CI: 0.86, 0.94) compared to external test datasets (sensitivity 0.85, 95% CI: 0,77, 0.91; specificity 0.92, 95% CI: 0.89, 0.95). Vertebra- and rib-wise analyses achieved higher specificity (0.98) compared to patient-wise analysis (0.92, 95% CI: 0.89, 0.95), although sensitivity remained moderate across all levels (0.85–0.89). Secondary analyses showed variability among CAAI-FDS (sensitivities 0.68–0.80; specificities 0.87–0.97) and by anatomical region, with the highest sensitivity for skull (0.90, 95% CI: 0.85, 0.93), rib (0.92, 95% CI: 0.83, 0.96) and pelvis fractures (1.00), and lowest for spine fractures (0.82, 95% CI: 0.73, 0.88). Stand-alone AI showed moderate to good diagnostic accuracy, slightly outperforming unaided human readers, with minimal further improvement when humans were aided by AI. While AI demonstrates promising diagnostic accuracy in fracture detection, study biases, stringent patient selection, and lack of external testing raise concerns about real-world applicability. Commercially available solutions tend to underperform compared to pooled study results, highlighting the gap between research settings and clinical practice. Future efforts should focus on reducing bias, improving generalizability and robustness, as well as conducting prospective trials to assess AI’s true impact on clinical outcomes.

**Graphical abstract:**

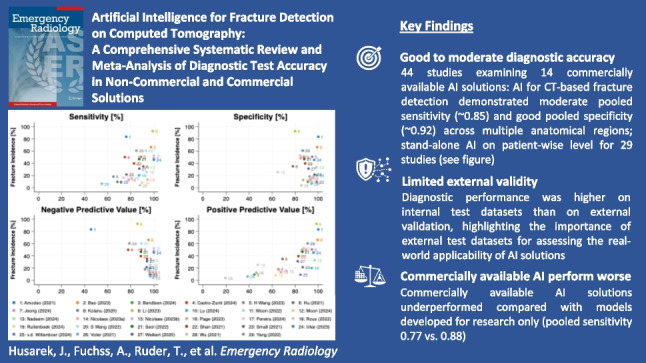

**Supplementary Information:**

The online version contains supplementary material available at 10.1007/s10140-026-02437-7.

## Introduction

Emergency departments are the front lines of hospital care, where unpredictable patient influx, overcrowding, and high-stakes decision-making create a demanding and time-sensitive environment. Ensuring rapid, accurate diagnoses while maintaining efficiency and patient safety is a constant challenge. In recent years, artificial intelligence (AI) has emerged as a promising tool to optimize workflows, reduce clinician burden, and enhance diagnostic accuracy — particularly in imaging-based assessments such as fracture detection on computed tomography (CT) scans. However, high sensitivity and specificity (and even high ROC-AUC) can still coincide with low precision, leading to substantial false-positive burden and limited clinical usability.

The use of CT imaging has grown at a significantly higher rate than the number of patients [1, 2]. Several factors contribute to this trend, including lower cost, increased machine availability, and a stronger emphasis on diagnostic certainty and liability protection. However, this rise in CT utilization comes at a cost: the increased demand for interpretation, screening, and reporting has substantially increased the workload for radiologists [3]. Additionally, radiological imaging has been identified as a major contributor to prolonged emergency department stays, particularly for ambulatory and surgical patients [4–6], further exacerbating the issue of overcrowding and accounts for a fifth of emergency resources used [7].

In 2019, the age-standardized incidence rate of fractures was more than 2296 cases per 100,000 people [8]. Roughly a fifth of emergency room visits are due to injuries and falls and require imaging for further diagnostics [9]. Although global age-standardized rates of fracture incidence, prevalence, and years lived with disability declined slightly between 1990 and 2019, the overall number of cases rose significantly [10]. As the global population continues to age, the impact of e.g. hip fractures is expected to grow, with their incidence projected to double by 2050 [11]. The estimated number of emergency department visits for fractures related to falls grew significantly, increasing from 574,000 in 2001 to 984,000 in 2020 [12].

AI is revolutionizing workflows across various fields, including healthcare and medical imaging [13]. The rapid development of AI, both in medicine and beyond, has been driven by advancements in computational power and the increasing availability of large-scale data. In particular, the widespread implementation of electronic health records and digital medical imaging archives has provided extensive datasets for training AI models [13]. The use of AI in radiology is multifaceted, with various proposed applications aimed at enhancing efficiency and accuracy. Among its many potential benefits, AI is expected to accelerate triage, reduce human error, and optimize workflows.

Early studies and reviews have found that AI is often non-inferior to radiologists for fracture detection [14–17] and better than non-expert human readers [17]. Good diagnostic accuracy has been shown in reviews examining e.g. vertebral fractures [18] and hip fractures [19, 20]. Several AI-based algorithms have received market approval and are commercially available and widely implemented. However concerns have been raised concerning the methodological quality of diagnostic test accuracy (DTA) of these AI solutions [21]. For instance, the review by Jung et al. found that only 20% of studies were tested on external data sets [22]. Dataset biases may arise from patient selection or strict inclusion criteria, which exclude equivocal or difficult images. Many AI algorithms are trained and tested on limited or homogeneous datasets, which may lead to overly optimistic performance and may not generalize to broader or more diverse patient population. Consequently, AI models may perform well on typical cases but struggle with rare fracture patterns, pediatric patients, or images from different hospitals. Furthermore, as many studies apply split-sampling or resampling techniques no external validation is applied [21]. Finally, variability in study designs – some evaluating only AI, others comparing AI with radiologists, others with both AI and radiologists – limits the comparability of results across studies.

To date, no review has systematically assessed how adherence to methodological standards in studies evaluating AI-based algorithms for fracture detection in CT scans influences the reported diagnostic accuracy. Moreover, it remains unclear to what extent diagnostic performance varies depending on anatomical region, commercial availability of AI tools, and the level of analysis. This review is the first to comprehensively address these gaps. The purposes of this DTA systematic review with meta-analyses were therefore:

1. **Comprehensive review:** summarize peer-reviewed studies assessing the diagnostic accuracy of AI-FD from CT images and provide an overview of commercially available AI fracture detection solutions (CAAI-FDS).
2. **Primary analyses:** evaluate the diagnostic accuracy of AI-FD with a focus on the impact of
  3. cohort selection (e.g., exclusion of patient cases with implants or specific age groups),
  4. testing with external and internal test datasets,
  5. analysis at different levels (i.e., patient-wise, rib-wise or vertebra-wise).
3. **Secondary analyses**: investigate further factors influencing diagnostic accuracy
  7. the choice of CAAI-FDS ,
  8. the anatomical region studied,
  9. reader type (i.e., stand-alone AI, human unaided, and human aided by AI).

## Methods

### Protocol and registration

This study was conducted following the Cochrane Handbook for Systematic Reviews of DTA [23] and is reported according to the Preferred Reporting Items for Systematic Reviews and Meta-Analyses of DTA (PRISMA-DTA) [24]. The protocol is registered in PROSPERO under ID CRD42024509803.

### Eligibility criteria

Study eligibility was defined with the PICOS scheme:

- *Population:* patients, irrespective of demographic characteristics, who have undergone CT scans for fracture detection.
- *Intervention:* studies utilizing AI-FD algorithms or models designed to diagnose bone fractures in CT images.
- *Comparator:* reference standard used to confirm the presence or absence of fractures, as defined in each study. This commonly included written CT reports or evaluations by radiologists, either through independent review or consensus reading.
- *Outcomes:* diagnostic accuracy metrics such as sensitivity and specificity, along with true positives (TP), false positives (FP), true negatives (TN), and false negatives (FN).
- *Study Design:* original DTA research articles published in peer-reviewed journals in English from January 2010 onward. Exclusion of letters to the editor, systematic reviews, conference abstracts, case reports, case series, opinion pieces and editorials.

Studies that did not fulfill the PICOS criteria were excluded from the review.

### Information sources

With the aim to achieve the highest possible coverage and inclusiveness, the following databases were systematically searched to identify relevant publications, as recommended by Bramer et al. [25]: Embase (via Ovid), MEDLINE (via Ovid), Cochrane Library, Web of Science, and the first 200 articles from Google Scholar. Additionally, IEEE Xplore (via Google Scholar) was included to enhance coverage. The reference lists of relevant articles were screened using the CitationChaser tool [26] to efficiently identify further studies through forward and backward citation chasing. If further data or clarification was required, the study authors were contacted directly.

### Search strategy

Following Bramer et al. [27]s recommendation for systemic construction of the search strategy, the review topic was initially divided into specific search blocks: “Artificial Intelligence”, “Computed Tomography”, and “Fracture”. Additional keywords and synonyms were identified through reference lists on similar topics. Boolean operators (AND, OR), proximity operators (e.g., NEXT), and truncation or wildcard symbols were used to enhance the search. Medical Subject Headings (e.g., Emtree, MeSH) were incorporated where applicable, with adaptations to the syntax requirements of the individual databases. An initial search was conducted in Embase via the Ovid platform, and the strategy was adapted for use across the other databases. The final search was completed on June 2nd, 2024. The citation chasing was carried out on January 2, 2025. The full list of different database-specific search syntaxes is available in Supplement 1.

### Study selection

Search results from all databases were collected and deduplicated in EndNote 20.5 (Clarivate Analytics, PA, USA), followed by a manual check for accuracy [28]. The remaining articles were then imported into the web-based platform Rayyan [29]. Two independent reviewers (JH, AF) conducted title, abstract and full-text screening in successive stages. Any discrepancies were discussed and resolved by consensus. If disagreements persisted, a senior researcher (MM) was consulted for the final decision.

### Commercially available AI fracture detection solutions identification

Two independent online resources were used to classify identified solutions as CAAI-FDS: The Health AI Register [30] and the U.S. Food and Drug Administration (FDA’s) AI/ML-Enabled Medical Device List [31]. The Health AI Register provides information on AI-based solutions in clinical radiology with a focus on Conformité Européenne (CE) marked devices. The FDA list identifies medical devices that have met the agency’s requirements for market approval, specifically for the US market. The Health AI Register was searched on 23 December 2024, while the FDA list was reviewed on 29 December 2024. All identified products and companies were reviewed to verify its current regulatory status and assess its clinical applicability. Additionally, manual searches were conducted for AI products and solutions, and all products mentioned in the included studies were examined.

### Data extraction

Data extraction was conducted by one reviewer (AF) using Microsoft Excel 16.6 (Microsoft Corporation, WA, USA) and independently cross-checked by a second reviewer (JH). Any discrepancies were discussed and resolved by mutual agreement. Persisting disagreements were consulted with and decided by a third researcher (MM). For studies with unclear or missing data, the corresponding author was contacted to request clarification or additional information. The form for data extraction was piloted on a subset of studies to ensure clarity and consistency.

Regarding the cohort composition, studies were categorized as ‘selected’ if specific patients, e.g. with implants, pathological fractures or metastases were excluded. In contrast, ‘unselected’ cohort was defined as close to clinical real-life settings without selecting the patient or exclusion of difficult-to-diagnose cases.

The origin of test datasets was defined by their relation to the study team. The ‘external’ test datasets were collected independently of the investigators, whereas ‘internal’ test datasets were generated by the study group or from the same hospital that developed the AI algorithm. All studies evaluating CAAI-FDS were classified as using external test datasets, as the development of these algorithms was conducted by the specific companies.

For reader types, definitions were based on the degree of AI involvement. ‘Stand-Alone AI’ referred to AI algorithms performing diagnostic tasks independently without human input, ‘Human Unaided’ denoted clinicians interpreting CT scans without any assistance from AI, and ‘Human Aided’ described scenarios in which clinicians were supported by AI output during their assessment.

The level of analysis was classified into two main diagnostic assessments: i) ‘patient-wise’ and ii) ‘region-wise’. Patient-wise referred to diagnostic accuracy at the level of each patient, while region-wise assessments are a heterogeneous group including different subgroups such as ‘vertebra-wise’, ‘rib-wise’, or ‘sample-wise’ (studies evaluating distinct fracture sites or annotated image regions rather than whole-patient assessments).

If multiple levels of analysis, cohort compositions or test datasets were reported within a study, data were extracted and analyzed separately. If several AI algorithms were reported in a single publication, the performance metrics of the model with the highest diagnostic accuracy as judged by the author were extracted. For ‘vertebra-wise’ and ‘rib-wise’, contingency tables were calculated based on the given data and if necessary, extrapolation using specific ranges (e.g. L1-L5 or ribs 2–12). In addition, in line with the Genant classification [32] of osteoporotic vertebral fractures only grades 2/3 were extracted, since grade 1 is considered too similar to unfractured vertebrae.

A detailed list of all extracted variables, derived from the study codebook, is provided in Supplement 2.

### Risk of Bias and applicability

The Quality Assessment of Diagnostic Accuracy Studies 2 (QUADAS-2) [33] was used to assess the risk of bias (RoB) and applicability of the included studies, following Cochrane Collaboration recommendations. The original QUADAS-2 signaling questions were modified to align with the objectives of this review. A full list of the signaling questions is provided in Supplement 3. QUADAS-2 assesses four key domains for RoB: cohort selection, index test, reference standard, and funding. Each domain was classified as having a ‘low’, ‘high’ or ‘unclear’ risk of bias. Domain-level QUADAS-2 judgments constituted the primary risk-of-bias assessment.

In addition, to facilitate concise descriptive synthesis across a large and heterogeneous body of evidence, we applied an a priori, review-specific, non-standard summary of overall RoB based on the number of domains rated as ‘high’. Studies were categorized as having a low RoB (no ‘high’ ratings), moderate RoB (1–2 ‘high’ ratings across different domains), or high RoB (more than 2 ‘high’ ratings across different domains). This summary was used for descriptive purposes only and does not replace the domain-level QUADAS-2 assessment.

For applicability, the original QUADAS-2 domains were adapted to evaluate three areas: test dataset, commercial availability, reference standard. As with study selection and data extraction, two reviewers (JH, AF) independently conducted the assessments, with disagreements resolved through discussion or by a third reviewer (MM).

### Data synthesis and statistical analysis

This systematic review uses statistical methodology consistent with the Cochrane Handbook for Systematic Reviews of Diagnostic Test Accuracy [23]. All analyses were conducted in STATA 18.1 (StataCorp, College Station, TX, USA) with the use of the – *metadta* – command, which facilitates meta-analysis and meta-regression of DTA data. This approach optimizes complex statistical modeling by incorporating a comprehensive set of validated procedures designed specifically for diagnostic accuracy studies. To allow for the inherent correlation between sensitivity and specificity, a generalized linear mixed model with a logit link function was used as recommended in Chapter 10 of the Cochrane Handbook for DTA [34].

Various stratified analyses were conducted, including categorization by test dataset origin, cohort composition, level of analysis, CAAI-FDS, anatomical region, rater types, reference standard, and industry funding status, with a descriptive evaluation of pooled estimates, without statistical comparison. The primary diagnostic accuracy measures reported in this systematic review were sensitivity and specificity, both reported with their corresponding 95% confidence interval (CI). In addition, between-study heterogeneity was assessed using generalized I^2^ as proposed by Zhou & Dendukuri [35].

To better characterize included patient populations and to provide a more comprehensive assessment of diagnostic performance, disease prevalence, positive predictive value (PPV), negative predictive value (NPV), and F1 score were additionally reported for each study contributing to the sensitivity–specificity forest plots in a supplementary table.

When methodologically appropriate and feasible, multiple available contingency tables from a single included study were combined by summing the individual cells (TP, FN, FP, TN) to create a more comprehensive contingency table for that study.

The F1 score was included as a complementary performance metric and is defined as the harmonic mean of positive predictive value (precision) and sensitivity (recall):

$$\textrm{F}1=2\times \frac{\textrm{PPV}\times \textrm{Sensitivity}}{\textrm{PPV}+\textrm{Sensitivity}}$$

By assigning equal weight to precision and sensitivity, the F1 score penalizes large imbalances between false-positive burden and case detection performance. This metric may facilitate comparison across studies, particularly in contexts characterized by class imbalance or low disease prevalence.

To facilitate interpretation, sensitivity and specificity were grouped into five categories: *Very poor:* <0.60; *Poor:* 0.60 to <0.70; *Moderate*: 0.70 to <0.90; *Good:* 0.90 to <0.95 and *Excellent*: ≥0.95. Forest plots were used to illustrate the different analyses along with their corresponding diagnostic accuracy measures.

### Code availability

The STATA 18.1 code (StataCorp, College Station, TX, USA) used for the statistical analyses is provided as a supplementary document.

## Results

### Study selection

The systematic search identified a total of 6015 articles. After removing of duplicates, 4074 articles remained for title and abstract screening. Of these, 125 articles underwent full-text screening, and 38 DTA studies met the inclusion criteria and were included in the initial selection.

**Fig. 1:**
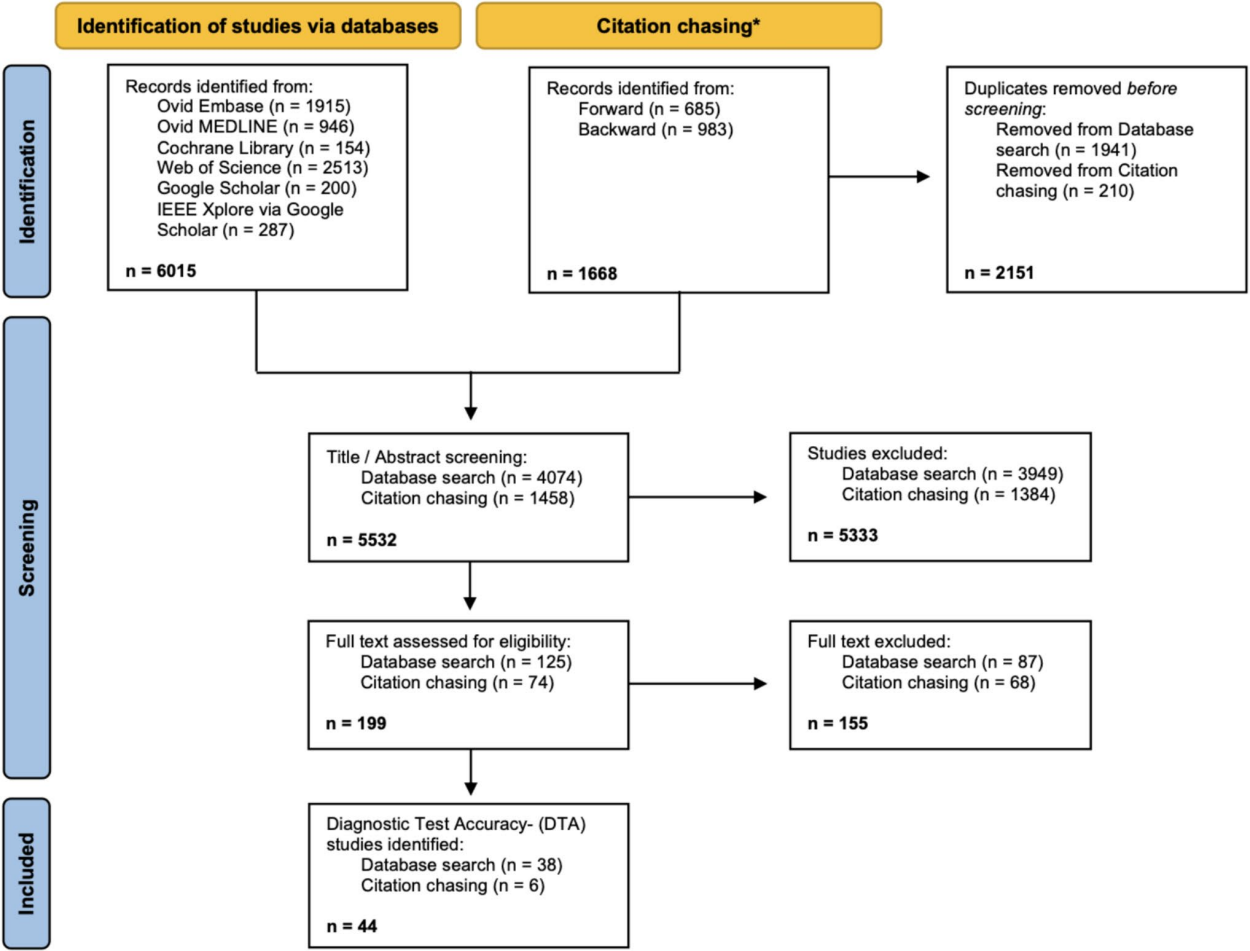
PRISMA Flowchart

Following forward and backward citation chasing of these 38 studies, 1668 additional articles were identified. After removal of duplicates, 1458 unique articles remained for further screening. Ultimately, 6 additional DTA studies met the inclusion criteria, bringing the total number of included studies to 44 (see PRISMA flow chart, Fig. 1). Across all included studies, a total of 246,951 patients were evaluated, including 38,854 fracture-positive analysis units. The descriptive characteristics of all included studies are presented in Table 1.

**Table 1:** Descriptive Characteristics of Included Studies (Sorted by Author)

Author	Main Target	Patients (n)	Females (%)	Males (%)	Mean Age ± SD (Median)	Total Units Analyzed (n)	Fracture Positive Units (n)	Analysis Level	Test Dataset	Cohort Selection
Al-Helo et al. (2012) [54]	Spine (VCF)	50	N/A	N/A	N/A	250	23	Vertebra-Wise	ITD	N/S
Amodeo et al. (2021) [55]	CMF	208	N/A	N/A	N/A	30	25	Patient-Wise	ITD	N/S
Bao et al. (2023) [41]	Orbita	497	46.5	53.5	40 ± 16	302	200	Sample-Wise	ITD	N/S
								Patient-Wise		
Bendtsen et al. (2024) [37]	Spine (VCF)	10,012	N/A	N/A	N/A	1000	95	Patient-Wise	ETD	Selected
Burns et al. (2017) [46]	Spine (VCF)	150	61.3	38.7	73 ± 11	150	75	Patient-Wise	ETD	Selected
Castro-Zunti et al. (2024) [56]	Rib	2199	38.6	61.4	60 ± 17	2000	1000	Patient-Wise	ITD	Selected
Choi et al. (2023) [38]	Spine (VCF)	1042	N/A	N/A	N/A	227	110	Patient-Wise	ITD	Unselected
						1135	122	Vertebra-Wise		
Erne et al. (2021) [57]	Acetabulum	159	N/A	N/A	N/A	64	32	Sample-Wise	ITD	Selected
Hu et al. (2021) [58]	Rib	1697	N/A	N/A	N/A	252	88	Patient-Wise	ITD	Unselected
Jeong et al. (2024) [59]	Nose	740	N/A	N/A	N/A	50	24	Patient-Wise	ITD	Selected
Kolanu et al. (2020) [60]	Spine (VCF)	1696	N/A	N/A	N/A	1570	282	Patient-Wise	ETD	Unselected
Lee et al. (2024) [61]	Spine (VCF)	589	N/A	N/A	N/A	981	99	Vertebra-Wise	ITD	Selected
Li et al. (2023) [50]	Rib	18,172	41.8	58.2	58 ± 14	1612	955	Patient-Wise	ITD	Selected
						2319^$^	2145^$^		ETD	
						55,250^$^	10,292^$^	Rib-Wise		
Liu et al. (2021) [62]	Rib	393	37.7	62.3	46 ± 14	1179^§$^	561^§$^	Patient-Wise	ETD	Selected
						28,296^§$^	1641^§$^	Rib-Wise		
Lu et al. (2024) [63]	Skull	3500	22.6	77.4	55 ± 21	671^§$^	170^§$^	Patient-Wise	ITD	Unselected
						4026*^§$^	1020*^§$^			
Moon et al. (2022) [64]	Nose	780	N/A	N/A	N/A	40	21	Patient-Wise	ITD	N/S
Moon et al. (2024) [65]	CMF	1134	N/A	N/A	N/A	232	116	Patient-Wise	ITD	N/S
Nadeem et al. (2024) [66]	Spine (VCF)	3231	47.8	52.2	59 ± 9	3231	1062	Patient-Wise	Not specified	Selected
						40,050	2259	Vertebra-Wise		
Nicolaes et al. (2023a) [67]	Spine (VCF)	4810	55.2	44.8	N/A (62)	48,584	899	Vertebra-Wise	ETD	Unselected
						4810	623	Patient-Wise		
Nicolaes et al. (2023b) [39]	Spine (VCF)	2609	49.1	50.9	N/A (68)	1943	297	Patient-Wise	ETD	Selected
						24,930	663	Vertebra-Wise		
Page et al. (2023) [45]	Spine (VCF)	1087	54	46	N/A (73)	1087	137	Patient-Wise	ETD	Unselected
Pereira et al. (2024) [68]	Spine (VCF)	899	N/A	N/A	70	899	145	Patient-Wise	ETD	Selected
Polzer et al. (2024) [69]	Spine (Acute)	257	54.5	45.5	68 ± 14	448	96	Vertebra-Wise	ITD	Selected
Potter et al. (2024) [47]	Spine (VCF)	325	51.1	48.9	59 ± 17	480	82	Vertebra-Wise	ITD	Selected
						20	10	Patient-Wise		
Roux et al. (2022) [40]	Spine (VCF)	143,274	47.8	52.2	73 ± 9	500	127	Patient-Wise	ETD	Unselected
Ruitenbeek et al. (2024) [53]	Spine (Cervical)	2973	37.5	62.5	55 ± 20	2036	167	Patient-Wise	ETD	Unselected
Seol et al. (2022) [70]	Nose	2535	46.5	53.5	46 ± 20	507	237	Patient-Wise	ITD	N/S
Shan et al. (2021) [71]	Skull	4782	46	54	N/A (54)	235	93	Patient-Wise	ETD	N/S
Small et al. (2021) [72]	Spine (Cervical)	695	45.5	54.5	N/A (61)	665^$^	143^$^	Patient-Wise	ETD	Unselected
Tian et al. (2024) [73]	Spine (Lumbar)	396	55.6	44.4	N/A (70)	887	131	Vertebra-Wise	ETD	Selected
						152	32		ITD	
Tomita et al. (2018) [48]	Spine (VCF)	129	N/A	N/A	N/A	129^$^	81^$^	Patient-Wise	ITD	Unselected
Tong et al. (2023) [74]	Zygoma	379	30.1	69.9	35 ± 13	95	44	Sample-Wise	ITD	Selected
Ukai et al. (2023) [75]	Pelvis	205	43.4	56.6	63 ± 19	205	93	Patient-Wise	ITD	Selected
Van den Wittenboer et al. (2024) [76]	Spine (Cervical)	2368	39.2	60.8	N/A (48)	2368^$^	221^$^	Patient-Wise	ETD	Selected
Voter et al. (2021) [77]	Spine (Cervical)	1904	49.7	50.3	60 ± 22	1904	122	Patient-Wise	ETD	Unselected
Weikert et al. (2020) [78]	Rib	511	N/A	N/A	58 ± 23	510	159	Patient-Wise	ETD	Unselected
Wu et al. (2021) [79]	Rib	10,943	41.4	58.6	55 ± 17	8051	313	Patient-Wise	ITD	Selected
H Wang et al. (2023) [80]	CMF	2435	N/A	N/A	N/A	192	97	Patient-Wise	ETD	Selected
S Wang et al. (2022) [49]	Rib	13,821	39.3	60.7	51 ± 15	1628	420	Patient-Wise	ITD	Selected
						1613^$^	976^$^		ETD	
						39,072	1665	Rib-Wise	ITD	
						38,712^$^	3412^$^		ETD	
X Wang et al. (2022) [81]	Mandible	686	27.6	72.4	36 ± 13	3672*	886*	Sample-Wise	ITD	Selected
Y Wang et al. (2024) [82]	Spine (VCF)	1097	76.6	23.4	73 ± 8	17,658	2536	Vertebra-Wise	ITD	Selected
						2823	398		ETD	
Yang et al. (2022) [42]	Nose	252	59.1	40.9	46 ± 13	76^§$^	46^§$^	Patient-Wise	ITD	Unselected
Zhang et al. (2023) [83]	Spine (Acute)	1217	62.5	37.5	62 ± 17	11,356	374	Vertebra-Wise	ITD	Selected
Zhou et al. (2023) [43]	Rib	113	24.8	75.2	50 ± 15	2712^§$^	712^§$^	Rib-Wise	ETD	Unselected

*SD* Standard Deviation; *VCF* Vertebral Compression Fracture; *ITD* Internal Test Dataset; *ETD*: External Test Dataset; *CMF* Craniomaxillofacial; *§* Human aided; *$* Human unaided; *: Pooled; N/S: Not Specified; N/A: Not Available

### Commercially available AI fracture detection solutions identification

A total of 14 CAAI-FDS from twelve different companies were identified for use on CT images. These solutions are primarily from China (*n* = 5) and Israel (*n* = 3), followed by France (*n* = 2) with one solution each from Austria, India, Australia, Poland, and Taiwan.

Many solutions are specialized on fracture detection of a specific anatomical region, including rib fractures (Aidoc RibFx, uAI EasyTriage-Rib, CT AI-assisted Rib Fracture), vertebral compression fractures (IB Lab FLAMINGO, HealthOST Bone Solution, CINA-VCF), and cervical spine fractures (Aidoc C-Spine, CINA-CSpine). Some also extend their functionality beyond fractures, such as InferRead CT Bone, which detects bone metastases and tumors. Nine solutions, RibFx, qER, Annalise Enterprise CTB, C-Spine, HealthOST Bone Solution, CINA-CSpine, CINA-VCF, DeepCT, and uAI EasyTriage-Rib, have received FDA 510(k) clearance as Class II devices (indicating the AI solution is considered safe and effective for clinical use and having demonstrated substantial equivalence to an already approved device). Ten solutions were CE marked. Five received both CE marking and FDA clearance. These are RibFx, C-Spine, Annalise Enterprise CTB, DeepCT and uAI EasyTriage-Rib.

Most AI systems are designed for use in adult populations (*n* = 7). Only one solution is aimed at all age groups (SenseCare Lung CT). Two solutions are specifically targeted for patients over the age of 50 years (IB Lab FLAMINGO and CINA-VCF). All identified CAAI-FDS are designed to support human interpretation or triage, but not for autonomous use. The descriptive characteristics of all included CAAI-FDS are presented in Table 2.

**Table 2:** Descriptive Characteristics of Included Commercial Fracture Detection Solutions for Computed Tomography (Sorted by Company Name)

Product Name	Company	Country	Speciality	CE	FDA	Target Condition	Market Entry	Population
Annalise Enterprise CTB	annalise.ai	Australia	Neuro	Class IIb, MDR	Class II*	Neurological findings, including cranial fractures	2022	Adults
BrainScan CT	BrainScan Inc.	Poland	Neuro	Class IIa, MDR	/	Neurological findings, including cranial fractures	2020	Adults
C-Spine (CSF)	Aidoc	Israel	MSK	Class I, MDD	Class II	Cervical spine fractures	2021	N/A
CINA-CSpine	Avicenna.AI	France	MSK	/	Class II	Cervical spine fractures	2024	N/A
CINA-VCF	Avicenna.AI	France	MSK	/	Class II	VCF	2024	Adults >50
CT AI-assisted Rib Fracture	Huiying Medical Technology	China	MSK	/	/	Rib fractures	N/A	N/A
DeepCT	Deep01 Limited	Taiwan	Neuro	Class I, MDD	Class II	Neurological findings, including cranial fractures	2019	Adults
Health VCF^$^	Zebra Medical Vision^$^	Israel	MSK	/	Class II	Mild to moderate VCF	2020	N/A
HealthOST Bone solution	Nanox.AI^§^	Israel	MSK	/	Class II	VCF	2020	N/A
IB Lab FLAMINGO	ImageBiopsy Lab	Austria	MSK	Class IIa, MDR	/	VCF, Osteoporosis	2023	Adults >50
InferRead CT Bone	Infervision	China	MSK	Class IIb, MDR	/	Bone fractures, tumors, metastases	2024	Adults
qER	Qure.AI	India	Neuro	Class IIb, MDR	Class II^&^	Neurological findings, including cranial fractures	2018	Adults
Rib fractures (RibFx)	Aidoc	Israel	MSK	Class I, MDD	Class II	Rib fractures	2021	N/A
SenseCare Lung CT	SenseTime	China	Chest	Class IIb, MDR	/	Pulmonary nodules, pneumonia lesions and fractures	2020	All Ages
uAI EasyTriage-Rib	United Imaging Intelligence	China	MSK	Class IIa, MDD	Class II	Rib fractures (only if 3 or more)	2021	N/A

*CE*: Conformité Européenne; *FDA* U.S. Food and Drug Administration; *MDR* Medical Device Regulations; *MDD* Medical Devices Directive; *MSK* Musculoskeletal; *VCF*: Vertebral Compression

*FDA 510(k) clearance as CADt for seven acute findings: acute subdural/epidural hematoma, acute subarachnoid hemorrhage, Intra-axial hemorrhage, intraventricular hemorrhage, obstructive hydrocephalus, vasogenic edema, and mass effect

^$^Zebra Medical Vision was acquired by Nanox.AI [ 84 ]. The product HealthVCF is not on the market anymore

^§^Previously part of Zebra Medical Vision

^&^In the U.S., FDA 510(k) clearance only for quantifying the volume of intracranial structures and lesions

### Risk of Bias and applicability

Of the 44 included studies, the majority (*n* = 36) were assessed as having a moderate risk of bias (RoB). Four studies [36–39] were rated as having high RoB, while only four studies [40–43] were considered low risk of bias across all domains. Inadequate reporting of patient characteristics or selective cohort inclusion was the most frequently observed concern, affecting 32 of the 44 studies. An inadequate reference standard was identified in 25 studies. Six studies reported industry-funding [36–39, 44, 45].

With respect to applicability, only a quarter (*n* = 11) of all studies evaluated AI products which were commercially available for clinical use. Half of the studies (*n* = 22) lacked external validation. All studies were deemed to have low applicability concerns for the reference standard. Risk of bias and applicability assessments for each study are summarized in Table 3 and provided as a spreadsheet in Table 4. A detailed table with all included studies and corresponding assessment of the signaling question is available in Supplement 3.

**Table 3:**
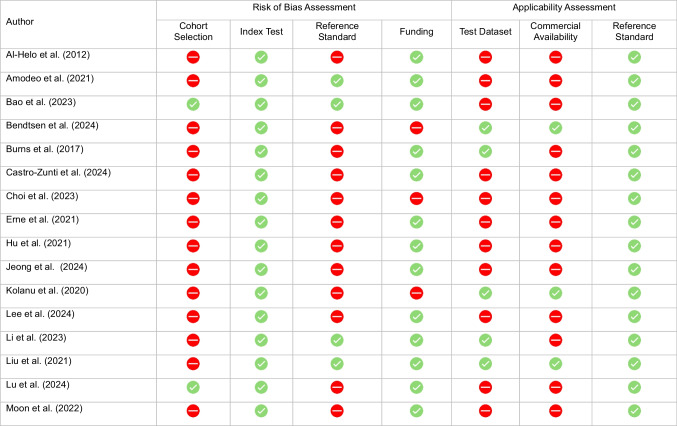

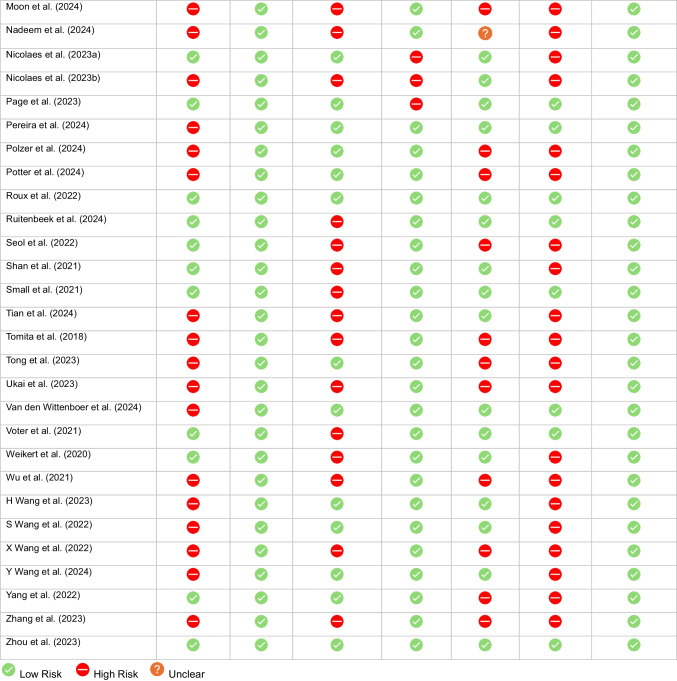
QUADAS-2 Results in Tabular Presentation (Sorted by Author)

### Meta-analyses

A total of 111 contingency tables were extracted from 44 studies. Of these, 21 were combined by summing the individual cells (TP, FN, FP, TN) to create a more comprehensive contingency table for that study. In addition, four studies [38, 46–48] that only reported Genant [32] vertebral body fractures of grades 1–3 were excluded but retained in the descriptive synthesis.

#### Primary analysis

In the primary analysis, diagnostic accuracies were assessed for the following subgroups: (1) selected vs. unselected cohorts, (2) external vs. internal test datasets, and (3) the depth of analysis of different levels: patient-wise vs. region wise (rib-wise vs. vertebra-wise vs. other sample-wise) on the diagnostic accuracy of stand-alone AI .

**Table 4:**
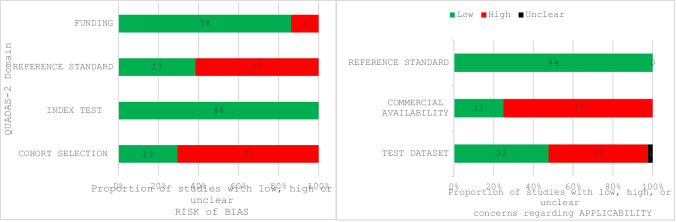
QUADAS-2 Results in Spreadsheet Presentation

#### Diagnostic accuracy of AI stratified by cohort composition: Selected vs. unselected cohorts

**Fig. 2:**
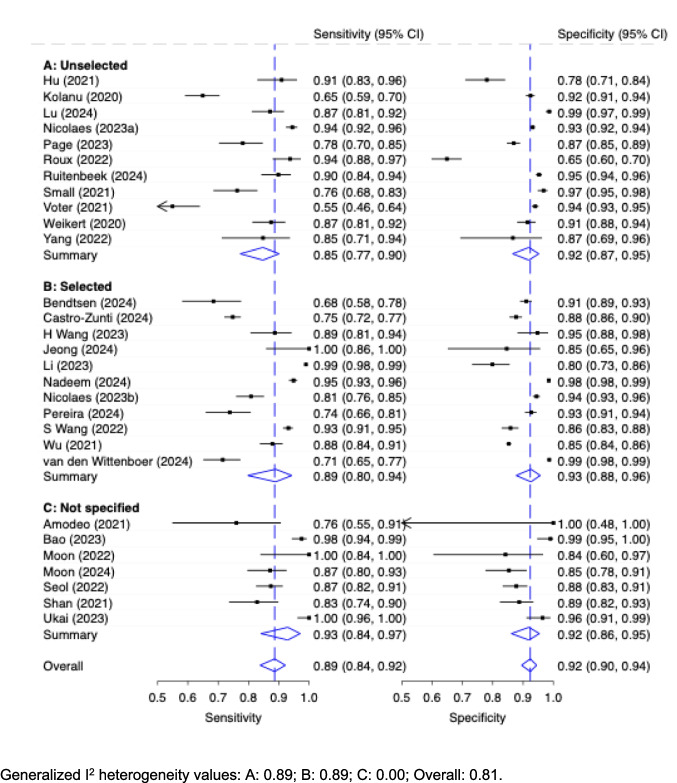
Diagnostic Accuracy with 95% Confidence Interval (CI) of Stand-Alone AI (Patient-Wise Level) by Unselected vs. Selected Cohort Representation

The diagnostic accuracy of stand-alone AI (patient-wise level) with respect to the cohort composition is shown in Fig. 2., with corresponding study-level metrics summarized in Table 5. Meta analysis of ‘unselected’ cohorts (*n* = 11) showed a moderate pooled sensitivity of 0.85 (95% CI: 0.77, 0.90) and good specificity of 0.92 (95% CI: 0.87, 0.95). ‘Selected’ (*n* = 11) cohorts likewise showed a moderate pooled sensitivity of 0.89 (95% CI: 0.80, 0.94) and good specificity of 0.93 (95% CI: 0.88, 0.96). The meta-analysis of unclassified cohorts (*n* = 7) however demonstrated the highest pooled sensitivity (0.93, 95% CI: 0.84.0, 0.97) with a specificity of 0.92 (95% CI: 0.86, 0.95), like the other groups. The values for the generalized I^2^ heterogeneity for ‘unselected’, ‘selected’, ‘not specified’ and overall were 0.89; 0.89; 0.00; 0.81, respectively.

**Table 5:** Study-Level Diagnostic Performance Metrics for Stand-Alone AI (Patient-Wise Level)

Author (Year)	N	TP	FP	TN	FN	Prevalence [%]		Sensitivity [%]		Specificity [%]		PPV [%]		NPV [%]		F1 score
Hu et al. (2021)	252	80	36	128	8	34.9	(29.3 - 41.0)	90.9	(83.1 - 95.3)	78.0	(71.1 - 83.7)	69.0	(60.1 - 76.7)	94.1	(88.8 - 97.0)	0.78
Kolanu et al. (2020)	1570	183	97	1191	99	18.0	(16.1 - 19.9)	64.9	(59.2 - 70.2)	92.5	(90.9 - 93.8)	65.4	(59.6 - 70.7)	92.3	(90.7 - 93.7)	0.65
Lu et al. (2024)	841	148	9	662	22	20.2	(17.6 - 23.1)	87.1	(81.2 - 91.3)	98.7	(97.5 - 99.3)	94.3	(89.5 - 97.0)	96.8	(95.2 - 97.9)	0.91
Nicolaes et al. (2023a)	4810	593	285	3897	35	13.1	(12.1 - 14.0)	94.4	(92.3 - 96.0)	93.2	(92.4 - 93.9)	67.5	(64.4 - 70.6)	99.1	(98.8 - 99.4)	0.79
Page et al. (2023)	1087	107	124	826	30	12.6	(10.8 - 14.7)	78.1	(70.5 - 84.2)	86.9	(84.7 - 88.9)	46.3	(40.0 - 52.8)	96.5	(95.0 - 97.5)	0.58
Roux et al. (2022)	500	119	131	242	8	25.4	(21.8 - 29.4)	93.7	(88.1 - 96.8)	64.9	(59.9 - 69.5)	47.6	(41.5 - 53.8)	96.8	(93.8 - 98.4)	0.63
Ruitenbeek et al. (2024)	2036	150	88	1781	17	8.2	(7.1 - 9.5)	89.8	(84.3 - 93.5)	95.3	(94.2 - 96.2)	63.0	(56.7 - 68.9)	99.1	(98.5 - 99.4)	0.74
Small et al. (2021)	665	109	17	505	34	21.5	(18.5 - 24.8)	76.2	(68.6 - 82.5)	96.7	(94.8 - 98.0)	86.5	(79.5 - 91.4)	93.7	(91.3 - 95.5)	0.81
Voter et al. (2021)	1904	67	106	1676	55	6.4	(5.4 - 7.6)	54.9	(46.1 - 63.5)	94.1	(92.9 - 95.1)	38.7	(31.8 - 46.2)	96.8	(95.9 - 97.6)	0.45
Weikert et al. (2020)	510	139	30	321	20	31.2	(27.3 - 35.3)	87.4	(81.4 - 91.7)	91.5	(88.1 - 93.9)	82.2	(75.8 - 87.3)	94.1	(91.1 - 96.2)	0.85
Yang et al. (2022)	76	39	4	26	7	60.5	(49.3 - 70.8)	84.8	(71.8 - 92.4)	86.7	(70.3 - 94.7)	90.7	(78.4 - 96.3)	78.8	(62.2 - 89.3)	0.88
Bendtsen et al. (2024)	1000	65	81	824	30	9.5	(7.8 - 11.5)	68.4	(58.5 - 76.9)	91.0	(89.0 - 92.7)	44.5	(36.7 - 52.6)	96.5	(95.0 - 97.5)	0.54
Castro-Zunti et al. (2024)	2000	748	123	877	252	50.0	(47.8 - 52.2)	74.8	(72.0 - 77.4)	87.7	(85.5 - 89.6)	85.9	(83.4 - 88.0)	77.7	(75.2 - 80.0)	0.80
H Wang et al. (2023)	192	86	5	90	11	50.5	(43.5 - 57.5)	88.7	(80.8 - 93.5)	94.7	(88.3 - 97.7)	94.5	(87.8 - 97.6)	89.1	(81.5 - 93.8)	0.91
Jeong et al. (2024)	50	24	4	22	0	48.0	(34.8 - 61.5)	100.0	(86.2 - 100.0)	84.6	(66.5 - 93.8)	85.7	(68.5 - 94.3)	100.0	(85.1 - 100.0)	0.92
Li et al. (2023)	2319	2122	35	139	23	92.5	(91.4 - 93.5)	98.9	(98.4 - 99.3)	79.9	(73.3 - 85.2)	98.4	(97.8 - 98.8)	85.8	(79.6 - 90.3)	0.99
Nadeem (et al. (2024)	3231	1007	33	2136	55	32.9	(31.3 - 34.5)	94.8	(93.3 - 96.0)	98.5	(97.9 - 98.9)	96.8	(95.6 - 97.7)	97.5	(96.7 - 98.1)	0.96
Nicolaes et al. (2023b)	1943	240	91	1555	57	15.3	(13.8 - 17.0)	80.8	(75.9 - 84.9)	94.5	(93.3 - 95.5)	72.5	(67.5 - 77.0)	96.5	(95.4 - 97.3)	0.76
Pereira et al. (2024)	899	107	55	699	38	16.1	(13.9 - 18.7)	73.8	(66.1 - 80.3)	92.7	(90.6 - 94.4)	66.0	(58.5 - 72.9)	94.8	(93.0 - 96.2)	0.70
S Wang et al. (2022)	1613	909	90	547	67	60.5	(58.1 - 62.9)	93.1	(91.4 - 94.6)	85.9	(83.0 - 88.4)	91.0	(89.1 - 92.6)	89.1	(86.4 - 91.3)	0.92
Wu et al. (2021)	8051	275	1138	6600	38	3.9	(3.5 - 4.3)	87.9	(83.8 - 91.0)	85.3	(84.5 - 86.1)	19.5	(17.5 - 21.6)	99.4	(99.2 - 99.6)	0.32
van den Wittenboer et al. (2024)	2368	158	29	2118	63	9.3	(8.2 - 10.6)	71.5	(65.2 - 77.0)	98.6	(98.1 - 99.1)	84.5	(78.6 - 89.0)	97.1	(96.3 - 97.7)	0.77
Amodeo et al. (2021)	30	19	0	5	6	83.3	(66.4 - 92.7)	76.0	(56.6 - 88.5)	100.0	(56.6 - 100.0)	100.0	(83.2 - 100.0)	45.5	(21.3 - 72.0)	0.86
Bao et al. (2023)	302	195	1	101	5	66.2	(60.7 - 71.3)	97.5	(94.3 - 98.9)	99.0	(94.7 - 99.8)	99.5	(97.2 - 99.9)	95.3	(89.4 - 98.0)	0.98
Moon et al. (2022)	40	21	3	16	0	52.5	(37.5 - 67.1)	100.0	(84.5 - 100.0)	84.2	(62.4 - 94.5)	87.5	(69.0 - 95.7)	100.0	(80.6 - 100.0)	0.93
Moon et al. (2024)	232	101	17	99	15	50.0	(43.6 - 56.4)	87.1	(79.8 - 92.0)	85.3	(77.8 - 90.6)	85.6	(78.1 - 90.8)	86.8	(79.4 - 91.9)	0.86
Seol et al. (2022)	507	207	33	237	30	46.7	(42.4 - 51.1)	87.3	(82.5 - 91.0)	87.8	(83.3 - 91.2)	86.2	(81.3 - 90.0)	88.8	(84.4 - 92.0)	0.87
Shan et al. (2021)	235	77	16	126	16	39.6	(33.5 - 45.9)	82.8	(73.9 - 89.1)	88.7	(82.5 - 92.9)	82.8	(73.9 - 89.1)	88.7	(82.5 - 92.9)	0.83
Ukai et al. (2023)	205	93	4	108	0	45.4	(38.7 - 52.2)	100.0	(96.0 - 100.0)	96.4	(91.2 - 98.6)	95.9	(89.9 - 98.4)	100.0	(96.6 - 100.0)	0.98

Abbreviations: *NPV* Negative Predictive Value; *PPV* Positive Predictive Value; *TP* True Positive; *FP* False Positive; *TN* True Negative; *FN* False Negative

#### Diagnostic accuracy of AI stratified by test dataset origin: External vs. internal

**Fig. 3:**
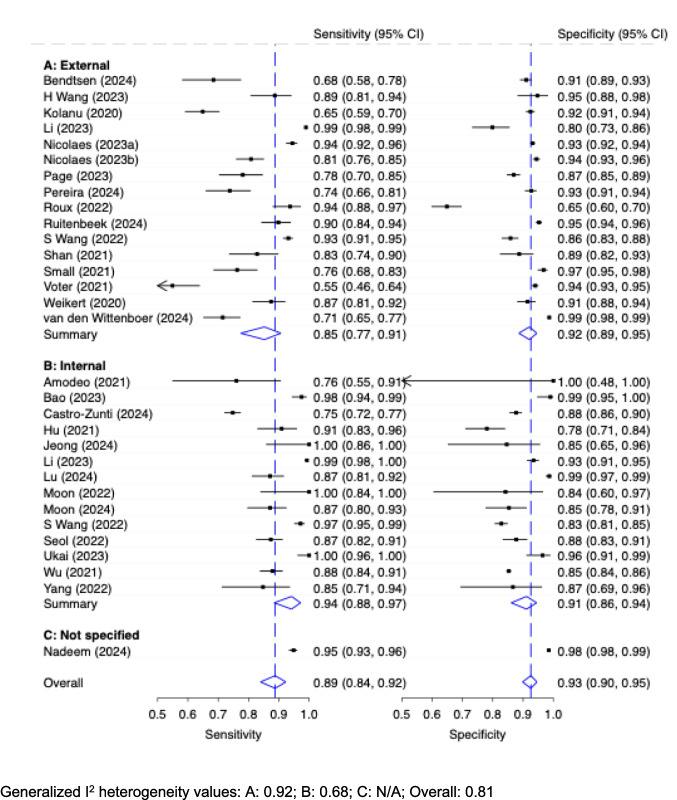
Diagnostic Accuracy with 95% Confidence Interval (CI) of Stand-Alone AI (Patient-Wise Level) by External vs. Internal Test Dataset Origin

The diagnostic accuracy of stand-alone AI (patient-wise level) with respect to the type of test dataset origin is shown in Fig. 3. Studies using external test datasets (*n* = 16) showed a moderate pooled sensitivity of 0.85 (95% CI: 0.77, 0.91) and good specificity of 0.92 (95% CI: 0.89, 0.95). Studies applying internal test datasets (*n* = 14) performed better with respect to the reported sensitivity: They were pooled to have a close to excellent sensitivity of 0.94 (95% CI: 0.88, 0.97) while maintaining a good specificity of 0.91 (95% CI: 0.86, 0.94). The dataset of one study was classified as ‘not specified’. This study reported the highest sensitivity (0.95, 95% CI: 0.93, 0.96) and specificity (0.98, 95% CI: 0.98, 0.99) compared to the other groups. The values for the generalized I^2^ heterogeneity for external, internal, and overall datasets were 0.92; 0.68; 0.81, respectively.

#### Diagnostic accuracy of AI stratified by level of analysis: Patient-wise vs. sample-wise vs. vertebra vs. rib-wise

**Fig. 4:**
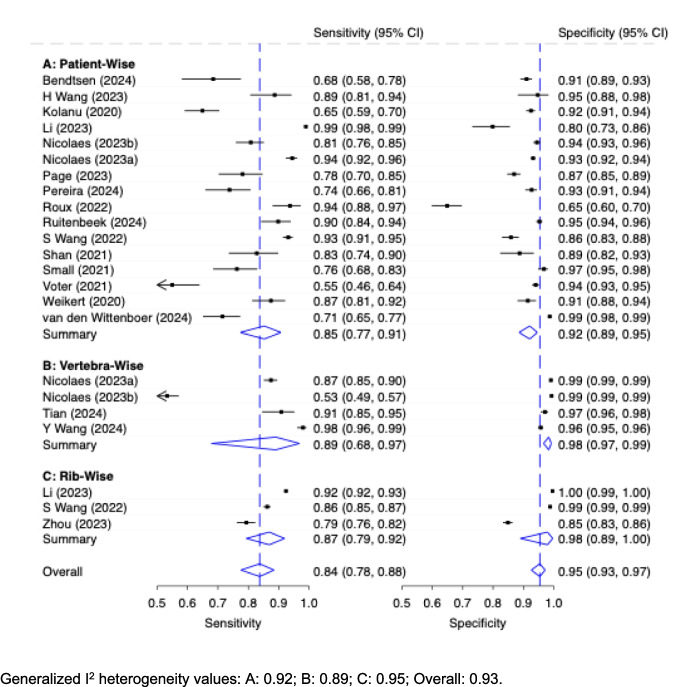
Diagnostic Accuracy with 95% Confidence Interval (CI) of Stand-Alone AI on External Test Datasets by Level of Analysis

The diagnostic accuracy of stand-alone AI on external datasets according to the level of analysis is shown in Fig. 4. External test datasets were stratified in three groups (patient-, vertebra- and rib-wise), whereas internal test datasets were stratified in four groups (patient-wise on the one hand and vertebra-, rib-, and other sample-wise on the other). For external test datasets, ‘patient-wise’ (*n* = 16) analyses showed a pooled moderate sensitivity of 0.85 (95% CI: 0.77, 0.91) and good specificity of 0.92 (95% CI: 0.89, 0.95) whereas meta-analyses of ‘vertebra-wise’ (*n* = 4) and ‘rib-wise’ datasets showed better diagnostic accuracy, sensitivity being 0.89 (95% CI: 0.68, 0.97) and 0.87 (95% CI: 0.79, 0.92) and excellent specificity of 0.98 (95% CI: 0.97, 0.99) and 0.98 (95% CI: 0.89, 1.00). Heterogeneity was high in all groups (I^2^: 0.92; 0.89; 0.95; 0.93 for patient-, vertebra-, rib-wise and overall). This shows that diagnostic accuracy varies depending on the level of analysis, with vertebrae- and rib-wise analyses showing higher specificity.

Pooled diagnostic accuracy measures of internal test datasets are shown in Supplementary Fig. 1. Differences between levels of analysis were like those seen in external datasets. Overall sensitivity of internal datasets was better on all levels compared to external datasets, consistent with the findings for the patient-wise level shown in Fig. 3.

#### Secondary analyses

In the secondary analyses the differences between (1) various CAAI-FDS, (2) anatomical regions and (3) the reader type, e.g., stand-alone AI, human unaided vs. human with AI support were examined.

#### Diagnostic accuracy of AI stratified by commercial availability

All studies evaluating CAAI-FDS were classified as using external test datasets by definition, as these systems were assessed independently of their developers.

**Fig. 5:**
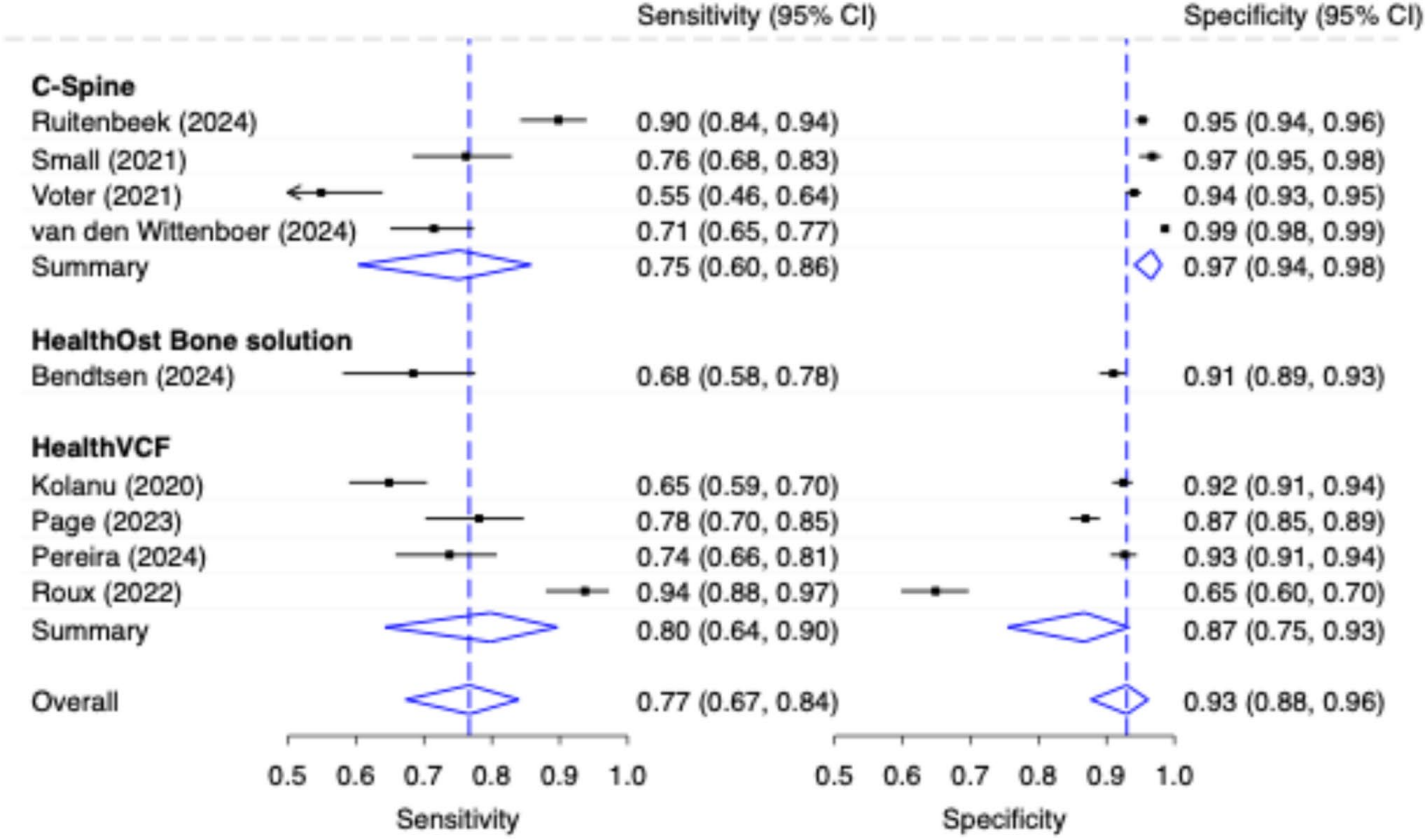
Diagnostic Accuracy with 95% Confidence Interval (CI) of Stand-Alone AI (Patient-Wise Level) by Commercially Available AI Fracture Detection Solution

The diagnostic accuracy of stand-alone AI at the patient-wise level according to different CAAI-FDS is shown in Fig. 5. ‘C-Spine’ (*n* = 4) achieved a pooled low to moderate sensitivity of 0.75 (95% CI: 0.60, 0.86) and excellent specificity of 0.97 (95% CI: 0.94, 0.98); whereas studies evaluating ‘HealthVCF’ (*n* = 4) demonstrated a higher sensitivity (0.80, 95% CI: 0.64, 0.90) and lower specificity (0.87, 95% CI: 0.75, 0.93). ‘HealthOst Bone solution’ was evaluated in only one study which reported a poor sensitivity of 0.68 (95% CI: 0.58, 0.78) and good specificity of 0.91 (95% CI: 0.89, 0.93). The values for the generalized I^2^ heterogeneity were 0.92; 0.00; 0.94 for ‘C-Spine’, ‘HealthVCF’ and overall.

**Fig. 6:**
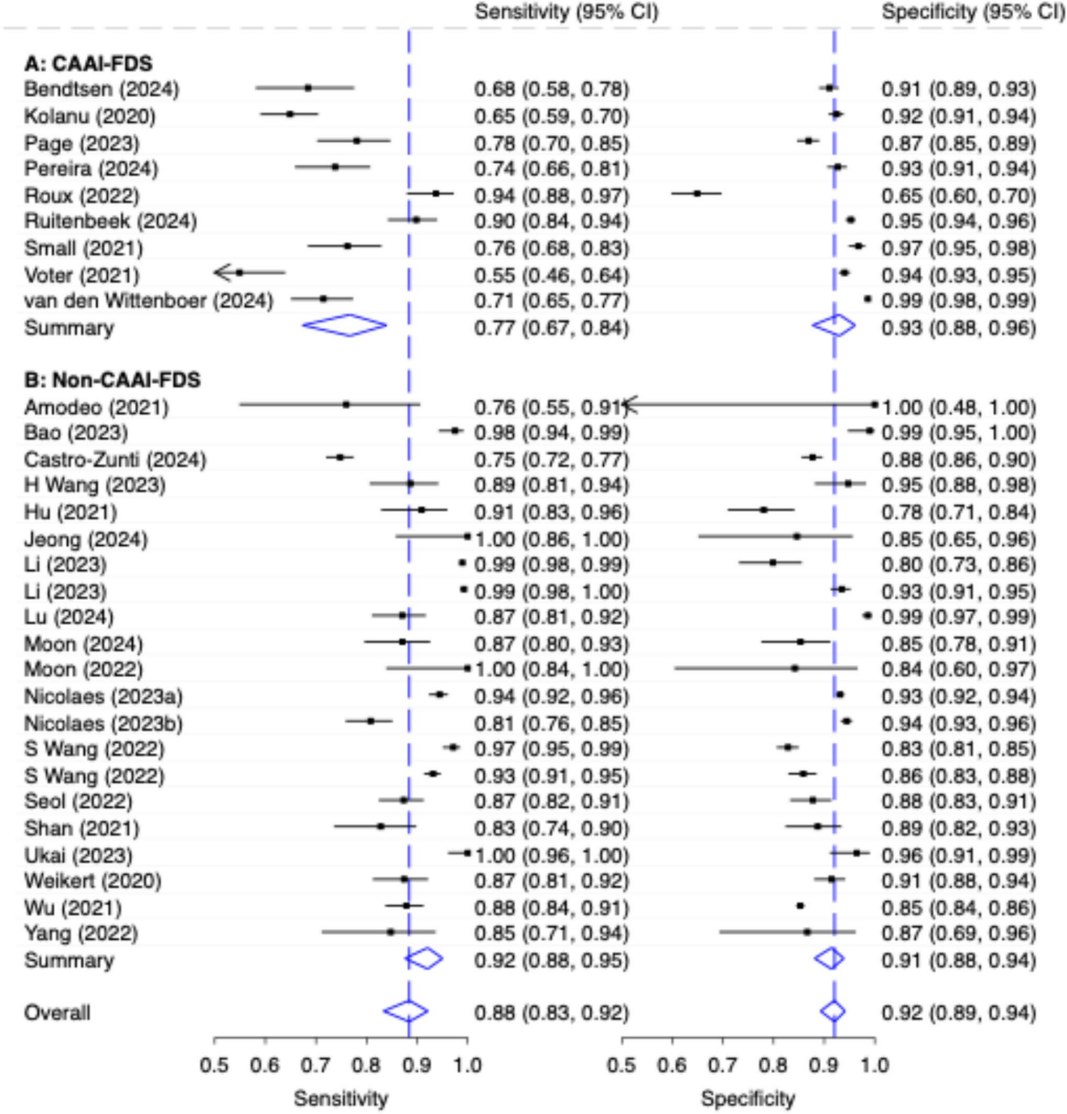
Diagnostic Accuracy with 95% Confidence Interval (CI) of Stand-Alone AI (Patient-Wise Level) by Commercially Available AI Fracture Detection Solution vs. Non- Commercially Available AI Fracture Detection Solution

The pooled sensitivity for all CAAI-FDS was moderate (0.77, 95% CI 0.67, 0.84) and the pooled specificity was good (0.93, 95% CI: 0.88, 0.96). Non-CAAI-FDS studies (stand-alone AI; patient-wise, *n* = 18) achieved a higher pooled sensitivity of 0.88 (95% CI: 0.83–0.92) at a similar specificity of 0.92 (95% CI: 0.89–0.94), see Fig. 6. Two of these studies included evaluations using both external and internal test datasets [49, 50]. The values for the generalized I^2^ heterogeneity for CAAI-FDS, Non-CAAI-FDS, and overall were 0.94; 0.71; 0.80, respectively.

#### Diagnostic accuracy of AI stratified by anatomical region

**Fig. 7:**
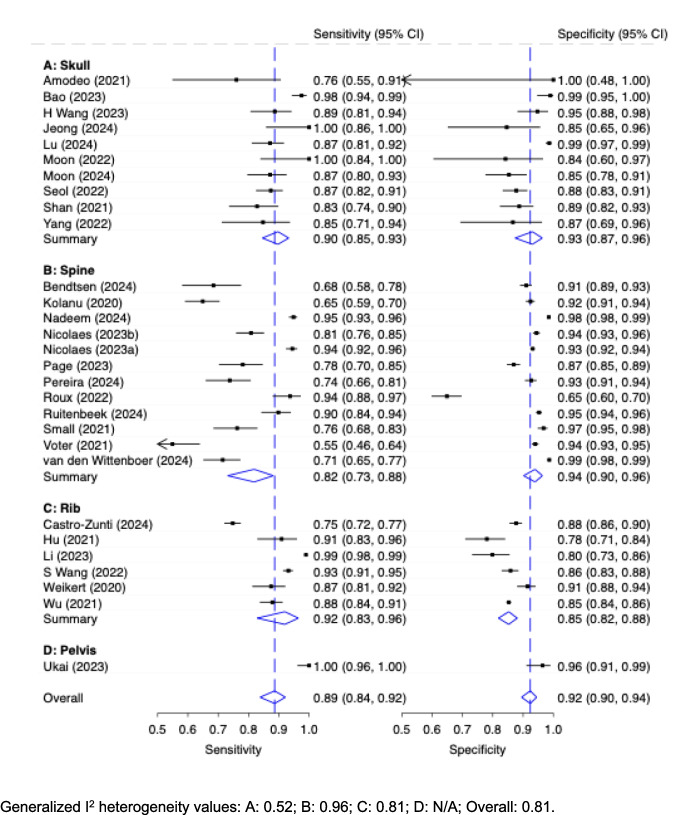
Diagnostic Accuracy with 95% Confidence Interval (CI) of Stand-Alone AI (Patient-Wise Level) by Anatomical Region

The diagnostic accuracy of stand-alone AI with respect to the different anatomical regions is shown in Fig. 7. Studies assessing the spine showed the lowest sensitivity with a pooled moderate value of 0.82 (95% CI: 0.73, 0.88) but the highest, close to excellent specificity (0.94, 95% CI: 0.90, 0.96). The best performance with respect to sensitivity was observed from studies evaluating the ribs with a pooled good sensitivity (0.92, 95% CI: 0.83, 0.96) and moderate specificity (0.85, 95% CI: 0.82, 0.88). Studies evaluating the skull performed similarly, with a pooled good sensitivity of 0.90 (95% CI: 0.85, 0.93) and a pooled good specificity of 0.93 (95% CI: 0.87, 0.96). Only one study evaluated ‘pelvis’. Heterogeneity was moderate to high (I^2^: 0.52; 0.96; 0.81; 0.81 for skull, spine, rib, overall).

#### Diagnostic accuracy of different raters: Stand-alone AI, human-aided, and human unaided

**Fig. 8:**
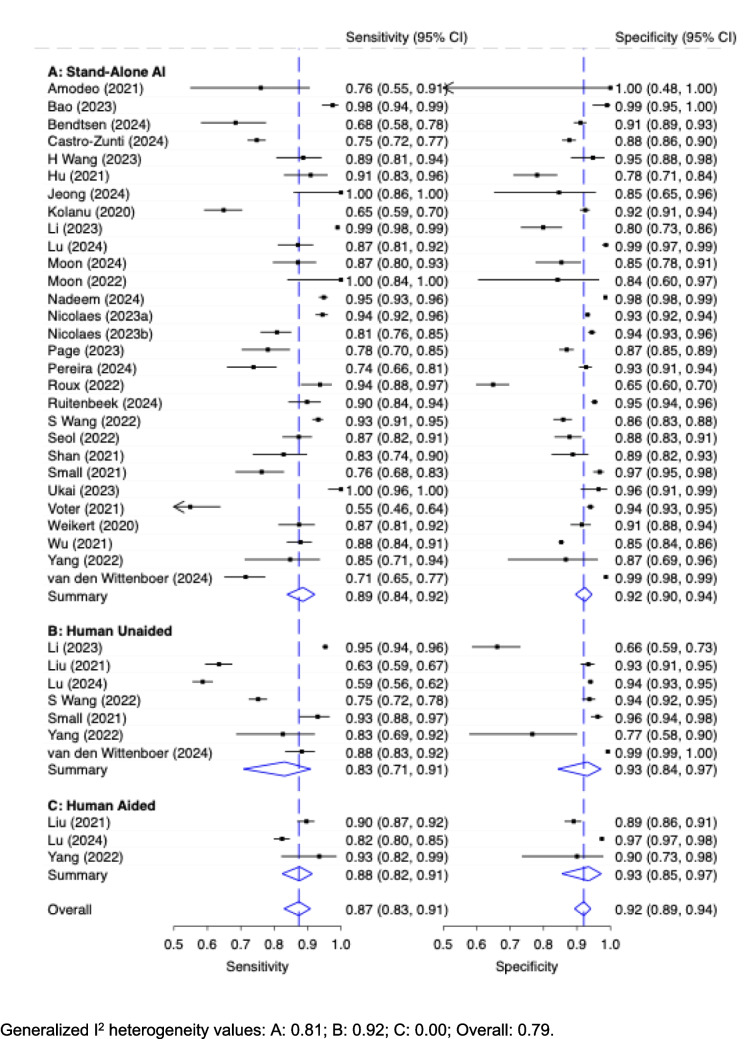
Diagnostic Accuracy with 95% Confidence Interval (CI) of Reader Type (Patient-Wise Level). Generalized I^2^ heterogeneity values: A: 0.81; B: 0.92; C: 0.00; Overall: 0.79

The diagnostic accuracy of different reader type (patient-wise level) is shown in Fig. 8. Differences between ‘Stand-Alone AI’ (*n* = 29) and ‘Human unaided’ were small, however AI performed slightly better with respect to sensitivity: ‘Stand-Alone AI’ showed a pooled close to good sensitivity of 0.89 (95% CI: 0.84, 0.92) and specificity of 0.92 (95% CI: 0.90, 0.94). whereas ‘Human Unaided’ (*n* = 7) performed a pooled slightly worse sensitivity of 0.83 (95% CI: 0.71, 0.91) and comparable specificity of 0.93 (95% CI: 0.84, 0.97). ‘Human Aided’, only evaluated in three studies, showed a sensitivity of 0.88 (95% CI: 0.87, 0.92) with a specificity of 0.93 (95% CI: 0.85, 0.97). The values for the generalized I^2^ heterogeneity were as followed for ‘Stand Alone AI’, ‘Human unaided’, ‘Human aided’ and overall: 0.81; 0.92; 0.00; 0.79.

A detailed breakdown of heterogeneity measures for each analysis is provided in Supplement 4. Additional analyses are presented in Supplement 5, with corresponding study-level performance metrics provided in aligned supplementary tables.

## Discussion

This comprehensive DTA systematic review with meta-analysis synthesizes evidence from 44 studies evaluating AI solutions for fracture detection on CT images. Overall, pooled estimates for stand-alone AI demonstrate moderate to good diagnostic sensitivity and good specificity, consistent with findings from prior reviews [14, 15, 18, 19]. Distinctively, our study is the first to systematically examine how methodological factors – such as cohort selection and the use of independent test datasets – influences diagnostic performance. Additionally, we provide a detailed stratification by anatomical region and commercial availability – providing a more granular understanding of the real-world applicability of AI solutions.

Nearly all included studies (40 out of 44) were rated as having at least a moderate risk of bias, with the most frequent concern being non-transparent or selective patient inclusion. Studies either failed to sufficiently report patient characteristics or applied overly restrictive inclusion criteria, limiting the generalizability of their findings. This concern has previously been raised by Cohen and others [21], who noted that stringent case selection may lead to inflated performance estimates. Our findings support this notion: studies using representative, unselected patient cohorts reported a pooled sensitivity of 0.85 (95% CI: 0.77, 0.90). In contrast, studies with clearly selected cohorts showed higher sensitivity (0.89, 95% CI: 0.80, 0.94), and studies that did not report their selection process performed best, with a pooled sensitivity of 0.93 (95% CI: 0.84, 0.97). Notably, this ‘not specified’ cohort subgroup (*n* = 7) also showed an absence of between-study heterogeneity (*I*^2^ = 0.00) despite its high pooled performance, which might be explained by non-transparent cohort definitions and/or selectively reported cohorts with inflated sensitivity estimates. This lack of transparent reporting likely reflects deviations from recommended methodological standards, resulting in artificially elevated sensitivity estimates in these cohorts. Specificity, by contrast, remained relatively consistent across groups.

With respect to applicability, a key finding from our analysis is the difference in diagnostic performance of internal and external test datasets. Only 15 of 44 studies employed external test datasets for testing the AI algorithms. Interestingly, studies that relied on external test datasets demonstrated a lower pooled sensitivity of 0.85 (95% CI: 0.77, 0.91) compared to those using internal test datasets, which were pooled to have a sensitivity of 0.94 (95% CI: 0.88, 0.97). Specificity was comparable between the two groups. This discrepancy underlines the hypothesis [21] that algorithms perform optimally when tested on the same dataset or under similar conditions to the development phase, as seen with internal test datasets. Internal datasets typically consist of images from the same hospital, imaging protocols, and equipment, which may lead to an overestimation of performance when generalized to new, unseen datasets. In contrast, external test datasets, which involve images from different hospitals, equipment, and patient populations, likely present a broader range of conditions that the algorithms have not encountered during training, reducing their ability to generalize effectively. This finding underscores the importance of external test datasets as a critical step for assessing the real-world applicability of AI solutions. It also highlights that performance metrics based on internal testing may not accurately reflect how the algorithms will perform when deployed in clinical settings, where variability in imaging conditions and patient demographics is inevitable.

These concerns regarding bias in the selection of cohorts and test datasets are not only methodological and affect overall diagnostic accuracy in real-world settings but also raise important ethical considerations. Several authors [51, 52] have voiced concerns over how AI models trained on non-representative datasets may amplify biases, leading to poorer performance on underrepresented populations. This could contribute to health disparities, particularly if AI systems are deployed in clinical settings without thorough evaluation across diverse patient groups.

A likely explanation for the limited use of external datasets is the persistent challenge of data sharing across institutions, often due to privacy concerns, regulatory restrictions, or lack of infrastructure for secure collaboration. This is a well-recognized issue in the field of medical AI development. Federated learning presents a promising alternative. By allowing decentralized training of AI models while preserving data privacy, federated learning may help overcome institutional barriers and enable broader validation efforts across diverse clinical settings. Synthetic data has been suggested as an alternative to supplement real data, particularly in situations where obtaining diverse or sufficiently large datasets is difficult; however, this should be done with caution, as it may inadvertently reinforce biases and fail to fully represent clinical realities.

A further key limitation to applicability identified in our analysis is that only eleven of the 44 studies assessed AI solutions which were commercially available. These commercially available systems generally demonstrated lower diagnostic sensitivity (0.77, 95% CI: 0.67, 0.84) compared to the non-commercially available AI solutions (0.88, 95% CI: 0.83–0.92), with specificity being comparable across both groups. One potential explanation for this difference is that all studies identified to test commercially available solutions used external data sets whereas studies evaluating non commercially available solutions used both external and internal datasets. Commercially available systems were only tested independently from their developers, meaning they may have been exposed to real-world variables do not present in more controlled research settings. These variables, such as system integration, user familiarity, and the diversity of clinical data, could potentially affect the system’s performance in ways not accounted for during the development phase. As a result, these systems may underperform compared to those evaluated in more controlled environments, where factors such as dataset consistency and developer oversight are more tightly managed. Of the three different commercially available fracture detection solutions which were evaluated, the pooled sensitivities were poor to moderate, whereas specificities were good to excellent. This may seem paradoxical, given that in real-world clinical settings and under current regulatory frameworks, AI is intended solely to support human readers. Therefore, prioritizing sensitivity would be more logical, as the final diagnosis is always made by human readers, with AI serving primarily to assist in detecting potential cases that might otherwise be overlooked.

In addition to dataset composition and validation strategy, limited reporting of precision-oriented performance metrics represents a major barrier to assessing real-world robustness of AI fracture detection systems. Metrics such as PPV, F1-score, precision–recall analyses (e.g., AUPRC), and detection-oriented measures of false positives (e.g., false positives per scan/FROC) were inconsistently reported across studies and could not be synthesized quantitatively. Consequently, models may retain favorable sensitivity and specificity (even in externally validated or multicenter settings) while still producing a clinically prohibitive false-positive burden under domain shift. This limitation, together with dataset bias in proof-of-concept studies, likely contributes to the observed performance gap between commercially available and experimental AI solutions, as commercial systems are typically optimized for robustness across heterogeneous clinical environments, often at the expense of sensitivity and precision-related endpoints.

In line with similar reviews, our study highlights the variability in diagnostic performance depending on the anatomical region being studied. The highest sensitivity was observed in studies evaluating rib fractures (0.92, 95% CI: 0.83, 0.96), followed by skull fractures (0.90, 95% CI: 0.85, 0.93) and spine `fractures (0.82, 95% CI: 0.73, 0.88). However, this was accompanied by an inverse relationship with specificity. These differences may be attributable to either the intrinsic characteristics of the anatomical regions or the algorithm’s potential emphasis on optimizing sensitivity, a common trade-off given the inherent mathematical relationship between sensitivity and specificity.

Another critical question is how AI compares to human experts. Most studies only evaluated AI performance, which limits generalizability due to the variability in dataset difficulty and composition. With the limited number of studies available, our review indicates that current AI systems perform comparably to or slightly better than radiologists in fracture detection on CT. However, no stratification was conducted according to the experience of the radiologists, as this information was often not provided. In pooled analysis, radiologists achieved approximately 83% sensitivity and 93% specificity, while AI performed with 89% sensitivity and 92% specificity. This suggests that AI is slightly more accurate in detecting fractures, while radiologists had a marginally lower false-positive rate. However, the differences were small, aligning with other studies reporting non-inferior performance of AI compared to radiologists [14, 15, 18]. This review also explored human-AI collaboration, though only a few studies addressed this. Interestingly, in three studies where radiologists used AI assistance, the combined human and AI reading showed a sensitivity of 0.90 (95% CI: 0.87, 0.92) which is close to the stand-alone AI but better than unaided human readers. It is important to note, however, that the number of direct comparison studies is limited.

The studies included in this analysis further differed in granularity and level of analysis. While most assessed diagnostic performance at the patient-wise level, others evaluated individual structures such as ribs or vertebrae. If a model relies on spurious correlations, it may reproduce these cues in negative cases, resulting in increased false positives and reduced precision/F1. Patient-wise aggregation can amplify this effect, as classifying a patient as positive based on any detected finding makes overall performance highly sensitive to even a small number of spurious detections, thereby increasing false-positive burden compared with structure-level assessment.

These findings suggest that structure-level assessment may provide more localized outputs and facilitate interpretation of where false-positive detections arise, whereas patient-wise assessment may be more susceptible to clinically burdensome overcalling when spurious cues are present. Some recent models provide visualization of regions of interest or heatmaps indicating where the algorithm focuses, which can support interpretability by helping readers judge whether predictions are driven by plausible anatomical features rather than non-target cues. Importantly, such visualizations should not be interpreted as improving specificity per se; rather, they complement reporting of precision-oriented endpoints (e.g., precision/PPV) and recall/sensitivity, ideally summarized using F1-score or precision–recall analyses, to better characterize clinical usability and robustness under domain shift.

Our review identified only one study which evaluated the clinical impact of AI-based fracture detection systems. In that study, Ruitenbeek et al. reported a reduction of 16 minutes in the time to diagnosis of cervical spine fractures following the introduction of AI [53]. The extent to which such time savings translate into improved patient-relevant outcomes remains incompletely understood.

While downstream patient-relevant outcomes remain an important benchmark, it should be noted that for computer-aided triage and notification (CADt) systems, time-to-diagnosis or workflow efficiency gains can constitute a clinically meaningful effectiveness endpoint aligned with the intended use of prioritizing time-sensitive findings. Such endpoints have also been considered in regulatory and reimbursement evaluation frameworks. Nevertheless, for AI-based fracture detection, additional prospective evidence is needed to clarify when observed time savings translate into patient-relevant benefits.

The lack of robust clinical evaluation is striking given the increasing adoption of AI tools in routine practice. It is essential to demonstrate the effect on relevant clinical endpoints – such as fewer missed fractures, decrease in complication rates, reduction in re-admission or hard endpoints such as mortality rates. This will require well-designed prospective trials, ideally randomized or cluster-randomized, that compare AI-assisted workflows to standard practice while accounting for the clinical context typically available to radiologists. In parallel, monitoring during clinical use will be essential to ensure safety and performance, and cost-effectiveness analyses will be crucial for evaluating the economic viability of integrating AI into real-world healthcare systems.

## Strengths and limitations

This systematic review provides the most comprehensive assessment to date of AI-based fracture detection on CT, including both peer-reviewed and commercial solutions. Strengths include adherence to PRISMA-DTA guidelines, a broad search strategy with citation chasing, and robust subgroup analyses by cohort selection, test dataset origin, level of analysis, CAAI-FDS, anatomical region, and different rater types. Risk of bias was rigorously assessed using a tailored QUADAS-2 tool.

However, several limitations must be acknowledged. Most included studies had at least moderate risk of bias, often due to selective patient inclusion and limited transparency.

Although external testing was rare, raising concerns about generalizability, limited and heterogeneous reporting of precision-oriented and false-positive burden metrics represents an equally important barrier to assessing real-world usability. While study-level PPV and F1 scores could be derived from available contingency tables, these measures could not be synthesized quantitatively due to inconsistent reporting and their strong dependence on study-specific prevalence. As a result, algorithms may appear robust based on sensitivity and specificity in internal or even external cohorts while still generating a clinically prohibitive false-positive workload under domain shift.

Reporting was frequently incomplete or inconsistent across studies, limiting the ability to standardize key methodological characteristics (e.g. reference standards, reader protocols) and constraining quantitative synthesis of clinically relevant endpoints like false-positive burden and precision-oriented metrics. Substantial clinical and methodological heterogeneity (e.g. different anatomical targets, units of analysis, and study design) reduces comparability and necessitates cautious interpretation of pooled estimates.

Finally, few studies assessed clinical outcomes, restricting conclusions about real-world impact beyond diagnostic performance.

## Conclusions

This systematic review and meta-analysis identified several key factors influencing the diagnostic accuracy and clinical applicability of AI-based fracture detection solutions. Studies with preselected cohorts tended to report higher sensitivities. Diagnostic accuracy was slightly lower when AI algorithms were evaluated using external test datasets, i.e., data originating from a different context than that used for training. Commercially available AI solutions slightly underperformed to experimental, non-commercial ones. These findings show the limited generalizability of available studies and underscore the need for transparent reporting of methodological practices. Rigorous independent accuracy assessments are necessary to better understand real-world applicability of AI tools. To ensure more robust and representative model development it is crucial to explore and implement methods that optimize data availability for training and facilitate secure data sharing, such as federated learning.

Future research should further prioritize prospective clinical trials and ongoing post-market surveillance to establish not only diagnostic accuracy but also the clinical utility and safety of AI-driven diagnostic systems in clinical practice.

## Supplementary information

ESM 1 (DOCX 539 KB)

ESM 2 (DOCX 34.8 KB)

## Data Availability

The data supporting the results of this study are available on reasoned request from the corresponding author.

## Declarations

### AI assisted technologies

ChatGPT (OpenAI) was used to improve the grammar and overall readability of the manuscript. The authors reviewed and approved all edits to ensure accuracy and consistency with the intended meaning.
