# Supplementary material for: Artificial intelligence for fracture detection on computed tomography: a comprehensive systematic review and meta-analysis of diagnostic test accuracy in non-commercial and commercial solutions
Source: Emerg Radiol. 2026 Feb 7;33(2):351–75. doi: 10.1007/s10140-026-02437-7 (PMC13079534; doi:10.1007/s10140-026-02437-7)
Supplement: Supplementary file 1 — (DOCX 539 KB) [file 10140_2026_2437_MOESM1_ESM.docx]

# Supplement

## Supplement 1. Search Syntax for the different Databases

### Database 1. Embase via Ovid

| **Artificial intelligence (AI)** | 1 | exp artificial intelligence/ or exp artificial neural network/ or exp machine learning/ or exp large language model/ |
| --- | --- | --- |
|  | 2 | (AI or algorithm* or artificial intelligence or artificial neural network* or CNN or CNN-based or computer-aided detection or computer-aided diagnosis or convolutional network* or deep belief network* or deep convolutional neural network* or deep learning or deep learning algorithm* or deep learning system* or expert system* or feed-forward neural network* or intelligent system* or machine learning or R-CNN or recurrent neural network* or reinforcement learning or large language model* or LLM).ti,ab. |
|  | 3 | 1 or 2 |
| **CT** | 4 | exp computer assisted tomography/ or exp CT scanner/ |
|  | 5 | (CT or CT scan* or computed tomograph* or computerized tomograph*).ti,ab. |
|  | 6 | 4 or 5 |
| **Fracture** | 7 | exp fracture/ |
|  | 8 | fractur*.ti,ab. |
|  | 9 | 7 or 8 |
|  | 10 | 3 (AI) and 6 (CT) and 9 (Fracture) |
|  | 11 | limit 10 to yr="2010 -Current" |

### Database 2. MEDLINE via Ovid

| **AI** | 1 | exp Artificial Intelligence/ or exp Neural Networks, Computer/ |
| --- | --- | --- |
|  | 2 | (AI or algorithm* or artificial intelligence or artificial neural network* or CNN or CNN-based or computer-aided detection or computer-aided diagnosis or convolutional network* or deep belief network* or deep convolutional neural network* or deep learning or deep learning algorithm* or deep learning system* or expert system* or feed-forward neural network* or intelligent system* or machine learning or R-CNN or recurrent neural network* or reinforcement learning or large language model* or LLM).ti,ab. |
|  | 3 | 1 or 2 |
| **CT** | 4 | exp Tomography, X-Ray Computed/ or exp Tomography Scanners, X-Ray Computed/ |
|  | 5 | (CT or CT scan* or computed tomograph* or computerized tomograph*).ti,ab. |
|  | 6 | 4 or 5 |
| **Fracture** | 7 | exp Fractures, Bone/ |
|  | 8 | fractur*.ti,ab. |
|  | 9 | 7 or 8 |
|  | 10 | 3 (AI) and 6 (CT) and 9 (Fracture) |
|  | 11 | limit 10 to yr="2010 -Current" |

### Database 3. Cochrane Library

| **AI** | 1 | MeSH descriptor: [Artificial Intelligence] explode all trees |
| --- | --- | --- |
|  | 2 | MeSH descriptor: [Neural Networks, Computer] explode all trees |
|  | 3 | AI or algorithm* or "artificial intelligence" or (artificial NEXT neural NEXT network*) or CNN or CNN-based or "computer-aided detection" or "computer-aided diagnosis" or (convolutional NEXT network*) or (deep NEXT belief NEXT network*) or (deep NEXT convolutional NEXT neural NEXT network*) or "deep learning" or (deep NEXT learning NEXT algorithm*) or (deep NEXT learning NEXT system*) or (expert NEXT system*) or (feed-forward NEXT neural NEXT network*) or (intelligent NEXT system*) or "machine learning" or R-CNN or (recurrent NEXT neural NEXT network*) or "reinforcement learning" or (large NEXT language NEXT model*) or LLM |
|  | 4 | #1 OR #2 OR #3 |
| **CT** | 5 | MeSH descriptor: [Tomography, X-Ray Computed] explode all trees |
|  | 6 | MeSH descriptor: [Tomography Scanners, X-Ray Computed] explode all trees |
|  | 7 | CT or CT scan* or computed tomograph* or computerized tomograph* |
|  | 8 | #5 OR #6 OR #7 |
| **Fracture** | 9 | MeSH descriptor: [Fractures, Bone] explode all trees |
|  | 10 | fractur* |
|  | 11 | #9 OR #10 |
|  | 12 | #4 (AI) AND #8 (CT) AND #11 (Fracture) |

### Database 4. Web of Science

| **AI** | 1 | TS=(AI OR algorithm* OR “artificial intelligence” OR “artificial neural network*” OR “machine learning” OR CNN OR CNN-based OR “computer-aided detection” OR “computer-aided diagnosis” OR “convolutional network*” OR “deep belief network*” OR “deep convolutional neural network*” OR “deep learning” OR “deep learning algorithm*” OR “deep learning system*” OR “expert system*” OR “feed-forward neural network*” OR “intelligent system*” OR R-CNN OR “recurrent neural network*” OR “large language model” OR LLM) |
| --- | --- | --- |
| **CT** | 2 | TS=(CT OR “CT scan*” OR “computed tomograph*” OR “computerized tomograph*”) |
| **Fracture** | 3 | TS=(Fractur*) |
|  | 4 | #1 (AI) AND #2 (CT) AND #3 (Fracture) |

### Database 5. Google Scholar

| **AI** | 1 | ("Artificial Intelligence" OR "Machine Learning" OR "Deep Learning" OR "CNN" OR "Computer-Aided Detection" OR "Convolutional Neural Network" OR "Large Language Models") |
| --- | --- | --- |
| **Fracture** | 2 | ("Fracture Detection" OR "Fracture Diagnosis" OR "Bone Fracture" OR "Orthopedic Trauma") |
| **CT** | 3 | ("Computed Tomography" OR "CT Images") |
|  | 4 | #1 (AI) AND #2 (Fracture) AND #3 (CT) |
|  |  | Included most relevant: 200 |

### Database 6. IEEE Xplore via Google Scholar

| **AI** | 1 | ("Artificial Intelligence" OR "Machine Learning" OR "Deep Learning" OR "CNN" OR "Computer-Aided Detection" OR "Convolutional Neural Network" OR "Large Language Models") |
| --- | --- | --- |
| **Fracture** | 2 | ("Fracture Detection" OR "Fracture Diagnosis" OR "Bone Fracture" OR "Orthopedic Trauma") |
| **CT** | 3 | ("Computed Tomography" OR "CT Images") |
|  |  | #1 (AI) AND #2 (Fracture) AND #3 (CT) |
|  |  | site:ieeexplore.ieee.org |

## Supplement 2. Data extraction table

| Variable name | Variable label |
| --- | --- |
| no_uniquie | Unique ID |
| study_id | Study ID |
| author | First Author |
| year | Publication Year |
| country | Country of Study |
| V_algorithm | AI Algorithm |
| product_name | Product Name |
| reference_standard | Reference Standard |
| funding | Industrial Funding |
| anatomical_region | Anatomical Region |
| anatomical_subregion | Anatomical Subregion |
| bias_risk | Risk of Bias |
| cohort_representation | Cohort Representation |
| reader_type | Reader Type |
| V_information | Information |
| inclusion_quant_analysis | Inclusion |
| analysis_level | Level of Analysis |
| dataset_origin | Dataset |
| pooled | Data Pooled |
| n_analysis_units | Number of Analysis Units |
| n_fractures | Number of Fractures |
| n_nonfractures | Number of Non-Fractures |
| true_positives | True Positives |
| false_positives | False Positives |
| false_negatives | False Negatives |
| true_negatives | True Negatives |
| sensitivity | Sensitivity |
| specificity | Specificity |

## Supplement 3. Signalling Questions for Risk of Bias and Applicability

#### Risk of Bias Assessment Signalling Questions (SQ)

*Cohort Selection (CS)*

CS-SQ-1: Was sufficient patient data reported?

CS-SQ-2: Was a random, unselected patient cohort included?

*Index Test (IT)*

IT-SQ-1: Were the index test results interpreted without knowledge of the reference standard results?

*Reference Standard (RS)*

RS-SQ-1: Was the reference standard determined by expert consensus?

RS-SQ-2: Were the reference standard results interpreted independently of the index test results?

*Funding (F)*

F-SQ-1: Was the study funded by industrial sources?

#### Applicability assessment (A) Signalling Questions

*Test Dataset (TD)*

TD-SQ-A-1: Did the study use an external or internal test dataset to test the AI-FD solution?

*Commercial Availability (CA)*

CA-SQ-A-1: Is the AI-FD solution commercially available?

*Reference Standard (RS)*

RS-SQ-A-1: Are there concerns that the target condition as defined by the reference standard does not align with the research question?

### Answers Signaling Questions in Tabular Presentation (Sorted by Author)

|  | Risk of Bias Assessment | | | | | | | | | | | Applicability Assessment | | |
| --- | --- | --- | --- | --- | --- | --- | --- | --- | --- | --- | --- | --- | --- | --- |
|  | Cohort Selection | | | Index Test | | Reference Standard | | | Funding | | Overall Assessment | Test Dataset | Commercial Availability | Reference Standard |
| Author | CS-SQ-1 | CS-SQ-2 | CS-Overall | IT-SQ-1 | IT-Overall | RS-SQ-1 | RS-SQ-2 | RS-Overall | F-SQ-1 | F-Overall |  | TD-SQ-A-1 | CA-SQ-A-1 | RS-SQ-A-1 |
| Al-Helo et al. (2012) | No | N/A | High | Yes | Low | No | Yes | High | No | Low | Moderate | High | High | Low |
| Amodeo et al. (2021) | No | N/A | High | Yes | Low | Yes | Yes | Low | No | Low | Moderate | High | High | Low |
| Bao et al. (2023) | Yes | N/A | Low | Yes | Low | Yes | Yes | Low | No | Low | Low | High | High | Low |
| Bendtsen et al. (2024) | Yes | No | High | Yes | Low | No | Yes | High | Yes | High | High | Low | Low | Low |
| Burns et al. (2017) | Yes | No | High | Yes | Low | No | No | High | No | Low | Moderate | Low | High | Low |
| Castro-Zunti et al. (2024) | Yes | No | High | Yes | Low | No | Yes | High | No | Low | Moderate | High | High | Low |
| Choi et al. (2023) | No | Yes | High | Yes | Low | No | Yes | High | Yes | High | High | High | High | Low |
| Erne et al. (2021) | No | No | High | Yes | Low | No | Yes | High | No | Low | Moderate | High | High | Low |
| Hu et al. (2021) | No | Yes | High | Yes | Low | No | Yes | High | No | Low | Moderate | High | High | Low |
| Jeong et al. (2024) | No | No | High | Yes | Low | No | Yes | High | No | Low | Moderate | High | High | Low |
| Kolanu et al. (2020) | No | Yes | High | Yes | Low | Yes | No | High | Yes | High | High | Low | Low | Low |
| Lee et al. (2024) | No | No | High | Yes | Low | No | Yes | High | No | Low | Moderate | High | High | Low |
| Li et al. (2023) | Yes | No | High | Yes | Low | Yes | Yes | Low | No | Low | Moderate | Low | High | Low |
| Liu et al. (2021) | Yes | No | High | Yes | Low | Yes | Yes | Low | No | Low | Moderate | Low | Low | Low |
| Lu et al. (2024) | Yes | Yes | Low | Yes | Low | No | Yes | High | No | Low | Moderate | High | High | Low |
| Moon et al. (2022) | No | N/A | High | Yes | Low | No | Yes | High | No | Low | Moderate | High | High | Low |
| Moon et al. (2024) | No | N/A | High | Yes | Low | No | Yes | High | No | Low | Moderate | High | High | Low |
| Nadeem et al. (2024) | Yes | No | High | Yes | Low | No | Yes | High | No | Low | Moderate | Unclear | High | Low |
| Nicolaes et al. (2023a) | Yes | Yes | Low | Yes | Low | Yes | Yes | Low | Yes | High | Moderate | Low | High | Low |
| Nicolaes et al. (2023b) | Yes | No | High | Yes | Low | No | Yes | High | Yes | High | High | Low | High | Low |
| Page et al. (2023) | Yes | Yes | Low | Yes | Low | Yes | Yes | Low | Yes | High | Moderate | Low | Low | Low |
| Pereira et al. (2024) | No | No | High | Yes | Low | Yes | Yes | Low | No | Low | Moderate | Low | Low | Low |
| Polzer et al. (2024) | Yes | No | High | Yes | Low | Yes | Yes | Low | No | Low | Moderate | High | High | Low |
| Potter et al. (2024) | Yes | No | High | Yes | Low | Yes | Yes | Low | No | Low | Moderate | High | High | Low |
| Roux et al. (2022) | Yes | Yes | Low | Yes | Low | Yes | Yes | Low | No | Low | Low | Low | Low | Low |
| Ruitenbeek et al. (2024) | Yes | Yes | Low | Yes | Low | No | No | High | No | Low | Moderate | Low | Low | Low |
| Seol et al. (2022) | Yes | N/A | Low | Yes | Low | No | Yes | High | No | Low | Moderate | High | High | Low |
| Shan et al. (2021) | Yes | N/A | Low | Yes | Low | No | Yes | High | No | Low | Moderate | Low | High | Low |
| Small et al. (2021) | Yes | Yes | Low | Yes | Low | Yes | No | High | No | Low | Moderate | Low | Low | Low |
| Tian et al. (2024) | Yes | No | High | Yes | Low | No | Yes | High | No | Low | Moderate | Low | High | Low |
| Tomita et al. (2018) | No | Yes | High | Yes | Low | No | Yes | High | No | Low | Moderate | High | High | Low |
| Tong et al. (2023) | Yes | No | High | Yes | Low | Yes | Yes | Low | No | Low | Moderate | High | High | Low |
| Ukai et al. (2023) | Yes | No | High | Yes | Low | No | Yes | High | No | Low | Moderate | High | High | Low |
| Van den Wittenboer et al. (2024) | Yes | No | High | Yes | Low | Yes | Yes | Low | No | Low | Moderate | Low | Low | Low |
| Voter et al. (2021) | Yes | Yes | Low | Yes | Low | No | No | High | No | Low | Moderate | Low | Low | Low |
| Weikert et al. (2020) | Yes | Yes | Low | Yes | Low | No | Yes | High | No | Low | Moderate | Low | High | Low |
| Wu et al. (2021) | Yes | No | High | Yes | Low | No | Yes | High | No | Low | Moderate | High | High | Low |
| H Wang et al. (2023) | Yes | No | High | Yes | Low | Yes | Yes | Low | No | Low | Moderate | Low | High | Low |
| S Wang et al. (2022) | Yes | No | High | Yes | Low | Yes | Yes | Low | No | Low | Moderate | Low | High | Low |
| X Wang et al. (2022) | Yes | No | High | Yes | Low | No | Yes | High | No | Low | Moderate | High | High | Low |
| Y Wang et al. (2024) | Yes | No | High | Yes | Low | Yes | Yes | Low | No | Low | Moderate | Low | High | Low |
| Yang et al. (2022) | Yes | Yes | Low | Yes | Low | Yes | Yes | Low | No | Low | Low | High | High | Low |
| Zhang et al. (2023) | Yes | No | High | Yes | Low | No | Yes | High | No | Low | Moderate | High | High | Low |
| Zhou et al. (2023) | Yes | Yes | Low | Yes | Low | Yes | Yes | Low | No | Low | Low | Low | Low | Low |

Abbreviations: SQ: Signalling Question; CS: Cohort Selection; IT: Index Test; RS: Reference Standard; F: Funding; TD: Test Dataset; CA: Commercially Availability; RF: Reference Standard

## Supplement 4. Heterogeneity measures

### Supplementary Heterogeneity for Figure 2. Diagnostic Accuracy with 95% Confidence Interval (CI) of Stand-Alone AI (Patient-Wise Level) by Unselected vs. Selected Cohort Representation

|  | T^2^ (Sensitivity) | I^2^ (Sensitivity) | T^2^ (Specificity) | I^2^ (Specificity) | T^2^ (Generalized) | I^2^ (Generalized) | Covariance | ρ |
| --- | --- | --- | --- | --- | --- | --- | --- | --- |
| A: Unselected | 0.62 | 0.89 | 0.87 | 0.90 | 0.49 | 0.89 | -0.23 | -0.31 |
| B: Selected | 1.35 | 0.91 | 0.85 | 0.88 | 1.00 | 0.89 | -0.37 | -0.35 |
| C: Not specified | 1.18 | 0.72 | 0.48 | 0.41 | 0.00 | 0.00 | 0.75 | 1.00 |
| Overall | 1.11 | 0.87 | 0.81 | 0.74 | 0.88 | 0.81 | -0.13 | -0.14 |

### Supplementary Heterogeneity for Figure 3. Diagnostic Accuracy with 95% Confidence Interval (CI) of Stand-Alone AI (Patient-Wise Level) by External vs. Internal Test Dataset Origin

|  | T^2^ (Sensitivity) | I^2^ (Sensitivity) | T^2^ (Specificity) | I^2^ (Specificity) | T^2^ (Generalized) | I^2^ (Generalized) | Covariance | ρ |
| --- | --- | --- | --- | --- | --- | --- | --- | --- |
| A: External Validation | 1.07 | 0.94 | 0.63 | 0.94 | 0.48 | 0.92 | -0.45 | -0.55 |
| B: Internal Valid. | 1.64 | 0.74 | 0.75 | 0.63 | 1.17 | 0.68 | 0.25 | 0.22 |
| C: Not specified | - | - | - | - | - | - | - | - |
| Overall | 1.15 | 0.87 | 0.78 | 0.73 | 0.89 | 0.81 | -0.05 | -0.06 |

### Supplementary Heterogeneity for Figure 4. Diagnostic Accuracy with 95% Confidence Interval (CI) of Stand-Alone AI on External Test Datasets by Level of Analysis

|  | T^2^ (Sensitivity) | I^2^ (Sensitivity) | T^2^ (Specificity) | I^2^ (Specificity) | T^2^ (Generalized) | I^2^ (Generalized) | Covariance | ρ |
| --- | --- | --- | --- | --- | --- | --- | --- | --- |
| A: Patient-Wise | 1.07 | 0.94 | 0.63 | 0.94 | 0.48 | 0.92 | -0.45 | -0.55 |
| B: Vertebra-Wise | 1.82 | 0.96 | 0.54 | 0.94 | 0.17 | 0.89 | -0.90 | -0.91 |
| C: Rib-Wise | 0.23 | 0.98 | 2.43 | 0.99 | 0.06 | 0.95 | 0.70 | 0.95 |
| Overall | 0.74 | 0.93 | 1.24 | 0.93 | 0.92 | 0.93 | 0.06 | 0.06 |

### Supplementary Heterogeneity for Figure 5. Diagnostic Accuracy with 95% Confidence Interval (CI) of Stand-Alone AI (Patient-Wise Level) by Commercially Available AI Fracture Detection Solution

|  | T^2^ (Sensitivity) | I^2^ (Sensitivity) | T^2^ (Specificity) | I^2^ (Specificity) | T^2^ (Generalized) | I^2^ (Generalized) | Covariance | ρ |
| --- | --- | --- | --- | --- | --- | --- | --- | --- |
| A: C-Spine | 0.45 | 0.92 | 0.32 | 0.91 | 0.14 | 0.92 | 0.01 | 0.01 |
| B: HealthOst Bone solution | - | - | - | - | - | - | - | - |
| C: HealthVCF | 0.59 | 0.92 | 0.58 | 0.97 | 0.00 | 0.00 | -0.59 | -1.00 |
| Overall | 0.46 | 0.91 | 0.89 | 0.97 | 0.31 | 0.94 | -0.32 | -0.50 |

### Supplementary Heterogeneity for Figure 6. Diagnostic Accuracy with 95% Confidence Interval (CI) of Stand-Alone AI (Patient-Wise Level) by Commercially Available AI Fracture Detection Solution vs. Non- Commercially Available AI Fracture Detection Solution

|  | T^2^ (Sensitivity) | I^2^ (Sensitivity) | T^2^ (Specificity) | I^2^ (Specificity) | T^2^ (Generalized) | I^2^ (Generalized) | Covariance | ρ |
| --- | --- | --- | --- | --- | --- | --- | --- | --- |
| CAAI-FDS | 0.46 | 0.91 | 0.89 | 0.97 | 0.31 | 0.94 | -0.32 | -0.50 |
| Non-CAAI-FDS} | 1.03 | 0.79 | 0.52 | 0.61 | 0.52 | 0.71 | 0.11 | 0.15 |
| Overall | 1.16 | 0.87 | 0.68 | 0.72 | 0.77 | 0.80 | -0.13 | -0.14 |

### Supplementary Heterogeneity for Figure 7. Diagnostic Accuracy with 95% Confidence Interval (CI) of Stand-Alone AI (Patient-Wise Level) by Anatomical Region

|  | T^2^ (Sensitivity) | I^2^ (Sensitivity) | T^2^ (Specificity) | I^2^ (Specificity) | T^2^ (Generalized) | I^2^ (Generalized) | Covariance | ρ |
| --- | --- | --- | --- | --- | --- | --- | --- | --- |
| A: Skull | 0.33 | 0.57 | 0.84 | 0.50 | 0.24 | 0.52 | 0.18 | 0.35 |
| B: Spine | 0.75 | 0.94 | 0.86 | 0.97 | 0.64 | 0.96 | -0.06 | -0.07 |
| C: Rib | 1.11 | 0.93 | 0.08 | 0.76 | 0.04 | 0.81 | -0.22 | -0.76 |
| D: Pelvis | - | - | - | - | - | - | - | - |
| Overall | 1.11 | 0.87 | 0.81 | 0.74 | 0.88 | 0.81 | -0.13 | -0.14 |

### Supplementary Heterogeneity for Figure 8. Diagnostic Accuracy with 95% Confidence Interval (CI) of Reader Type (Patient-Wise Level)

|  | T^2^ (Sensitivity) | I^2^ (Sensitivity) | T^2^ (Specificity) | I^2^ (Specificity) | T^2^ (Generalized) | I^2^ (Generalized) | Covariance | ρ |
| --- | --- | --- | --- | --- | --- | --- | --- | --- |
| A: Stand-Alone AI | 1.11 | 0.87 | 0.81 | 0.74 | 0.88 | 0.81 | -0.13 | -0.14 |
| B: Human Unaided | 0.87 | 0.94 | 1.51 | 0.88 | 1.25 | 0.92 | -0.23 | -0.20 |
| C: Human Aided | 0.09 | 0.55 | 0.53 | 0.69 | 0.00 | 0.00 | -0.22 | -1.00 |
| Overall | 0.85 | 0.85 | 0.75 | 0.71 | 0.63 | 0.79 | -0.07 | -0.09 |

## Supplement 5. Additional Analyses

### Supplementary Figure 1 with Heterogeneity Table. Diagnostic Accuracy with 95% Confidence Interval (CI) of Stand-Alone AI on Internal Test Datasets by Level of Analysis

|  | T^2^ (Sensitivity) | I^2^ (Sensitivity) | T^2^ (Specificity) | I^2^ (Specificity) | T^2^ (Generalized) | I^2^ (Generalized) | Covariance | ρ |
| --- | --- | --- | --- | --- | --- | --- | --- | --- |
| A: Sample-Wise | 1.24 | 0.68 | 1.48 | 0.54 | 0.00 | 0.00 | 1.35 | 1.00 |
| B: Patient-Wise | 1.64 | 0.74 | 0.75 | 0.63 | 1.17 | 0.68 | 0.25 | 0.22 |
| C: Vertebra-Wise | 0.52 | 0.65 | 1.14 | 0.88 | 0.09 | 0.59 | 0.71 | 0.92 |
| D: Rib-Wise | - | - | - | - | - | - | - | - |
| Overall | 1.26 | 0.73 | 1.05 | 0.64 | 1.06 | 0.66 | 0.51 | 0.44 |

### Supplementary Figure 2 with Heterogeneity Table. Diagnostic Accuracy with 95% Confidence Interval (CI) of Stand-Alone AI (Patient-Wise Level) by Reference Standard

|  | T^2^ (Sensitivity) | I^2^ (Sensitivity) | T^2^ (Specificity) | I^2^ (Specificity) | T^2^ (Generalized) | T^2^ (Generalized) | Covariance | ρ |
| --- | --- | --- | --- | --- | --- | --- | --- | --- |
| A: Expert Consensus | 1.24 | 0.89 | 0.98 | 0.68 | 1.04 | 0.80 | -0.42 | -0.38 |
| B: Others | 0.89 | 0.85 | 0.65 | 0.80 | 0.57 | 0.83 | 0.13 | 0.17 |
| Overall | 1.11 | 0.87 | 0.81 | 0.74 | 0.88 | 0.81 | -0.13 | -0.14 |

### Supplementary Figure 3 with Heterogeneity Table. Diagnostic Accuracy with 95% Confidence Interval (CI) of Stand-Alone AI (Patient-Wise Level) by Risk of Bias

|  | T^2^ (Sensitivity) | I^2^ (Sensitivity) | T^2^ (Specificity) | I^2^ (Specificity) | T^2^ (Generalized) | T^2^ (Generalized) | Covariance | ρ |
| --- | --- | --- | --- | --- | --- | --- | --- | --- |
| A: Low | 0.42 | 0.62 | 2.78 | 0.81 | 0.52 | 0.64 | 0.80 | 0.74 |
| B: Moderate | 1.10 | 0.86 | 0.76 | 0.71 | 0.80 | 0.79 | -0.20 | -0.22 |
| C: High | 0.13 | 0.81 | 0.03 | 0.73 | 0.00 | 0.00 | 0.07 | 1.00 |
| Overall | 1.11 | 0.87 | 0.81 | 0.74 | 0.88 | 0.81 | -0.13 | -0.14 |

### Supplementary Figure 4 with Heterogeneity Table. Diagnostic Accuracy with 95% Confidence Interval (CI) of Stand-Alone AI (Patient-Wise Level) by Funding

|  | T^2^ (Sensitivity) | I^2^ (Sensitivity) | T^2^ (Specificity) | I^2^ (Specificity) | T^2^ (Generalized) | T^2^ (Generalized) | Covariance | ρ |
| --- | --- | --- | --- | --- | --- | --- | --- | --- |
| A: Industrial Funding | 0.59 | 0.94 | 0.09 | 0.89 | 0.05 | 0.91 | 0.07 | 0.30 |
| B: No Funding | 1.14 | 0.85 | 0.99 | 0.73 | 1.10 | 0.79 | -0.19 | -0.18 |
| Overall | 1.11 | 0.87 | 0.81 | 0.74 | 0.88 | 0.81 | -0.13 | -0.14 |

### Supplementary Table 1. Study-Level Diagnostic Performance Metrics Underlying the Forest Plots in Figure 3 for Stand-Alone AI (Patient-Wise Level) by Test Dataset Origin

| **Author**  **(Year)** | **N** | **TP** | **FP** | **TN** | **FN** | **Prevalence [%]** | | **Sensitivity [%]** | | **Specificity [%]** | | **PPV [%]** | | **NPV [%]** | | **F1 score** |
| --- | --- | --- | --- | --- | --- | --- | --- | --- | --- | --- | --- | --- | --- | --- | --- | --- |
| Bendtsen et al. (2024) | 1000 | 65 | 81 | 824 | 30 | 9.5 | (7.8 - 11.5) | 68.4 | (58.5 - 76.9) | 91.0 | (89.0 - 92.7) | 44.5 | (36.7 - 52.6) | 96.5 | (95.0 - 97.5) | 0.54 |
| H Wang et al. (2023) | 192 | 86 | 5 | 90 | 11 | 50.5 | (43.5 - 57.5) | 88.7 | (80.8 - 93.5) | 94.7 | (88.3 - 97.7) | 94.5 | (87.8 - 97.6) | 89.1 | (81.5 - 93.8) | 0.91 |
| Kolanu et al. (2020) | 1570 | 183 | 97 | 1191 | 99 | 18.0 | (16.1 - 19.9) | 64.9 | (59.2 - 70.2) | 92.5 | (90.9 - 93.8) | 65.4 | (59.6 - 70.7) | 92.3 | (90.7 - 93.7) | 0.65 |
| Li et al. (2023) | 2319 | 2122 | 35 | 139 | 23 | 92.5 | (91.4 - 93.5) | 98.9 | (98.4 - 99.3) | 79.9 | (73.3 - 85.2) | 98.4 | (97.8 - 98.8) | 85.8 | (79.6 - 90.3) | 0.99 |
| Nicolaes et al. (2023a) | 4810 | 593 | 285 | 3897 | 35 | 13.1 | (12.1 - 14.0) | 94.4 | (92.3 - 96.0) | 93.2 | (92.4 - 93.9) | 67.5 | (64.4 - 70.6) | 99.1 | (98.8 - 99.4) | 0.79 |
| Nicolaes et al. (2023b) | 1943 | 240 | 91 | 1555 | 57 | 15.3 | (13.8 - 17.0) | 80.8 | (75.9 - 84.9) | 94.5 | (93.3 - 95.5) | 72.5 | (67.5 - 77.0) | 96.5 | (95.4 - 97.3) | 0.76 |
| Page et al. (2023) | 1087 | 107 | 124 | 826 | 30 | 12.6 | (10.8 - 14.7) | 78.1 | (70.5 - 84.2) | 86.9 | (84.7 - 88.9) | 46.3 | (40.0 - 52.8) | 96.5 | (95.0 - 97.5) | 0.58 |
| Pereira et al. (2024) | 899 | 107 | 55 | 699 | 38 | 16.1 | (13.9 - 18.7) | 73.8 | (66.1 - 80.3) | 92.7 | (90.6 - 94.4) | 66.0 | (58.5 - 72.9) | 94.8 | (93.0 - 96.2) | 0.70 |
| Roux et al. (2022) | 500 | 119 | 131 | 242 | 8 | 25.4 | (21.8 - 29.4) | 93.7 | (88.1 - 96.8) | 64.9 | (59.9 - 69.5) | 47.6 | (41.5 - 53.8) | 96.8 | (93.8 - 98.4) | 0.63 |
| Ruitenbeek et al. (2024) | 2036 | 150 | 88 | 1781 | 17 | 8.2 | (7.1 - 9.5) | 89.8 | (84.3 - 93.5) | 95.3 | (94.2 - 96.2) | 63.0 | (56.7 - 68.9) | 99.1 | (98.5 - 99.4) | 0.74 |
| S Wang et al. (2022) | 1613 | 909 | 90 | 547 | 67 | 60.5 | (58.1 - 62.9) | 93.1 | (91.4 - 94.6) | 85.9 | (83.0 - 88.4) | 91.0 | (89.1 - 92.6) | 89.1 | (86.4 - 91.3) | 0.92 |
| Shan et al. (2021) | 235 | 77 | 16 | 126 | 16 | 39.6 | (33.5 - 45.9) | 82.8 | (73.9 - 89.1) | 88.7 | (82.5 - 92.9) | 82.8 | (73.9 - 89.1) | 88.7 | (82.5 - 92.9) | 0.83 |
| Small et al. (2021) | 665 | 109 | 17 | 505 | 34 | 21.5 | (18.5 - 24.8) | 76.2 | (68.6 - 82.5) | 96.7 | (94.8 - 98.0) | 86.5 | (79.5 - 91.4) | 93.7 | (91.3 - 95.5) | 0.81 |
| Voter et al. (2021) | 1904 | 67 | 106 | 1676 | 55 | 6.4 | (5.4 - 7.6) | 54.9 | (46.1 - 63.5) | 94.1 | (92.9 - 95.1) | 38.7 | (31.8 - 46.2) | 96.8 | (95.9 - 97.6) | 0.45 |
| Weikert et al. (2020) | 510 | 139 | 30 | 321 | 20 | 31.2 | (27.3 - 35.3) | 87.4 | (81.4 - 91.7) | 91.5 | (88.1 - 93.9) | 82.2 | (75.8 - 87.3) | 94.1 | (91.1 - 96.2) | 0.85 |
| van den Wittenboer et al. (2024) | 2368 | 158 | 29 | 2118 | 63 | 9.3 | (8.2 - 10.6) | 71.5 | (65.2 - 77.0) | 98.6 | (98.1 - 99.1) | 84.5 | (78.6 - 89.0) | 97.1 | (96.3 - 97.7) | 0.77 |
| Amodeo et al. (2021) | 30 | 19 | 0 | 5 | 6 | 83.3 | (66.4 - 92.7) | 76.0 | (56.6 - 88.5) | 100.0 | (56.6 - 100.0) | 100.0 | (83.2 - 100.0) | 45.5 | (21.3 - 72.0) | 0.86 |
| Bao et al. (2023) | 302 | 195 | 1 | 101 | 5 | 66.2 | (60.7 - 71.3) | 97.5 | (94.3 - 98.9) | 99.0 | (94.7 - 99.8) | 99.5 | (97.2 - 99.9) | 95.3 | (89.4 - 98.0) | 0.98 |
| Castro-Zunti et al. (2024) | 2000 | 748 | 123 | 877 | 252 | 50.0 | (47.8 - 52.2) | 74.8 | (72.0 - 77.4) | 87.7 | (85.5 - 89.6) | 85.9 | (83.4 - 88.0) | 77.7 | (75.2 - 80.0) | 0.80 |
| Hu et al. (2021) | 252 | 80 | 36 | 128 | 8 | 34.9 | (29.3 - 41.0) | 90.9 | (83.1 - 95.3) | 78.0 | (71.1 - 83.7) | 69.0 | (60.1 - 76.7) | 94.1 | (88.8 - 97.0) | 0.78 |
| Jeong et al. (2024) | 50 | 24 | 4 | 22 | 0 | 48.0 | (34.8 - 61.5) | 100.0 | (86.2 - 100.0) | 84.6 | (66.5 - 93.8) | 85.7 | (68.5 - 94.3) | 100.0 | (85.1 - 100.0) | 0.92 |
| Li et al. (2023) | 1612 | 948 | 43 | 614 | 7 | 59.2 | (56.8 - 61.6) | 99.3 | (98.5 - 99.6) | 93.5 | (91.3 - 95.1) | 95.7 | (94.2 - 96.8) | 98.9 | (97.7 - 99.5) | 0.97 |
| Lu et al. (2024) | 841 | 148 | 9 | 662 | 22 | 20.2 | (17.6 - 23.1) | 87.1 | (81.2 - 91.3) | 98.7 | (97.5 - 99.3) | 94.3 | (89.5 - 97.0) | 96.8 | (95.2 - 97.9) | 0.91 |
| Moon et al. (2022) | 232 | 101 | 17 | 99 | 15 | 50.0 | (43.6 - 56.4) | 87.1 | (79.8 - 92.0) | 85.3 | (77.8 - 90.6) | 85.6 | (78.1 - 90.8) | 86.8 | (79.4 - 91.9) | 0.86 |
| Moon et al. (2024) | 40 | 21 | 3 | 16 | 0 | 52.5 | (37.5 - 67.1) | 100.0 | (84.5 - 100.0) | 84.2 | (62.4 - 94.5) | 87.5 | (69.0 - 95.7) | 100.0 | (80.6 - 100.0) | 0.93 |
| S Wang et al. (2022) | 1628 | 408 | 207 | 1001 | 12 | 25.8 | (23.7 - 28.0) | 97.1 | (95.1 - 98.4) | 82.9 | (80.6 - 84.9) | 66.3 | (62.5 - 70.0) | 98.8 | (97.9 - 99.3) | 0.79 |
| Seol et al. (2022) | 507 | 207 | 33 | 237 | 30 | 46.7 | (42.4 - 51.1) | 87.3 | (82.5 - 91.0) | 87.8 | (83.3 - 91.2) | 86.2 | (81.3 - 90.0) | 88.8 | (84.4 - 92.0) | 0.87 |
| Ukai et al. (2023) | 205 | 93 | 4 | 108 | 0 | 45.4 | (38.7 - 52.2) | 100.0 | (96.0 - 100.0) | 96.4 | (91.2 - 98.6) | 95.9 | (89.9 - 98.4) | 100.0 | (96.6 - 100.0) | 0.98 |
| Wu et al. (2021) | 8051 | 275 | 1138 | 6600 | 38 | 3.9 | (3.5 - 4.3) | 87.9 | (83.8 - 91.0) | 85.3 | (84.5 - 86.1) | 19.5 | (17.5 - 21.6) | 99.4 | (99.2 - 99.6) | 0.32 |
| Yang et al. (2022) | 76 | 39 | 4 | 26 | 7 | 60.5 | (49.3 - 70.8) | 84.8 | (71.8 - 92.4) | 86.7 | (70.3 - 94.7) | 90.7 | (78.4 - 96.3) | 78.8 | (62.2 - 89.3) | 0.88 |
| Nadeem et al. (2024) | 3231 | 1007 | 33 | 2136 | 55 | 32.9 | (31.3 - 34.5) | 94.8 | (93.3 - 96.0) | 98.5 | (97.9 - 98.9) | 96.8 | (95.6 - 97.7) | 97.5 | (96.7 - 98.1) | 0.96 |

Abbreviations: NPV = Negative Predictive Value; PPV = Positive Predictive Value; TP = True Positive; FP = False Positive; TN = True Negative; FN = False Negative

### Supplementary Table 2. Study-Level Diagnostic Performance Metrics Underlying the Forest Plots in Figure 4 for Stand-Alone AI on External Test Datasets by Level of Analysis

| **Author**  **(Year)** | **N** | **TP** | **FP** | **TN** | **FN** | **Prevalence [%]** | | **Sensitivity [%]** | | **Specificity [%]** | | **PPV [%]** | | **NPV [%]** | | **F1 score** |
| --- | --- | --- | --- | --- | --- | --- | --- | --- | --- | --- | --- | --- | --- | --- | --- | --- |
| Bendtsen et al. (2024) | 1000 | 65 | 81 | 824 | 30 | 9.5 | (7.8 - 11.5) | 68.4 | (58.5 - 76.9) | 91.0 | (89.0 - 92.7) | 44.5 | (36.7 - 52.6) | 96.5 | (95.0 - 97.5) | 0.54 |
| H Wang et al. (2023) | 192 | 86 | 5 | 90 | 11 | 50.5 | (43.5 - 57.5) | 88.7 | (80.8 - 93.5) | 94.7 | (88.3 - 97.7) | 94.5 | (87.8 - 97.6) | 89.1 | (81.5 - 93.8) | 0.91 |
| Kolanu et al. (2020) | 1570 | 183 | 97 | 1191 | 99 | 18.0 | (16.1 - 19.9) | 64.9 | (59.2 - 70.2) | 92.5 | (90.9 - 93.8) | 65.4 | (59.6 - 70.7) | 92.3 | (90.7 - 93.7) | 0.65 |
| Li et al. (2023) | 2319 | 2122 | 35 | 139 | 23 | 92.5 | (91.4 - 93.5) | 98.9 | (98.4 - 99.3) | 79.9 | (73.3 - 85.2) | 98.4 | (97.8 - 98.8) | 85.8 | (79.6 - 90.3) | 0.99 |
| Nicolaes et al. (2023b) | 1943 | 240 | 91 | 1555 | 57 | 15.3 | (13.8 - 17.0) | 80.8 | (75.9 - 84.9) | 94.5 | (93.3 - 95.5) | 72.5 | (67.5 - 77.0) | 96.5 | (95.4 - 97.3) | 0.76 |
| Nicolaes et al. (2023a) | 4810 | 593 | 285 | 3897 | 35 | 13.1 | (12.1 - 14.0) | 94.4 | (92.3 - 96.0) | 93.2 | (92.4 - 93.9) | 67.5 | (64.4 - 70.6) | 99.1 | (98.8 - 99.4) | 0.79 |
| Page et al. (2023) | 1087 | 107 | 124 | 826 | 30 | 12.6 | (10.8 - 14.7) | 78.1 | (70.5 - 84.2) | 86.9 | (84.7 - 88.9) | 46.3 | (40.0 - 52.8) | 96.5 | (95.0 - 97.5) | 0.58 |
| Pereira et al. (2024) | 899 | 107 | 55 | 699 | 38 | 16.1 | (13.9 - 18.7) | 73.8 | (66.1 - 80.3) | 92.7 | (90.6 - 94.4) | 66.0 | (58.5 - 72.9) | 94.8 | (93.0 - 96.2) | 0.70 |
| Roux et al. (2022) | 500 | 119 | 131 | 242 | 8 | 25.4 | (21.8 - 29.4) | 93.7 | (88.1 - 96.8) | 64.9 | (59.9 - 69.5) | 47.6 | (41.5 - 53.8) | 96.8 | (93.8 - 98.4) | 0.63 |
| Ruitenbeek et al. (2024) | 2036 | 150 | 88 | 1781 | 17 | 8.2 | (7.1 - 9.5) | 89.8 | (84.3 - 93.5) | 95.3 | (94.2 - 96.2) | 63.0 | (56.7 - 68.9) | 99.1 | (98.5 - 99.4) | 0.74 |
| S Wang et al. (2022) | 1613 | 909 | 90 | 547 | 67 | 60.5 | (58.1 - 62.9) | 93.1 | (91.4 - 94.6) | 85.9 | (83.0 - 88.4) | 91.0 | (89.1 - 92.6) | 89.1 | (86.4 - 91.3) | 0.92 |
| Shan et al. (2021) | 235 | 77 | 16 | 126 | 16 | 39.6 | (33.5 - 45.9) | 82.8 | (73.9 - 89.1) | 88.7 | (82.5 - 92.9) | 82.8 | (73.9 - 89.1) | 88.7 | (82.5 - 92.9) | 0.83 |
| Small et al. (2021) | 665 | 109 | 17 | 505 | 34 | 21.5 | (18.5 - 24.8) | 76.2 | (68.6 - 82.5) | 96.7 | (94.8 - 98.0) | 86.5 | (79.5 - 91.4) | 93.7 | (91.3 - 95.5) | 0.81 |
| Voter et al. (2021) | 1904 | 67 | 106 | 1676 | 55 | 6.4 | (5.4 - 7.6) | 54.9 | (46.1 - 63.5) | 94.1 | (92.9 - 95.1) | 38.7 | (31.8 - 46.2) | 96.8 | (95.9 - 97.6) | 0.45 |
| Weikert et al. (2020) | 510 | 139 | 30 | 321 | 20 | 31.2 | (27.3 - 35.3) | 87.4 | (81.4 - 91.7) | 91.5 | (88.1 - 93.9) | 82.2 | (75.8 - 87.3) | 94.1 | (91.1 - 96.2) | 0.85 |
| van den Wittenboer et al. (2024) | 2368 | 158 | 29 | 2118 | 63 | 9.3 | (8.2 - 10.6) | 71.5 | (65.2 - 77.0) | 98.6 | (98.1 - 99.1) | 84.5 | (78.6 - 89.0) | 97.1 | (96.3 - 97.7) | 0.77 |
| Nicolaes et al. (2023a) | 24930 | 353 | 176 | 24091 | 310 | 2.7 | (2.5 - 2.9) | 53.2 | (49.4 - 57.0) | 99.3 | (99.2 - 99.4) | 66.7 | (62.6 - 70.6) | 98.7 | (98.6 - 98.9) | 0.59 |
| Nicolaes et al. (2023b) | 48584 | 786 | 458 | 47227 | 113 | 1.9 | (1.7 - 2.0) | 87.4 | (85.1 - 89.4) | 99.0 | (98.9 - 99.1) | 63.2 | (60.5 - 65.8) | 99.8 | (99.7 - 99.8) | 0.73 |
| Tian et al. (2024) | 887 | 119 | 22 | 734 | 12 | 14.8 | (12.6 - 17.3) | 90.8 | (84.7 - 94.7) | 97.1 | (95.6 - 98.1) | 84.4 | (77.5 - 89.5) | 98.4 | (97.2 - 99.1) | 0.88 |
| Y Wang et al. (2024) | 2823 | 390 | 104 | 2321 | 8 | 14.1 | (12.9 - 15.4) | 98.0 | (96.1 - 99.0) | 95.7 | (94.8 - 96.4) | 78.9 | (75.1 - 82.3) | 99.7 | (99.3 - 99.8) | 0.87 |
| Li et al. (2023) | 55250 | 9508 | 197 | 44761 | 784 | 18.6 | (18.3 - 19.0) | 92.4 | (91.9 - 92.9) | 99.6 | (99.5 - 99.6) | 98.0 | (97.7 - 98.2) | 98.3 | (98.2 - 98.4) | 0.95 |
| S Wang et al. (2022) | 38712 | 2941 | 424 | 34876 | 471 | 8.8 | (8.5 - 9.1) | 86.2 | (85.0 - 87.3) | 98.8 | (98.7 - 98.9) | 87.4 | (86.2 - 88.5) | 98.7 | (98.5 - 98.8) | 0.87 |
| Zhou et al. (2023) | 2712 | 565 | 302 | 1698 | 147 | 26.3 | (24.6 - 27.9) | 79.4 | (76.2 - 82.2) | 84.9 | (83.3 - 86.4) | 65.2 | (61.9 - 68.3) | 92.0 | (90.7 - 93.2) | 0.72 |

Abbreviations: NPV = Negative Predictive Value; PPV = Positive Predictive Value; TP = True Positive; FP = False Positive; TN = True Negative; FN = False Negative

### Supplementary Table 3. Study-Level Diagnostic Performance Metrics Underlying the Forest Plots in Figure 6 for Stand-Alone AI (Patient-Wise Level) by Commercial Availability of the AI Fracture Detection Solution

| **Author**  **(Year)** | **N** | **TP** | **FP** | **TN** | **FN** | **Prevalence [%]** | | **Sensitivity [%]** | | **Specificity [%]** | | **PPV [%]** | | **NPV [%]** | | **F1 score** |
| --- | --- | --- | --- | --- | --- | --- | --- | --- | --- | --- | --- | --- | --- | --- | --- | --- |
| Bendtsen et al. (2024) | 1000 | 65 | 81 | 824 | 30 | 9.5 | (7.8 - 11.5) | 68.4 | (58.5 - 76.9) | 91.0 | (89.0 - 92.7) | 44.5 | (36.7 - 52.6) | 96.5 | (95.0 - 97.5) | 0.54 |
| Kolanu et al. (2020) | 1570 | 183 | 97 | 1191 | 99 | 18.0 | (16.1 - 19.9) | 64.9 | (59.2 - 70.2) | 92.5 | (90.9 - 93.8) | 65.4 | (59.6 - 70.7) | 92.3 | (90.7 - 93.7) | 0.65 |
| Page et al. (2023) | 1087 | 107 | 124 | 826 | 30 | 12.6 | (10.8 - 14.7) | 78.1 | (70.5 - 84.2) | 86.9 | (84.7 - 88.9) | 46.3 | (40.0 - 52.8) | 96.5 | (95.0 - 97.5) | 0.58 |
| Pereira et al. (2024) | 899 | 107 | 55 | 699 | 38 | 16.1 | (13.9 - 18.7) | 73.8 | (66.1 - 80.3) | 92.7 | (90.6 - 94.4) | 66.0 | (58.5 - 72.9) | 94.8 | (93.0 - 96.2) | 0.70 |
| Roux et al. (2022) | 500 | 119 | 131 | 242 | 8 | 25.4 | (21.8 - 29.4) | 93.7 | (88.1 - 96.8) | 64.9 | (59.9 - 69.5) | 47.6 | (41.5 - 53.8) | 96.8 | (93.8 - 98.4) | 0.63 |
| Ruitenbeek et al. (2024) | 2036 | 150 | 88 | 1781 | 17 | 8.2 | (7.1 - 9.5) | 89.8 | (84.3 - 93.5) | 95.3 | (94.2 - 96.2) | 63.0 | (56.7 - 68.9) | 99.1 | (98.5 - 99.4) | 0.74 |
| Small et al. (2021) | 665 | 109 | 17 | 505 | 34 | 21.5 | (18.5 - 24.8) | 76.2 | (68.6 - 82.5) | 96.7 | (94.8 - 98.0) | 86.5 | (79.5 - 91.4) | 93.7 | (91.3 - 95.5) | 0.81 |
| Voter et al. (2021) | 1904 | 67 | 106 | 1676 | 55 | 6.4 | (5.4 - 7.6) | 54.9 | (46.1 - 63.5) | 94.1 | (92.9 - 95.1) | 38.7 | (31.8 - 46.2) | 96.8 | (95.9 - 97.6) | 0.45 |
| van den Wittenboer et al. (2024) | 2368 | 158 | 29 | 2118 | 63 | 9.3 | (8.2 - 10.6) | 71.5 | (65.2 - 77.0) | 98.6 | (98.1 - 99.1) | 84.5 | (78.6 - 89.0) | 97.1 | (96.3 - 97.7) | 0.77 |
| Amodeo et al. (2021) | 30 | 19 | 0 | 5 | 6 | 83.3 | (66.4 - 92.7) | 76.0 | (56.6 - 88.5) | 100.0 | (56.6 - 100.0) | 100.0 | (83.2 - 100.0) | 45.5 | (21.3 - 72.0) | 0.86 |
| Bao et al. (2023) | 302 | 195 | 1 | 101 | 5 | 66.2 | (60.7 - 71.3) | 97.5 | (94.3 - 98.9) | 99.0 | (94.7 - 99.8) | 99.5 | (97.2 - 99.9) | 95.3 | (89.4 - 98.0) | 0.98 |
| Castro-Zunti et al. (2024) | 2000 | 748 | 123 | 877 | 252 | 50.0 | (47.8 - 52.2) | 74.8 | (72.0 - 77.4) | 87.7 | (85.5 - 89.6) | 85.9 | (83.4 - 88.0) | 77.7 | (75.2 - 80.0) | 0.80 |
| H Wang et al. (2023) | 192 | 86 | 5 | 90 | 11 | 50.5 | (43.5 - 57.5) | 88.7 | (80.8 - 93.5) | 94.7 | (88.3 - 97.7) | 94.5 | (87.8 - 97.6) | 89.1 | (81.5 - 93.8) | 0.91 |
| Hu et al. (2021) | 252 | 80 | 36 | 128 | 8 | 34.9 | (29.3 - 41.0) | 90.9 | (83.1 - 95.3) | 78.0 | (71.1 - 83.7) | 69.0 | (60.1 - 76.7) | 94.1 | (88.8 - 97.0) | 0.78 |
| Jeong et al. (2024) | 50 | 24 | 4 | 22 | 0 | 48.0 | (34.8 - 61.5) | 100.0 | (86.2 - 100.0) | 84.6 | (66.5 - 93.8) | 85.7 | (68.5 - 94.3) | 100.0 | (85.1 - 100.0) | 0.92 |
| Li et al. (2023) | 1612 | 948 | 43 | 614 | 7 | 59.2 | (56.8 - 61.6) | 99.3 | (98.5 - 99.6) | 93.5 | (91.3 - 95.1) | 95.7 | (94.2 - 96.8) | 98.9 | (97.7 - 99.5) | 0.97 |
| Li et al. (2023) | 2319 | 2122 | 35 | 139 | 23 | 92.5 | (91.4 - 93.5) | 98.9 | (98.4 - 99.3) | 79.9 | (73.3 - 85.2) | 98.4 | (97.8 - 98.8) | 85.8 | (79.6 - 90.3) | 0.99 |
| Lu et al. (2024) | 841 | 148 | 9 | 662 | 22 | 20.2 | (17.6 - 23.1) | 87.1 | (81.2 - 91.3) | 98.7 | (97.5 - 99.3) | 94.3 | (89.5 - 97.0) | 96.8 | (95.2 - 97.9) | 0.91 |
| Moon et al. (2024) | 232 | 101 | 17 | 99 | 15 | 50.0 | (43.6 - 56.4) | 87.1 | (79.8 - 92.0) | 85.3 | (77.8 - 90.6) | 85.6 | (78.1 - 90.8) | 86.8 | (79.4 - 91.9) | 0.86 |
| Moon et al. (2022) | 40 | 21 | 3 | 16 | 0 | 52.5 | (37.5 - 67.1) | 100.0 | (84.5 - 100.0) | 84.2 | (62.4 - 94.5) | 87.5 | (69.0 - 95.7) | 100.0 | (80.6 - 100.0) | 0.93 |
| Nicolaes et al. (2023a) | 4810 | 593 | 285 | 3897 | 35 | 13.1 | (12.1 - 14.0) | 94.4 | (92.3 - 96.0) | 93.2 | (92.4 - 93.9) | 67.5 | (64.4 - 70.6) | 99.1 | (98.8 - 99.4) | 0.79 |
| Nicolaes et al. (2023b) | 1943 | 240 | 91 | 1555 | 57 | 15.3 | (13.8 - 17.0) | 80.8 | (75.9 - 84.9) | 94.5 | (93.3 - 95.5) | 72.5 | (67.5 - 77.0) | 96.5 | (95.4 - 97.3) | 0.76 |
| S Wang et al. (2022) | 1628 | 408 | 207 | 1001 | 12 | 25.8 | (23.7 - 28.0) | 97.1 | (95.1 - 98.4) | 82.9 | (80.6 - 84.9) | 66.3 | (62.5 - 70.0) | 98.8 | (97.9 - 99.3) | 0.79 |
| S Wang et al. (2022) | 1613 | 909 | 90 | 547 | 67 | 60.5 | (58.1 - 62.9) | 93.1 | (91.4 - 94.6) | 85.9 | (83.0 - 88.4) | 91.0 | (89.1 - 92.6) | 89.1 | (86.4 - 91.3) | 0.92 |
| Seol et al. (2022) | 507 | 207 | 33 | 237 | 30 | 46.7 | (42.4 - 51.1) | 87.3 | (82.5 - 91.0) | 87.8 | (83.3 - 91.2) | 86.2 | (81.3 - 90.0) | 88.8 | (84.4 - 92.0) | 0.87 |
| Shan et al. (2021) | 235 | 77 | 16 | 126 | 16 | 39.6 | (33.5 - 45.9) | 82.8 | (73.9 - 89.1) | 88.7 | (82.5 - 92.9) | 82.8 | (73.9 - 89.1) | 88.7 | (82.5 - 92.9) | 0.83 |
| Ukai et al. (2023) | 205 | 93 | 4 | 108 | 0 | 45.4 | (38.7 - 52.2) | 100.0 | (96.0 - 100.0) | 96.4 | (91.2 - 98.6) | 95.9 | (89.9 - 98.4) | 100.0 | (96.6 - 100.0) | 0.98 |
| Weikert et al. (2020) | 510 | 139 | 30 | 321 | 20 | 31.2 | (27.3 - 35.3) | 87.4 | (81.4 - 91.7) | 91.5 | (88.1 - 93.9) | 82.2 | (75.8 - 87.3) | 94.1 | (91.1 - 96.2) | 0.85 |
| Wu et al. (2021) | 8051 | 275 | 1138 | 6600 | 38 | 3.9 | (3.5 - 4.3) | 87.9 | (83.8 - 91.0) | 85.3 | (84.5 - 86.1) | 19.5 | (17.5 - 21.6) | 99.4 | (99.2 - 99.6) | 0.32 |
| Yang et al. (2022) | 76 | 39 | 4 | 26 | 7 | 60.5 | (49.3 - 70.8) | 84.8 | (71.8 - 92.4) | 86.7 | (70.3 - 94.7) | 90.7 | (78.4 - 96.3) | 78.8 | (62.2 - 89.3) | 0.88 |

Abbreviations: NPV = Negative Predictive Value; PPV = Positive Predictive Value; TP = True Positive; FP = False Positive; TN = True Negative; FN = False Negative

### **Supplementary Table 4.** Study-Level Diagnostic Performance Metrics Underlying the Forest Plots in Figure 7 for Stand-Alone AI (Patient-Wise Level) by Anatomical Region

| **Author**  **(Year)** | **N** | **TP** | **FP** | **TN** | **FN** | **Prevalence [%]** | | **Sensitivity [%]** | | **Specificity [%]** | | **PPV [%]** | | **NPV [%]** | | **F1 score** |
| --- | --- | --- | --- | --- | --- | --- | --- | --- | --- | --- | --- | --- | --- | --- | --- | --- |
| Amodeo et al. (2021) | 30 | 19 | 0 | 5 | 6 | 83.3 | (66.4 - 92.7) | 76.0 | (56.6 - 88.5) | 100.0 | (56.6 - 100.0) | 100.0 | (83.2 - 100.0) | 45.5 | (21.3 - 72.0) | 0.86 |
| Bao et al. (2023) | 302 | 195 | 1 | 101 | 5 | 66.2 | (60.7 - 71.3) | 97.5 | (94.3 - 98.9) | 99.0 | (94.7 - 99.8) | 99.5 | (97.2 - 99.9) | 95.3 | (89.4 - 98.0) | 0.98 |
| H Wang et al. (2023) | 192 | 86 | 5 | 90 | 11 | 50.5 | (43.5 - 57.5) | 88.7 | (80.8 - 93.5) | 94.7 | (88.3 - 97.7) | 94.5 | (87.8 - 97.6) | 89.1 | (81.5 - 93.8) | 0.91 |
| Jeong et al. (2024) | 50 | 24 | 4 | 22 | 0 | 48.0 | (34.8 - 61.5) | 100.0 | (86.2 - 100.0) | 84.6 | (66.5 - 93.8) | 85.7 | (68.5 - 94.3) | 100.0 | (85.1 - 100.0) | 0.92 |
| Lu et al. (2024) | 841 | 148 | 9 | 662 | 22 | 20.2 | (17.6 - 23.1) | 87.1 | (81.2 - 91.3) | 98.7 | (97.5 - 99.3) | 94.3 | (89.5 - 97.0) | 96.8 | (95.2 - 97.9) | 0.91 |
| Moon et al. (2022) | 40 | 21 | 3 | 16 | 0 | 52.5 | (37.5 - 67.1) | 100.0 | (84.5 - 100.0) | 84.2 | (62.4 - 94.5) | 87.5 | (69.0 - 95.7) | 100.0 | (80.6 - 100.0) | 0.93 |
| Moon et al. (2024) | 232 | 101 | 17 | 99 | 15 | 50.0 | (43.6 - 56.4) | 87.1 | (79.8 - 92.0) | 85.3 | (77.8 - 90.6) | 85.6 | (78.1 - 90.8) | 86.8 | (79.4 - 91.9) | 0.86 |
| Seol et al. (2022) | 507 | 207 | 33 | 237 | 30 | 46.7 | (42.4 - 51.1) | 87.3 | (82.5 - 91.0) | 87.8 | (83.3 - 91.2) | 86.2 | (81.3 - 90.0) | 88.8 | (84.4 - 92.0) | 0.87 |
| Shan et al. (2021) | 235 | 77 | 16 | 126 | 16 | 39.6 | (33.5 - 45.9) | 82.8 | (73.9 - 89.1) | 88.7 | (82.5 - 92.9) | 82.8 | (73.9 - 89.1) | 88.7 | (82.5 - 92.9) | 0.83 |
| Yang et al. (2022) | 76 | 39 | 4 | 26 | 7 | 60.5 | (49.3 - 70.8) | 84.8 | (71.8 - 92.4) | 86.7 | (70.3 - 94.7) | 90.7 | (78.4 - 96.3) | 78.8 | (62.2 - 89.3) | 0.88 |
| Bendtsen et al. (2024) | 1000 | 65 | 81 | 824 | 30 | 9.5 | (7.8 - 11.5) | 68.4 | (58.5 - 76.9) | 91.0 | (89.0 - 92.7) | 44.5 | (36.7 - 52.6) | 96.5 | (95.0 - 97.5) | 0.54 |
| Kolanu et al. (2020) | 1570 | 183 | 97 | 1191 | 99 | 18.0 | (16.1 - 19.9) | 64.9 | (59.2 - 70.2) | 92.5 | (90.9 - 93.8) | 65.4 | (59.6 - 70.7) | 92.3 | (90.7 - 93.7) | 0.65 |
| Nadeem et al. (2024) | 3231 | 1007 | 33 | 2136 | 55 | 32.9 | (31.3 - 34.5) | 94.8 | (93.3 - 96.0) | 98.5 | (97.9 - 98.9) | 96.8 | (95.6 - 97.7) | 97.5 | (96.7 - 98.1) | 0.96 |
| Nicolaes et al. (2023b) | 4810 | 593 | 285 | 3897 | 35 | 13.1 | (12.1 - 14.0) | 94.4 | (92.3 - 96.0) | 93.2 | (92.4 - 93.9) | 67.5 | (64.4 - 70.6) | 99.1 | (98.8 - 99.4) | 0.79 |
| Nicolaes et al. (2023a) | 1943 | 240 | 91 | 1555 | 57 | 15.3 | (13.8 - 17.0) | 80.8 | (75.9 - 84.9) | 94.5 | (93.3 - 95.5) | 72.5 | (67.5 - 77.0) | 96.5 | (95.4 - 97.3) | 0.76 |
| Page et al. (2023) | 1087 | 107 | 124 | 826 | 30 | 12.6 | (10.8 - 14.7) | 78.1 | (70.5 - 84.2) | 86.9 | (84.7 - 88.9) | 46.3 | (40.0 - 52.8) | 96.5 | (95.0 - 97.5) | 0.58 |
| Pereira et al. (2024) | 899 | 107 | 55 | 699 | 38 | 16.1 | (13.9 - 18.7) | 73.8 | (66.1 - 80.3) | 92.7 | (90.6 - 94.4) | 66.0 | (58.5 - 72.9) | 94.8 | (93.0 - 96.2) | 0.70 |
| Roux et al. (2022) | 500 | 119 | 131 | 242 | 8 | 25.4 | (21.8 - 29.4) | 93.7 | (88.1 - 96.8) | 64.9 | (59.9 - 69.5) | 47.6 | (41.5 - 53.8) | 96.8 | (93.8 - 98.4) | 0.63 |
| Ruitenbeek et al. (2024) | 2036 | 150 | 88 | 1781 | 17 | 8.2 | (7.1 - 9.5) | 89.8 | (84.3 - 93.5) | 95.3 | (94.2 - 96.2) | 63.0 | (56.7 - 68.9) | 99.1 | (98.5 - 99.4) | 0.74 |
| Small et al. (2021) | 665 | 109 | 17 | 505 | 34 | 21.5 | (18.5 - 24.8) | 76.2 | (68.6 - 82.5) | 96.7 | (94.8 - 98.0) | 86.5 | (79.5 - 91.4) | 93.7 | (91.3 - 95.5) | 0.81 |
| Voter et al. (2021) | 1904 | 67 | 106 | 1676 | 55 | 6.4 | (5.4 - 7.6) | 54.9 | (46.1 - 63.5) | 94.1 | (92.9 - 95.1) | 38.7 | (31.8 - 46.2) | 96.8 | (95.9 - 97.6) | 0.45 |
| van den Wittenboer et al. (2024) | 2368 | 158 | 29 | 2118 | 63 | 9.3 | (8.2 - 10.6) | 71.5 | (65.2 - 77.0) | 98.6 | (98.1 - 99.1) | 84.5 | (78.6 - 89.0) | 97.1 | (96.3 - 97.7) | 0.77 |
| Castro-Zunti et al. (2024) | 2000 | 748 | 123 | 877 | 252 | 50.0 | (47.8 - 52.2) | 74.8 | (72.0 - 77.4) | 87.7 | (85.5 - 89.6) | 85.9 | (83.4 - 88.0) | 77.7 | (75.2 - 80.0) | 0.80 |
| Hu et al. (2021) | 252 | 80 | 36 | 128 | 8 | 34.9 | (29.3 - 41.0) | 90.9 | (83.1 - 95.3) | 78.0 | (71.1 - 83.7) | 69.0 | (60.1 - 76.7) | 94.1 | (88.8 - 97.0) | 0.78 |
| Li et al. (2023) | 2319 | 2122 | 35 | 139 | 23 | 92.5 | (91.4 - 93.5) | 98.9 | (98.4 - 99.3) | 79.9 | (73.3 - 85.2) | 98.4 | (97.8 - 98.8) | 85.8 | (79.6 - 90.3) | 0.99 |
| S Wang et al. (2022) | 1613 | 909 | 90 | 547 | 67 | 60.5 | (58.1 - 62.9) | 93.1 | (91.4 - 94.6) | 85.9 | (83.0 - 88.4) | 91.0 | (89.1 - 92.6) | 89.1 | (86.4 - 91.3) | 0.92 |
| Weikert et al. (2020) | 510 | 139 | 30 | 321 | 20 | 31.2 | (27.3 - 35.3) | 87.4 | (81.4 - 91.7) | 91.5 | (88.1 - 93.9) | 82.2 | (75.8 - 87.3) | 94.1 | (91.1 - 96.2) | 0.85 |
| Wu et al. (2021) | 8051 | 275 | 1138 | 6600 | 38 | 3.9 | (3.5 - 4.3) | 87.9 | (83.8 - 91.0) | 85.3 | (84.5 - 86.1) | 19.5 | (17.5 - 21.6) | 99.4 | (99.2 - 99.6) | 0.32 |
| Ukai et al. (2023) | 205 | 93 | 4 | 108 | 0 | 45.4 | (38.7 - 52.2) | 100.0 | (96.0 - 100.0) | 96.4 | (91.2 - 98.6) | 95.9 | (89.9 - 98.4) | 100.0 | (96.6 - 100.0) | 0.98 |

Abbreviations: NPV = Negative Predictive Value; PPV = Positive Predictive Value; TP = True Positive; FP = False Positive; TN = True Negative; FN = False Negative

### Supplementary Table 5. Study-Level Diagnostic Performance Metrics Underlying the Forest Plots in Figure 8 by Reader Type (Patient-Wise Level)

| **Author**  **(Year)** | **N** | **TP** | **FP** | **TN** | **FN** | **Prevalence [%]** | | **Sensitivity [%]** | | **Specificity [%]** | | **PPV [%]** | | **NPV [%]** | | **F1 score** | |
| --- | --- | --- | --- | --- | --- | --- | --- | --- | --- | --- | --- | --- | --- | --- | --- | --- | --- |
| Amodeo et al. (2021) | 30 | 19 | 0 | 5 | 6 | 83.3 | (66.4 - 92.7) | 76.0 | (56.6 - 88.5) | 100.0 | (56.6 - 100.0) | 100.0 | (83.2 - 100.0) | 45.5 | (21.3 - 72.0) | | 0.86 |
| Bao et al. (2023) | 302 | 195 | 1 | 101 | 5 | 66.2 | (60.7 - 71.3) | 97.5 | (94.3 - 98.9) | 99.0 | (94.7 - 99.8) | 99.5 | (97.2 - 99.9) | 95.3 | (89.4 - 98.0) | | 0.98 |
| Bendtsen et al. (2024) | 1000 | 65 | 81 | 824 | 30 | 9.5 | (7.8 - 11.5) | 68.4 | (58.5 - 76.9) | 91.0 | (89.0 - 92.7) | 44.5 | (36.7 - 52.6) | 96.5 | (95.0 - 97.5) | | 0.54 |
| Castro-Zunti et al. (2024) | 2000 | 748 | 123 | 877 | 252 | 50.0 | (47.8 - 52.2) | 74.8 | (72.0 - 77.4) | 87.7 | (85.5 - 89.6) | 85.9 | (83.4 - 88.0) | 77.7 | (75.2 - 80.0) | | 0.80 |
| H Wang et al. (2023) | 192 | 86 | 5 | 90 | 11 | 50.5 | (43.5 - 57.5) | 88.7 | (80.8 - 93.5) | 94.7 | (88.3 - 97.7) | 94.5 | (87.8 - 97.6) | 89.1 | (81.5 - 93.8) | | 0.91 |
| Hu et al. (2021) | 252 | 80 | 36 | 128 | 8 | 34.9 | (29.3 - 41.0) | 90.9 | (83.1 - 95.3) | 78.0 | (71.1 - 83.7) | 69.0 | (60.1 - 76.7) | 94.1 | (88.8 - 97.0) | | 0.78 |
| Jeong et al. (2024) | 50 | 24 | 4 | 22 | 0 | 48.0 | (34.8 - 61.5) | 100.0 | (86.2 - 100.0) | 84.6 | (66.5 - 93.8) | 85.7 | (68.5 - 94.3) | 100.0 | (85.1 - 100.0) | | 0.92 |
| Kolanu et al. (2020) | 1570 | 183 | 97 | 1191 | 99 | 18.0 | (16.1 - 19.9) | 64.9 | (59.2 - 70.2) | 92.5 | (90.9 - 93.8) | 65.4 | (59.6 - 70.7) | 92.3 | (90.7 - 93.7) | | 0.65 |
| Li et al. (2023) | 2319 | 2122 | 35 | 139 | 23 | 92.5 | (91.4 - 93.5) | 98.9 | (98.4 - 99.3) | 79.9 | (73.3 - 85.2) | 98.4 | (97.8 - 98.8) | 85.8 | (79.6 - 90.3) | | 0.99 |
| Lu et al. (2024) | 841 | 148 | 9 | 662 | 22 | 20.2 | (17.6 - 23.1) | 87.1 | (81.2 - 91.3) | 98.7 | (97.5 - 99.3) | 94.3 | (89.5 - 97.0) | 96.8 | (95.2 - 97.9) | | 0.91 |
| Moon et al. (2024) | 232 | 101 | 17 | 99 | 15 | 50.0 | (43.6 - 56.4) | 87.1 | (79.8 - 92.0) | 85.3 | (77.8 - 90.6) | 85.6 | (78.1 - 90.8) | 86.8 | (79.4 - 91.9) | | 0.86 |
| Moon et al. (2022) | 40 | 21 | 3 | 16 | 0 | 52.5 | (37.5 - 67.1) | 100.0 | (84.5 - 100.0) | 84.2 | (62.4 - 94.5) | 87.5 | (69.0 - 95.7) | 100.0 | (80.6 - 100.0) | | 0.93 |
| Nadeem et al. (2024) | 3231 | 1007 | 33 | 2136 | 55 | 32.9 | (31.3 - 34.5) | 94.8 | (93.3 - 96.0) | 98.5 | (97.9 - 98.9) | 96.8 | (95.6 - 97.7) | 97.5 | (96.7 - 98.1) | | 0.96 |
| Nicolaes et al. (2023a) | 4810 | 593 | 285 | 3897 | 35 | 13.1 | (12.1 - 14.0) | 94.4 | (92.3 - 96.0) | 93.2 | (92.4 - 93.9) | 67.5 | (64.4 - 70.6) | 99.1 | (98.8 - 99.4) | | 0.79 |
| Nicolaes et al. (2023b) | 1943 | 240 | 91 | 1555 | 57 | 15.3 | (13.8 - 17.0) | 80.8 | (75.9 - 84.9) | 94.5 | (93.3 - 95.5) | 72.5 | (67.5 - 77.0) | 96.5 | (95.4 - 97.3) | | 0.76 |
| Page et al. (2023) | 1087 | 107 | 124 | 826 | 30 | 12.6 | (10.8 - 14.7) | 78.1 | (70.5 - 84.2) | 86.9 | (84.7 - 88.9) | 46.3 | (40.0 - 52.8) | 96.5 | (95.0 - 97.5) | | 0.58 |
| Pereira et al. (2024) | 899 | 107 | 55 | 699 | 38 | 16.1 | (13.9 - 18.7) | 73.8 | (66.1 - 80.3) | 92.7 | (90.6 - 94.4) | 66.0 | (58.5 - 72.9) | 94.8 | (93.0 - 96.2) | | 0.70 |
| Roux et al. (2022) | 500 | 119 | 131 | 242 | 8 | 25.4 | (21.8 - 29.4) | 93.7 | (88.1 - 96.8) | 64.9 | (59.9 - 69.5) | 47.6 | (41.5 - 53.8) | 96.8 | (93.8 - 98.4) | | 0.63 |
| Ruitenbeek et al. (2024) | 2036 | 150 | 88 | 1781 | 17 | 8.2 | (7.1 - 9.5) | 89.8 | (84.3 - 93.5) | 95.3 | (94.2 - 96.2) | 63.0 | (56.7 - 68.9) | 99.1 | (98.5 - 99.4) | | 0.74 |
| S Wang et al. (2022) | 1613 | 909 | 90 | 547 | 67 | 60.5 | (58.1 - 62.9) | 93.1 | (91.4 - 94.6) | 85.9 | (83.0 - 88.4) | 91.0 | (89.1 - 92.6) | 89.1 | (86.4 - 91.3) | | 0.92 |
| Seol et al. (2022) | 507 | 207 | 33 | 237 | 30 | 46.7 | (42.4 - 51.1) | 87.3 | (82.5 - 91.0) | 87.8 | (83.3 - 91.2) | 86.2 | (81.3 - 90.0) | 88.8 | (84.4 - 92.0) | | 0.87 |
| Shan et al. (2021) | 235 | 77 | 16 | 126 | 16 | 39.6 | (33.5 - 45.9) | 82.8 | (73.9 - 89.1) | 88.7 | (82.5 - 92.9) | 82.8 | (73.9 - 89.1) | 88.7 | (82.5 - 92.9) | | 0.83 |
| Small et al. (2021) | 665 | 109 | 17 | 505 | 34 | 21.5 | (18.5 - 24.8) | 76.2 | (68.6 - 82.5) | 96.7 | (94.8 - 98.0) | 86.5 | (79.5 - 91.4) | 93.7 | (91.3 - 95.5) | | 0.81 |
| Ukai et al. (2023) | 205 | 93 | 4 | 108 | 0 | 45.4 | (38.7 - 52.2) | 100.0 | (96.0 - 100.0) | 96.4 | (91.2 - 98.6) | 95.9 | (89.9 - 98.4) | 100.0 | (96.6 - 100.0) | | 0.98 |
| Voter et al. (2021) | 1904 | 67 | 106 | 1676 | 55 | 6.4 | (5.4 - 7.6) | 54.9 | (46.1 - 63.5) | 94.1 | (92.9 - 95.1) | 38.7 | (31.8 - 46.2) | 96.8 | (95.9 - 97.6) | | 0.45 |
| Weikert et al. (2020) | 510 | 139 | 30 | 321 | 20 | 31.2 | (27.3 - 35.3) | 87.4 | (81.4 - 91.7) | 91.5 | (88.1 - 93.9) | 82.2 | (75.8 - 87.3) | 94.1 | (91.1 - 96.2) | | 0.85 |
| Wu et al. (2021) | 8051 | 275 | 1138 | 6600 | 38 | 3.9 | (3.5 - 4.3) | 87.9 | (83.8 - 91.0) | 85.3 | (84.5 - 86.1) | 19.5 | (17.5 - 21.6) | 99.4 | (99.2 - 99.6) | | 0.32 |
| Yang et al. (2022) | 76 | 39 | 4 | 26 | 7 | 60.5 | (49.3 - 70.8) | 84.8 | (71.8 - 92.4) | 86.7 | (70.3 - 94.7) | 90.7 | (78.4 - 96.3) | 78.8 | (62.2 - 89.3) | | 0.88 |
| van den Wittenboer et al. (2024) | 2368 | 158 | 29 | 2118 | 63 | 9.3 | (8.2 - 10.6) | 71.5 | (65.2 - 77.0) | 98.6 | (98.1 - 99.1) | 84.5 | (78.6 - 89.0) | 97.1 | (96.3 - 97.7) | | 0.77 |
| Li et al. (2023) | 2319 | 2044 | 59 | 115 | 101 | 92.5 | (91.4 - 93.5) | 95.3 | (94.3 - 96.1) | 66.1 | (58.8 - 72.7) | 97.2 | (96.4 - 97.8) | 53.2 | (46.6 - 59.8) | | 0.96 |
| Liu et al. (2021) | 1179 | 356 | 41 | 577 | 205 | 47.6 | (44.7 - 50.4) | 63.5 | (59.4 - 67.3) | 93.4 | (91.1 - 95.1) | 89.7 | (86.3 - 92.3) | 73.8 | (70.6 - 76.7) | | 0.74 |
| Lu et al. (2024) | 4026 | 598 | 179 | 2827 | 422 | 25.3 | (24.0 - 26.7) | 58.6 | (55.6 - 61.6) | 94.0 | (93.1 - 94.8) | 77.0 | (73.9 - 79.8) | 87.0 | (85.8 - 88.1) | | 0.67 |
| S Wang et al. (2022) | 1613 | 734 | 40 | 597 | 242 | 60.5 | (58.1 - 62.9) | 75.2 | (72.4 - 77.8) | 93.7 | (91.6 - 95.4) | 94.8 | (93.0 - 96.2) | 71.2 | (68.0 - 74.1) | | 0.84 |
| Small et al. (2021) | 665 | 133 | 20 | 502 | 10 | 21.5 | (18.5 - 24.8) | 93.0 | (87.6 - 96.2) | 96.2 | (94.2 - 97.5) | 86.9 | (80.7 - 91.4) | 98.0 | (96.4 - 98.9) | | 0.90 |
| Yang et al. (2022) | 76 | 38 | 7 | 23 | 8 | 60.5 | (49.3 - 70.8) | 82.6 | (69.3 - 90.9) | 76.7 | (59.1 - 88.2) | 84.4 | (71.2 - 92.3) | 74.2 | (56.8 - 86.3) | | 0.84 |
| van den Wittenboer et al. (2024) | 2368 | 195 | 17 | 2130 | 26 | 9.3 | (8.2 - 10.6) | 88.2 | (83.3 - 91.8) | 99.2 | (98.7 - 99.5) | 92.0 | (87.5 - 94.9) | 98.8 | (98.2 - 99.2) | | 0.90 |
| Liu et al. (2021) | 1179 | 503 | 68 | 550 | 58 | 47.6 | (44.7 - 50.4) | 89.7 | (86.9 - 91.9) | 89.0 | (86.3 - 91.2) | 88.1 | (85.2 - 90.5) | 90.5 | (87.9 - 92.5) | | 0.89 |
| Lu et al. (2024) | 4026 | 840 | 77 | 2929 | 180 | 25.3 | (24.0 - 26.7) | 82.4 | (79.9 - 84.6) | 97.4 | (96.8 - 97.9) | 91.6 | (89.6 - 93.2) | 94.2 | (93.3 - 95.0) | | 0.87 |
| Yang et al. (2022) | 76 | 43 | 3 | 27 | 3 | 60.5 | (49.3 - 70.8) | 93.5 | (82.5 - 97.8) | 90.0 | (74.4 - 96.5) | 93.5 | (82.5 - 97.8) | 90.0 | (74.4 - 96.5) | | 0.93 |

Abbreviations: NPV = Negative Predictive Value; PPV = Positive Predictive Value; TP = True Positive; FP = False Positive; TN = True Negative; FN = False Negative

### Supplementary Table 6. Study-Level Diagnostic Performance Metrics Underlying the Forest Plots in Supplementary Figure 1 for Stand-Alone AI on Internal Test Datasets by Level of Analysis

| **Author**  **(Year)** | **N** | **TP** | **FP** | **TN** | **FN** | **Prevalence [%]** | | **Sensitivity [%]** | | | **Specificity [%]** | | **PPV [%]** | | **NPV [%]** | | **F1 score** |
| --- | --- | --- | --- | --- | --- | --- | --- | --- | --- | --- | --- | --- | --- | --- | --- | --- | --- |
| Bao et al. (2023) | 302 | 194 | 4 | 98 | 6 | 66.2 | (60.7 - 71.3) | 97.0 | (93.6 - 98.6) | 96.1 | | (90.3 - 98.5) | 98.0 | (94.9 - 99.2) | 94.2 | (88.0 - 97.3) | 0.97 |
| Erne et al. (2021) | 64 | 26 | 5 | 27 | 6 | 50.0 | (38.1 - 61.9) | 81.2 | (64.7 - 91.1) | 84.4 | | (68.2 - 93.1) | 83.9 | (67.4 - 92.9) | 81.8 | (65.6 - 91.4) | 0.83 |
| Tong et al. (2023) | 95 | 44 | 0 | 51 | 0 | 46.3 | (36.6 - 56.3) | 100.0 | (92.0 - 100.0) | 100.0 | | (93.0 - 100.0) | 100.0 | (92.0 - 100.0) | 100.0 | (93.0 - 100.0) | 1.00 |
| X Wang et al. (2022) | 3672 | 834 | 81 | 2705 | 52 | 24.1 | (22.8 - 25.5) | 94.1 | (92.4 - 95.5) | 97.1 | | (96.4 - 97.7) | 91.1 | (89.1 - 92.8) | 98.1 | (97.5 - 98.6) | 0.93 |
| Amodeo et al. (2021) | 30 | 19 | 0 | 5 | 6 | 83.3 | (66.4 - 92.7) | 76.0 | (56.6 - 88.5) | 100.0 | | (56.6 - 100.0) | 100.0 | (83.2 - 100.0) | 45.5 | (21.3 - 72.0) | 0.86 |
| Bao et al. (2023) | 302 | 195 | 1 | 101 | 5 | 66.2 | (60.7 - 71.3) | 97.5 | (94.3 - 98.9) | 99.0 | | (94.7 - 99.8) | 99.5 | (97.2 - 99.9) | 95.3 | (89.4 - 98.0) | 0.98 |
| Castro-Zunti et al. (2024) | 2000 | 748 | 123 | 877 | 252 | 50.0 | (47.8 - 52.2) | 74.8 | (72.0 - 77.4) | 87.7 | | (85.5 - 89.6) | 85.9 | (83.4 - 88.0) | 77.7 | (75.2 - 80.0) | 0.80 |
| Hu et al. (2021) | 252 | 80 | 36 | 128 | 8 | 34.9 | (29.3 - 41.0) | 90.9 | (83.1 - 95.3) | 78.0 | | (71.1 - 83.7) | 69.0 | (60.1 - 76.7) | 94.1 | (88.8 - 97.0) | 0.78 |
| Jeong et al. (2024) | 50 | 24 | 4 | 22 | 0 | 48.0 | (34.8 - 61.5) | 100.0 | (86.2 - 100.0) | 84.6 | | (66.5 - 93.8) | 85.7 | (68.5 - 94.3) | 100.0 | (85.1 - 100.0) | 0.92 |
| Li et al. (2023) | 1612 | 948 | 43 | 614 | 7 | 59.2 | (56.8 - 61.6) | 99.3 | (98.5 - 99.6) | 93.5 | | (91.3 - 95.1) | 95.7 | (94.2 - 96.8) | 98.9 | (97.7 - 99.5) | 0.97 |
| Lu et al. (2024) | 841 | 148 | 9 | 662 | 22 | 20.2 | (17.6 - 23.1) | 87.1 | (81.2 - 91.3) | 98.7 | | (97.5 - 99.3) | 94.3 | (89.5 - 97.0) | 96.8 | (95.2 - 97.9) | 0.91 |
| Moon et al. (2024) | 232 | 101 | 17 | 99 | 15 | 50.0 | (43.6 - 56.4) | 87.1 | (79.8 - 92.0) | 85.3 | | (77.8 - 90.6) | 85.6 | (78.1 - 90.8) | 86.8 | (79.4 - 91.9) | 0.86 |
| Moon et al. (2022) | 40 | 21 | 3 | 16 | 0 | 52.5 | (37.5 - 67.1) | 100.0 | (84.5 - 100.0) | 84.2 | | (62.4 - 94.5) | 87.5 | (69.0 - 95.7) | 100.0 | (80.6 - 100.0) | 0.93 |
| S Wang et al. (2022) | 1628 | 408 | 207 | 1001 | 12 | 25.8 | (23.7 - 28.0) | 97.1 | (95.1 - 98.4) | 82.9 | | (80.6 - 84.9) | 66.3 | (62.5 - 70.0) | 98.8 | (97.9 - 99.3) | 0.79 |
| Seol et al. (2022) | 507 | 207 | 33 | 237 | 30 | 46.7 | (42.4 - 51.1) | 87.3 | (82.5 - 91.0) | 87.8 | | (83.3 - 91.2) | 86.2 | (81.3 - 90.0) | 88.8 | (84.4 - 92.0) | 0.87 |
| Ukai et al. (2023) | 205 | 93 | 4 | 108 | 0 | 45.4 | (38.7 - 52.2) | 100.0 | (96.0 - 100.0) | 96.4 | | (91.2 - 98.6) | 95.9 | (89.9 - 98.4) | 100.0 | (96.6 - 100.0) | 0.98 |
| Wu et al. (2021) | 8051 | 275 | 1138 | 6600 | 38 | 3.9 | (3.5 - 4.3) | 87.9 | (83.8 - 91.0) | 85.3 | | (84.5 - 86.1) | 19.5 | (17.5 - 21.6) | 99.4 | (99.2 - 99.6) | 0.32 |
| Yang et al. (2022) | 76 | 39 | 4 | 26 | 7 | 60.5 | (49.3 - 70.8) | 84.8 | (71.8 - 92.4) | 86.7 | | (70.3 - 94.7) | 90.7 | (78.4 - 96.3) | 78.8 | (62.2 - 89.3) | 0.88 |
| Al-Helo et al. (2012) | 250 | 21 | 3 | 224 | 2 | 9.2 | (6.2 - 13.4) | 91.3 | (73.2 - 97.6) | 98.7 | | (96.2 - 99.5) | 87.5 | (69.0 - 95.7) | 99.1 | (96.8 - 99.8) | 0.89 |
| Lee et al. (2024) | 981 | 82 | 36 | 846 | 17 | 10.1 | (8.4 - 12.1) | 82.8 | (74.2 - 89.0) | 95.9 | | (94.4 - 97.0) | 69.5 | (60.7 - 77.1) | 98.0 | (96.9 - 98.8) | 0.76 |
| Polzer et al. (2024) | 448 | 73 | 77 | 275 | 23 | 21.4 | (17.9 - 25.5) | 76.0 | (66.6 - 83.5) | 78.1 | | (73.5 - 82.1) | 48.7 | (40.8 - 56.6) | 92.3 | (88.7 - 94.8) | 0.59 |
| Tian et al. (2024) | 152 | 32 | 5 | 115 | 0 | 21.1 | (15.3 - 28.2) | 100.0 | (89.3 - 100.0) | 95.8 | | (90.6 - 98.2) | 86.5 | (72.0 - 94.1) | 100.0 | (96.8 - 100.0) | 0.93 |
| Zhang et al. (2023) | 11356 | 356 | 181 | 10801 | 18 | 3.3 | (3.0 - 3.6) | 95.2 | (92.5 - 96.9) | 98.4 | | (98.1 - 98.6) | 66.3 | (62.2 - 70.2) | 99.8 | (99.7 - 99.9) | 0.78 |
| S Wang et al. (2022) | 39072 | 1553 | 599 | 36808 | 112 | 4.3 | (4.1 - 4.5) | 93.3 | (92.0 - 94.4) | 98.4 | | (98.3 - 98.5) | 72.2 | (70.2 - 74.0) | 99.7 | (99.6 - 99.7) | 0.81 |

Abbreviations: NPV = Negative Predictive Value; PPV = Positive Predictive Value; TP = True Positive; FP = False Positive; TN = True Negative; FN = False Negative

### Supplementary Table 7. Study-Level Diagnostic Performance Metrics Underlying the Forest Plots in Supplementary Figure 2 for Stand-Alone AI (Patient-Wise Level) by Reference Standard

| **Author**  **(Year)** | **N** | **TP** | **FP** | **TN** | **FN** | **Prevalence [%]** | | **Sensitivity [%]** | | | **Specificity [%]** | | | **PPV [%]** | | | **NPV [%]** | | **F1 score** | |
| --- | --- | --- | --- | --- | --- | --- | --- | --- | --- | --- | --- | --- | --- | --- | --- | --- | --- | --- | --- | --- |
| Amodeo et al. (2021) | 30 | 19 | 0 | 5 | 6 | 83.3 | (66.4 - 92.7) | 76.0 | (56.6 - 88.5) | 100.0 | | (56.6 - 100.0) | 100.0 | | (83.2 - 100.0) | 45.5 | | (21.3 - 72.0) | 0.86 |  |
| Bao et al. (2023) | 302 | 195 | 1 | 101 | 5 | 66.2 | (60.7 - 71.3) | 97.5 | (94.3 - 98.9) | 99.0 | | (94.7 - 99.8) | 99.5 | | (97.2 - 99.9) | 95.3 | | (89.4 - 98.0) | 0.98 |  |
| H Wang et al. (2023) | 192 | 86 | 5 | 90 | 11 | 50.5 | (43.5 - 57.5) | 88.7 | (80.8 - 93.5) | 94.7 | | (88.3 - 97.7) | 94.5 | | (87.8 - 97.6) | 89.1 | | (81.5 - 93.8) | 0.91 |  |
| Kolanu et al. (2020) | 1570 | 183 | 97 | 1191 | 99 | 18.0 | (16.1 - 19.9) | 64.9 | (59.2 - 70.2) | 92.5 | | (90.9 - 93.8) | 65.4 | | (59.6 - 70.7) | 92.3 | | (90.7 - 93.7) | 0.65 |  |
| Li et al. (2023) | 2319 | 2122 | 35 | 139 | 23 | 92.5 | (91.4 - 93.5) | 98.9 | (98.4 - 99.3) | 79.9 | | (73.3 - 85.2) | 98.4 | | (97.8 - 98.8) | 85.8 | | (79.6 - 90.3) | 0.99 |  |
| Nicolaes et al. (2023a) | 4810 | 593 | 285 | 3897 | 35 | 13.1 | (12.1 - 14.0) | 94.4 | (92.3 - 96.0) | 93.2 | | (92.4 - 93.9) | 67.5 | | (64.4 - 70.6) | 99.1 | | (98.8 - 99.4) | 0.79 |  |
| Page et al. (2023) | 1087 | 107 | 124 | 826 | 30 | 12.6 | (10.8 - 14.7) | 78.1 | (70.5 - 84.2) | 86.9 | | (84.7 - 88.9) | 46.3 | | (40.0 - 52.8) | 96.5 | | (95.0 - 97.5) | 0.58 |  |
| Pereira et al. (2024) | 899 | 107 | 55 | 699 | 38 | 16.1 | (13.9 - 18.7) | 73.8 | (66.1 - 80.3) | 92.7 | | (90.6 - 94.4) | 66.0 | | (58.5 - 72.9) | 94.8 | | (93.0 - 96.2) | 0.70 |  |
| Roux et al. (2022) | 500 | 119 | 131 | 242 | 8 | 25.4 | (21.8 - 29.4) | 93.7 | (88.1 - 96.8) | 64.9 | | (59.9 - 69.5) | 47.6 | | (41.5 - 53.8) | 96.8 | | (93.8 - 98.4) | 0.63 |  |
| S Wang et al. (2022) | 1613 | 909 | 90 | 547 | 67 | 60.5 | (58.1 - 62.9) | 93.1 | (91.4 - 94.6) | 85.9 | | (83.0 - 88.4) | 91.0 | | (89.1 - 92.6) | 89.1 | | (86.4 - 91.3) | 0.92 |  |
| Small et al. (2021) | 665 | 109 | 17 | 505 | 34 | 21.5 | (18.5 - 24.8) | 76.2 | (68.6 - 82.5) | 96.7 | | (94.8 - 98.0) | 86.5 | | (79.5 - 91.4) | 93.7 | | (91.3 - 95.5) | 0.81 |  |
| Yang et al. (2022) | 76 | 39 | 4 | 26 | 7 | 60.5 | (49.3 - 70.8) | 84.8 | (71.8 - 92.4) | 86.7 | | (70.3 - 94.7) | 90.7 | | (78.4 - 96.3) | 78.8 | | (62.2 - 89.3) | 0.88 |  |
| van den Wittenboer et al. (2024) | 2368 | 158 | 29 | 2118 | 63 | 9.3 | (8.2 - 10.6) | 71.5 | (65.2 - 77.0) | 98.6 | | (98.1 - 99.1) | 84.5 | | (78.6 - 89.0) | 97.1 | | (96.3 - 97.7) | 0.77 |  |
| Bendtsen et al. (2024) | 1000 | 65 | 81 | 824 | 30 | 9.5 | (7.8 - 11.5) | 68.4 | (58.5 - 76.9) | 91.0 | | (89.0 - 92.7) | 44.5 | | (36.7 - 52.6) | 96.5 | | (95.0 - 97.5) | 0.54 |  |
| Castro-Zunti et al. (2024) | 2000 | 748 | 123 | 877 | 252 | 50.0 | (47.8 - 52.2) | 74.8 | (72.0 - 77.4) | 87.7 | | (85.5 - 89.6) | 85.9 | | (83.4 - 88.0) | 77.7 | | (75.2 - 80.0) | 0.80 |  |
| Hu et al. (2021) | 252 | 80 | 36 | 128 | 8 | 34.9 | (29.3 - 41.0) | 90.9 | (83.1 - 95.3) | 78.0 | | (71.1 - 83.7) | 69.0 | | (60.1 - 76.7) | 94.1 | | (88.8 - 97.0) | 0.78 |  |
| Jeong et al. (2024) | 50 | 24 | 4 | 22 | 0 | 48.0 | (34.8 - 61.5) | 100.0 | (86.2 - 100.0) | 84.6 | | (66.5 - 93.8) | 85.7 | | (68.5 - 94.3) | 100.0 | | (85.1 - 100.0) | 0.92 |  |
| Lu et al. (2024) | 841 | 148 | 9 | 662 | 22 | 20.2 | (17.6 - 23.1) | 87.1 | (81.2 - 91.3) | 98.7 | | (97.5 - 99.3) | 94.3 | | (89.5 - 97.0) | 96.8 | | (95.2 - 97.9) | 0.91 |  |
| Moon et al. (2022) | 232 | 101 | 17 | 99 | 15 | 50.0 | (43.6 - 56.4) | 87.1 | (79.8 - 92.0) | 85.3 | | (77.8 - 90.6) | 85.6 | | (78.1 - 90.8) | 86.8 | | (79.4 - 91.9) | 0.86 |  |
| Moon et al. (2024) | 40 | 21 | 3 | 16 | 0 | 52.5 | (37.5 - 67.1) | 100.0 | (84.5 - 100.0) | 84.2 | | (62.4 - 94.5) | 87.5 | | (69.0 - 95.7) | 100.0 | | (80.6 - 100.0) | 0.93 |  |
| Nadeem et al. (2024) | 3231 | 1007 | 33 | 2136 | 55 | 32.9 | (31.3 - 34.5) | 94.8 | (93.3 - 96.0) | 98.5 | | (97.9 - 98.9) | 96.8 | | (95.6 - 97.7) | 97.5 | | (96.7 - 98.1) | 0.96 |  |
| Nicolaes et al. (2023b) | 1943 | 240 | 91 | 1555 | 57 | 15.3 | (13.8 - 17.0) | 80.8 | (75.9 - 84.9) | 94.5 | | (93.3 - 95.5) | 72.5 | | (67.5 - 77.0) | 96.5 | | (95.4 - 97.3) | 0.76 |  |
| Ruitenbeek et al. (2024) | 2036 | 150 | 88 | 1781 | 17 | 8.2 | (7.1 - 9.5) | 89.8 | (84.3 - 93.5) | 95.3 | | (94.2 - 96.2) | 63.0 | | (56.7 - 68.9) | 99.1 | | (98.5 - 99.4) | 0.74 |  |
| Seol et al. (2022) | 507 | 207 | 33 | 237 | 30 | 46.7 | (42.4 - 51.1) | 87.3 | (82.5 - 91.0) | 87.8 | | (83.3 - 91.2) | 86.2 | | (81.3 - 90.0) | 88.8 | | (84.4 - 92.0) | 0.87 |  |
| Shan et al. (2021) | 235 | 77 | 16 | 126 | 16 | 39.6 | (33.5 - 45.9) | 82.8 | (73.9 - 89.1) | 88.7 | | (82.5 - 92.9) | 82.8 | | (73.9 - 89.1) | 88.7 | | (82.5 - 92.9) | 0.83 |  |
| Ukai et al. (2023) | 205 | 93 | 4 | 108 | 0 | 45.4 | (38.7 - 52.2) | 100.0 | (96.0 - 100.0) | 96.4 | | (91.2 - 98.6) | 95.9 | | (89.9 - 98.4) | 100.0 | | (96.6 - 100.0) | 0.98 |  |
| Voter et al. (2021) | 1904 | 67 | 106 | 1676 | 55 | 6.4 | (5.4 - 7.6) | 54.9 | (46.1 - 63.5) | 94.1 | | (92.9 - 95.1) | 38.7 | | (31.8 - 46.2) | 96.8 | | (95.9 - 97.6) | 0.45 |  |
| Weikert et al. (2020) | 510 | 139 | 30 | 321 | 20 | 31.2 | (27.3 - 35.3) | 87.4 | (81.4 - 91.7) | 91.5 | | (88.1 - 93.9) | 82.2 | | (75.8 - 87.3) | 94.1 | | (91.1 - 96.2) | 0.85 |  |
| Wu et al. (2021) | 8051 | 275 | 1138 | 6600 | 38 | 3.9 | (3.5 - 4.3) | 87.9 | (83.8 - 91.0) | 85.3 | | (84.5 - 86.1) | 19.5 | | (17.5 - 21.6) | 99.4 | | (99.2 - 99.6) | 0.32 |  |

Abbreviations: NPV = Negative Predictive Value; PPV = Positive Predictive Value; TP = True Positive; FP = False Positive; TN = True Negative; FN = False Negative

### Supplementary Table 8. Study-Level Diagnostic Performance Metrics Underlying the Forest Plots in Supplementary Figure 3 for Stand-Alone AI (Patient-Wise Level) by Risk of Bias

| **Author**  **(Year)** | **N** | **TP** | **FP** | **TN** | **FN** | **Prevalence [%]** | | **Sensitivity [%]** | | **Specificity [%]** | | **PPV [%]** | | **NPV [%]** | | **F1 score** |
| --- | --- | --- | --- | --- | --- | --- | --- | --- | --- | --- | --- | --- | --- | --- | --- | --- |
| Bao et al. (2023) | 302 | 195 | 1 | 101 | 5 | 66.2 | (60.7 - 71.3) | 97.5 | (94.3 - 98.9) | 99.0 | (94.7 - 99.8) | 99.5 | (97.2 - 99.9) | 95.3 | (89.4 - 98.0) | 0.98 |
| Roux et al. (2022) | 500 | 119 | 131 | 242 | 8 | 25.4 | (21.8 - 29.4) | 93.7 | (88.1 - 96.8) | 64.9 | (59.9 - 69.5) | 47.6 | (41.5 - 53.8) | 96.8 | (93.8 - 98.4) | 0.63 |
| Yang et al. (2022) | 76 | 39 | 4 | 26 | 7 | 60.5 | (49.3 - 70.8) | 84.8 | (71.8 - 92.4) | 86.7 | (70.3 - 94.7) | 90.7 | (78.4 - 96.3) | 78.8 | (62.2 - 89.3) | 0.88 |
| Amodeo et al. (2021) | 30 | 19 | 0 | 5 | 6 | 83.3 | (66.4 - 92.7) | 76.0 | (56.6 - 88.5) | 100.0 | (56.6 - 100.0) | 100.0 | (83.2 - 100.0) | 45.5 | (21.3 - 72.0) | 0.86 |
| Castro-Zunti et al. (2024) | 2000 | 748 | 123 | 877 | 252 | 50.0 | (47.8 - 52.2) | 74.8 | (72.0 - 77.4) | 87.7 | (85.5 - 89.6) | 85.9 | (83.4 - 88.0) | 77.7 | (75.2 - 80.0) | 0.80 |
| H Wang et al. (2023) | 192 | 86 | 5 | 90 | 11 | 50.5 | (43.5 - 57.5) | 88.7 | (80.8 - 93.5) | 94.7 | (88.3 - 97.7) | 94.5 | (87.8 - 97.6) | 89.1 | (81.5 - 93.8) | 0.91 |
| Hu et al. (2021) | 252 | 80 | 36 | 128 | 8 | 34.9 | (29.3 - 41.0) | 90.9 | (83.1 - 95.3) | 78.0 | (71.1 - 83.7) | 69.0 | (60.1 - 76.7) | 94.1 | (88.8 - 97.0) | 0.78 |
| Jeong et al. (2024) | 50 | 24 | 4 | 22 | 0 | 48.0 | (34.8 - 61.5) | 100.0 | (86.2 - 100.0) | 84.6 | (66.5 - 93.8) | 85.7 | (68.5 - 94.3) | 100.0 | (85.1 - 100.0) | 0.92 |
| Li et al. (2023) | 2319 | 2122 | 35 | 139 | 23 | 92.5 | (91.4 - 93.5) | 98.9 | (98.4 - 99.3) | 79.9 | (73.3 - 85.2) | 98.4 | (97.8 - 98.8) | 85.8 | (79.6 - 90.3) | 0.99 |
| Lu et al. (2024) | 841 | 148 | 9 | 662 | 22 | 20.2 | (17.6 - 23.1) | 87.1 | (81.2 - 91.3) | 98.7 | (97.5 - 99.3) | 94.3 | (89.5 - 97.0) | 96.8 | (95.2 - 97.9) | 0.91 |
| Moon et al. (2022) | 232 | 101 | 17 | 99 | 15 | 50.0 | (43.6 - 56.4) | 87.1 | (79.8 - 92.0) | 85.3 | (77.8 - 90.6) | 85.6 | (78.1 - 90.8) | 86.8 | (79.4 - 91.9) | 0.86 |
| Moon et al. (2024) | 40 | 21 | 3 | 16 | 0 | 52.5 | (37.5 - 67.1) | 100.0 | (84.5 - 100.0) | 84.2 | (62.4 - 94.5) | 87.5 | (69.0 - 95.7) | 100.0 | (80.6 - 100.0) | 0.93 |
| Nadeem et al. (2024) | 3231 | 1007 | 33 | 2136 | 55 | 32.9 | (31.3 - 34.5) | 94.8 | (93.3 - 96.0) | 98.5 | (97.9 - 98.9) | 96.8 | (95.6 - 97.7) | 97.5 | (96.7 - 98.1) | 0.96 |
| Nicolaes et al. (2023a) | 4810 | 593 | 285 | 3897 | 35 | 13.1 | (12.1 - 14.0) | 94.4 | (92.3 - 96.0) | 93.2 | (92.4 - 93.9) | 67.5 | (64.4 - 70.6) | 99.1 | (98.8 - 99.4) | 0.79 |
| Page et al. (2023) | 1087 | 107 | 124 | 826 | 30 | 12.6 | (10.8 - 14.7) | 78.1 | (70.5 - 84.2) | 86.9 | (84.7 - 88.9) | 46.3 | (40.0 - 52.8) | 96.5 | (95.0 - 97.5) | 0.58 |
| Pereira et al. (2024) | 899 | 107 | 55 | 699 | 38 | 16.1 | (13.9 - 18.7) | 73.8 | (66.1 - 80.3) | 92.7 | (90.6 - 94.4) | 66.0 | (58.5 - 72.9) | 94.8 | (93.0 - 96.2) | 0.70 |
| Ruitenbeek et al. (2024) | 2036 | 150 | 88 | 1781 | 17 | 8.2 | (7.1 - 9.5) | 89.8 | (84.3 - 93.5) | 95.3 | (94.2 - 96.2) | 63.0 | (56.7 - 68.9) | 99.1 | (98.5 - 99.4) | 0.74 |
| S Wang et al. (2022) | 1613 | 909 | 90 | 547 | 67 | 60.5 | (58.1 - 62.9) | 93.1 | (91.4 - 94.6) | 85.9 | (83.0 - 88.4) | 91.0 | (89.1 - 92.6) | 89.1 | (86.4 - 91.3) | 0.92 |
| Seol et al. (2022) | 507 | 207 | 33 | 237 | 30 | 46.7 | (42.4 - 51.1) | 87.3 | (82.5 - 91.0) | 87.8 | (83.3 - 91.2) | 86.2 | (81.3 - 90.0) | 88.8 | (84.4 - 92.0) | 0.87 |
| Shan et al. (2021) | 235 | 77 | 16 | 126 | 16 | 39.6 | (33.5 - 45.9) | 82.8 | (73.9 - 89.1) | 88.7 | (82.5 - 92.9) | 82.8 | (73.9 - 89.1) | 88.7 | (82.5 - 92.9) | 0.83 |
| Small et al. (2021) | 665 | 109 | 17 | 505 | 34 | 21.5 | (18.5 - 24.8) | 76.2 | (68.6 - 82.5) | 96.7 | (94.8 - 98.0) | 86.5 | (79.5 - 91.4) | 93.7 | (91.3 - 95.5) | 0.81 |
| Ukai et al. (2023) | 205 | 93 | 4 | 108 | 0 | 45.4 | (38.7 - 52.2) | 100.0 | (96.0 - 100.0) | 96.4 | (91.2 - 98.6) | 95.9 | (89.9 - 98.4) | 100.0 | (96.6 - 100.0) | 0.98 |
| Voter et al. (2021) | 1904 | 67 | 106 | 1676 | 55 | 6.4 | (5.4 - 7.6) | 54.9 | (46.1 - 63.5) | 94.1 | (92.9 - 95.1) | 38.7 | (31.8 - 46.2) | 96.8 | (95.9 - 97.6) | 0.45 |
| Weikert et al. (2020) | 510 | 139 | 30 | 321 | 20 | 31.2 | (27.3 - 35.3) | 87.4 | (81.4 - 91.7) | 91.5 | (88.1 - 93.9) | 82.2 | (75.8 - 87.3) | 94.1 | (91.1 - 96.2) | 0.85 |
| Wu et al. (2021) | 8051 | 275 | 1138 | 6600 | 38 | 3.9 | (3.5 - 4.3) | 87.9 | (83.8 - 91.0) | 85.3 | (84.5 - 86.1) | 19.5 | (17.5 - 21.6) | 99.4 | (99.2 - 99.6) | 0.32 |
| van den Wittenboer et al. (2024) | 2368 | 158 | 29 | 2118 | 63 | 9.3 | (8.2 - 10.6) | 71.5 | (65.2 - 77.0) | 98.6 | (98.1 - 99.1) | 84.5 | (78.6 - 89.0) | 97.1 | (96.3 - 97.7) | 0.77 |
| Bendtsen et al. (2024) | 1000 | 65 | 81 | 824 | 30 | 9.5 | (7.8 - 11.5) | 68.4 | (58.5 - 76.9) | 91.0 | (89.0 - 92.7) | 44.5 | (36.7 - 52.6) | 96.5 | (95.0 - 97.5) | 0.54 |
| Kolanu et al. (2020) | 1570 | 183 | 97 | 1191 | 99 | 18.0 | (16.1 - 19.9) | 64.9 | (59.2 - 70.2) | 92.5 | (90.9 - 93.8) | 65.4 | (59.6 - 70.7) | 92.3 | (90.7 - 93.7) | 0.65 |
| Nicolaes et al. (2023b) | 1943 | 240 | 91 | 1555 | 57 | 15.3 | (13.8 - 17.0) | 80.8 | (75.9 - 84.9) | 94.5 | (93.3 - 95.5) | 72.5 | (67.5 - 77.0) | 96.5 | (95.4 - 97.3) | 0.76 |

Abbreviations: NPV = Negative Predictive Value; PPV = Positive Predictive Value; TP = True Positive; FP = False Positive; TN = True Negative; FN = False Negative

### Supplementary Table 9. Study-Level Diagnostic Performance Metrics Underlying the Forest Plots in Supplementary Figure 4 for Stand-Alone AI (Patient-Wise Level) by Funding

| **Author**  **(Year)** | **N** | **TP** | **FP** | **TN** | **FN** | **Prevalence [%]** | | **Sensitivity [%]** | | **Specificity [%]** | | **PPV [%]** | | **NPV [%]** | | **F1 score** |
| --- | --- | --- | --- | --- | --- | --- | --- | --- | --- | --- | --- | --- | --- | --- | --- | --- |
| Bendtsen et al. (2024) | 1000 | 65 | 81 | 824 | 30 | 9.5 | (7.8 - 11.5) | 68.4 | (58.5 - 76.9) | 91.0 | (89.0 - 92.7) | 44.5 | (36.7 - 52.6) | 96.5 | (95.0 - 97.5) | 0.54 |
| Kolanu et al. (2020) | 1570 | 183 | 97 | 1191 | 99 | 18.0 | (16.1 - 19.9) | 64.9 | (59.2 - 70.2) | 92.5 | (90.9 - 93.8) | 65.4 | (59.6 - 70.7) | 92.3 | (90.7 - 93.7) | 0.65 |
| Nicolaes et al. (2023b) | 1943 | 240 | 91 | 1555 | 57 | 15.3 | (13.8 - 17.0) | 80.8 | (75.9 - 84.9) | 94.5 | (93.3 - 95.5) | 72.5 | (67.5 - 77.0) | 96.5 | (95.4 - 97.3) | 0.76 |
| Nicolaes et al. (2023a) | 4810 | 593 | 285 | 3897 | 35 | 13.1 | (12.1 - 14.0) | 94.4 | (92.3 - 96.0) | 93.2 | (92.4 - 93.9) | 67.5 | (64.4 - 70.6) | 99.1 | (98.8 - 99.4) | 0.79 |
| Page et al. (2023) | 1087 | 107 | 124 | 826 | 30 | 12.6 | (10.8 - 14.7) | 78.1 | (70.5 - 84.2) | 86.9 | (84.7 - 88.9) | 46.3 | (40.0 - 52.8) | 96.5 | (95.0 - 97.5) | 0.58 |
| Amodeo et al. (2021) | 30 | 19 | 0 | 5 | 6 | 83.3 | (66.4 - 92.7) | 76.0 | (56.6 - 88.5) | 100.0 | (56.6 - 100.0) | 100.0 | (83.2 - 100.0) | 45.5 | (21.3 - 72.0) | 0.86 |
| Bao et al. (2023) | 302 | 195 | 1 | 101 | 5 | 66.2 | (60.7 - 71.3) | 97.5 | (94.3 - 98.9) | 99.0 | (94.7 - 99.8) | 99.5 | (97.2 - 99.9) | 95.3 | (89.4 - 98.0) | 0.98 |
| Castro-Zunti et al. (2024) | 2000 | 748 | 123 | 877 | 252 | 50.0 | (47.8 - 52.2) | 74.8 | (72.0 - 77.4) | 87.7 | (85.5 - 89.6) | 85.9 | (83.4 - 88.0) | 77.7 | (75.2 - 80.0) | 0.80 |
| H Wang et al. (2023) | 192 | 86 | 5 | 90 | 11 | 50.5 | (43.5 - 57.5) | 88.7 | (80.8 - 93.5) | 94.7 | (88.3 - 97.7) | 94.5 | (87.8 - 97.6) | 89.1 | (81.5 - 93.8) | 0.91 |
| Hu et al. (2021) | 252 | 80 | 36 | 128 | 8 | 34.9 | (29.3 - 41.0) | 90.9 | (83.1 - 95.3) | 78.0 | (71.1 - 83.7) | 69.0 | (60.1 - 76.7) | 94.1 | (88.8 - 97.0) | 0.78 |
| Jeong et al. (2024) | 50 | 24 | 4 | 22 | 0 | 48.0 | (34.8 - 61.5) | 100.0 | (86.2 - 100.0) | 84.6 | (66.5 - 93.8) | 85.7 | (68.5 - 94.3) | 100.0 | (85.1 - 100.0) | 0.92 |
| Li et al. (2023) | 2319 | 2122 | 35 | 139 | 23 | 92.5 | (91.4 - 93.5) | 98.9 | (98.4 - 99.3) | 79.9 | (73.3 - 85.2) | 98.4 | (97.8 - 98.8) | 85.8 | (79.6 - 90.3) | 0.99 |
| Lu et al. (2024) | 841 | 148 | 9 | 662 | 22 | 20.2 | (17.6 - 23.1) | 87.1 | (81.2 - 91.3) | 98.7 | (97.5 - 99.3) | 94.3 | (89.5 - 97.0) | 96.8 | (95.2 - 97.9) | 0.91 |
| Moon et al. (2022) | 232 | 101 | 17 | 99 | 15 | 50.0 | (43.6 - 56.4) | 87.1 | (79.8 - 92.0) | 85.3 | (77.8 - 90.6) | 85.6 | (78.1 - 90.8) | 86.8 | (79.4 - 91.9) | 0.86 |
| Moon et al. (2024) | 40 | 21 | 3 | 16 | 0 | 52.5 | (37.5 - 67.1) | 100.0 | (84.5 - 100.0) | 84.2 | (62.4 - 94.5) | 87.5 | (69.0 - 95.7) | 100.0 | (80.6 - 100.0) | 0.93 |
| Nadeem et al. (2024) | 3231 | 1007 | 33 | 2136 | 55 | 32.9 | (31.3 - 34.5) | 94.8 | (93.3 - 96.0) | 98.5 | (97.9 - 98.9) | 96.8 | (95.6 - 97.7) | 97.5 | (96.7 - 98.1) | 0.96 |
| Pereira et al. (2024) | 899 | 107 | 55 | 699 | 38 | 16.1 | (13.9 - 18.7) | 73.8 | (66.1 - 80.3) | 92.7 | (90.6 - 94.4) | 66.0 | (58.5 - 72.9) | 94.8 | (93.0 - 96.2) | 0.70 |
| Roux et al. (2022) | 500 | 119 | 131 | 242 | 8 | 25.4 | (21.8 - 29.4) | 93.7 | (88.1 - 96.8) | 64.9 | (59.9 - 69.5) | 47.6 | (41.5 - 53.8) | 96.8 | (93.8 - 98.4) | 0.63 |
| Ruitenbeek et al. (2024) | 2036 | 150 | 88 | 1781 | 17 | 8.2 | (7.1 - 9.5) | 89.8 | (84.3 - 93.5) | 95.3 | (94.2 - 96.2) | 63.0 | (56.7 - 68.9) | 99.1 | (98.5 - 99.4) | 0.74 |
| S Wang et al. (2022) | 1613 | 909 | 90 | 547 | 67 | 60.5 | (58.1 - 62.9) | 93.1 | (91.4 - 94.6) | 85.9 | (83.0 - 88.4) | 91.0 | (89.1 - 92.6) | 89.1 | (86.4 - 91.3) | 0.92 |
| Seol et al. (2022) | 507 | 207 | 33 | 237 | 30 | 46.7 | (42.4 - 51.1) | 87.3 | (82.5 - 91.0) | 87.8 | (83.3 - 91.2) | 86.2 | (81.3 - 90.0) | 88.8 | (84.4 - 92.0) | 0.87 |
| Shan et al. (2021) | 235 | 77 | 16 | 126 | 16 | 39.6 | (33.5 - 45.9) | 82.8 | (73.9 - 89.1) | 88.7 | (82.5 - 92.9) | 82.8 | (73.9 - 89.1) | 88.7 | (82.5 - 92.9) | 0.83 |
| Small et al. (2021) | 665 | 109 | 17 | 505 | 34 | 21.5 | (18.5 - 24.8) | 76.2 | (68.6 - 82.5) | 96.7 | (94.8 - 98.0) | 86.5 | (79.5 - 91.4) | 93.7 | (91.3 - 95.5) | 0.81 |
| Ukai et al. (2023) | 205 | 93 | 4 | 108 | 0 | 45.4 | (38.7 - 52.2) | 100.0 | (96.0 - 100.0) | 96.4 | (91.2 - 98.6) | 95.9 | (89.9 - 98.4) | 100.0 | (96.6 - 100.0) | 0.98 |
| Voter et al. (2021) | 1904 | 67 | 106 | 1676 | 55 | 6.4 | (5.4 - 7.6) | 54.9 | (46.1 - 63.5) | 94.1 | (92.9 - 95.1) | 38.7 | (31.8 - 46.2) | 96.8 | (95.9 - 97.6) | 0.45 |
| Weikert et al. (2020) | 510 | 139 | 30 | 321 | 20 | 31.2 | (27.3 - 35.3) | 87.4 | (81.4 - 91.7) | 91.5 | (88.1 - 93.9) | 82.2 | (75.8 - 87.3) | 94.1 | (91.1 - 96.2) | 0.85 |
| Wu et al. (2021) | 8051 | 275 | 1138 | 6600 | 38 | 3.9 | (3.5 - 4.3) | 87.9 | (83.8 - 91.0) | 85.3 | (84.5 - 86.1) | 19.5 | (17.5 - 21.6) | 99.4 | (99.2 - 99.6) | 0.32 |
| Yang et al. (2022) | 76 | 39 | 4 | 26 | 7 | 60.5 | (49.3 - 70.8) | 84.8 | (71.8 - 92.4) | 86.7 | (70.3 - 94.7) | 90.7 | (78.4 - 96.3) | 78.8 | (62.2 - 89.3) | 0.88 |
| van den Wittenboer et al. (2024) | 2368 | 158 | 29 | 2118 | 63 | 9.3 | (8.2 - 10.6) | 71.5 | (65.2 - 77.0) | 98.6 | (98.1 - 99.1) | 84.5 | (78.6 - 89.0) | 97.1 | (96.3 - 97.7) | 0.77 |

Abbreviations: NPV = Negative Predictive Value; PPV = Positive Predictive Value; TP = True Positive; FP = False Positive; TN = True Negative; FN = False Negative
