# Supplementary material for: Artificial intelligence for fracture detection on computed tomography: a comprehensive systematic review and meta-analysis of diagnostic test accuracy in non-commercial and commercial solutions
Source: Emerg Radiol. 2026 Feb 7;33(2):351–75. doi: 10.1007/s10140-026-02437-7 (PMC13079534; doi:10.1007/s10140-026-02437-7)
Supplement: Supplementary file 2 — (DOCX 34.8 KB) [file 10140_2026_2437_MOESM2_ESM.docx]

# STATA 18.1 analytical code - Artificial intelligence for fracture detection on computed tomography: A comprehensive systematic review and meta-analysis of diagnostic test accuracy in non-commercial and commercial solutions

## Main do-file

*** CLEAR ALL, SET WORKING DIRECTORY AND LOAD HELPFILES

clear all

** Working directory has to be changed

cd ".../SR_AI_CT/"

qui do "helpfiles/Do_helpfiles_DTA_SR.do"

*** SET PARAMETERS

global scale = 0.2

global hold = 1

local year = year(date(c(current_date),"DMY",2000))

local month = month(date(c(current_date),"DMY",2000))

local day = day(date(c(current_date),"DMY",2000))

if length("`month'") == 1 local month = "0"+"`month'"

if length("`day'") == 1 local day = "0"+"`day'"

local sav_name = "`year'.`month'.`day'.AICT_"

global sav_name = "`sav_name'"

capture rm "`sav_name'.xlsx"

set scheme stcolor

*** IMPORT & CLEAN

import_dataset, filename("input/2025_04_15_KI_CT_DTA_Data_Final.xlsx") type_on(0)

encode anatomical_subregion, gen(anatomical_subregion_enc)

label var anatomical_subregion_enc "Anatomical Subregion"

rename (true_positives false_positives false_negatives true_negatives) (tp fp fn tn)

local et_al " et al. "

local et_al " "

gen author_year = author + "`et_al'" + "(" + string(year) + ")"

replace author_year = "Nicolaes (2023a)" if study_id == 15

replace author_year = "Nicolaes (2023b)" if study_id == 25

label var author_year " "

order author_year, first

replace by_group_funding = 2 if by_group_funding == 0

replace by_group_reference = 2 if by_group_reference == 0

*** ANALYSIS

putdocx begin

putdocx paragraph, style(Title) font(Arial, 20)

putdocx text ("Meta-Analyses: CT fracture detection with commercial AI products")

foreach var of varlist by_group_funding by_group_bias by_group_region by_group_commercial1 by_group_commercial2 by_group_level_external by_group_level_internal by_group_reader by_group_cohort by_group_reference by_group_dataset by_group_com_ex_in {

display as error("`var'")

local lbe: variable label `var'

putdocx paragraph, style(Heading1) font(Arial)

putdocx text ("By group: `lbe'"), linebreak

bygroup_forest, var(`var') studylabel(author_year) color(blue) model(random) save_dta(0) size(3.5) sav("$sav_name") legend_adapt(1)

local pdfname = "$sav_name" + upper(subinstr("`var'","by_group_","",1))

putdocx paragraph, font(Courier, 1)

putdocx image "output/`pdfname'.jpg", width(6)

putdocx pagebreak

putdocx table tbl4 = matrix(SENS), rownames colnames

putdocx table tbl4(.,.), font("Arial",8) nformat(%4.2f)

putdocx table tbl5 = matrix(SPEC), rownames colnames

putdocx table tbl5(.,.), font("Arial",8) nformat(%4.2f)

putdocx table tbl6 = matrix(H), rownames colnames

putdocx table tbl6(.,.), font("Arial",8) nformat(%4.2f)

putdocx pagebreak

putdocx paragraph, font(Arial)

putdocx image "output/`pdfname'_s.jpg", width(6)

putdocx text ("$studies")

}

local name = "SUMMARY"

putdocx save "$sav_name`name'", replace

## Helpfiles

capture prog drop bygroup_forest

**program bygroup_forest**

syntax, var(name) studylabel(name) [model(string) sav(string) color(string) save_dta(integer 0) size(real 2) note(string) title(string) extra_stuff(string) legend_adapt(integer 0)]

version 18.0

qui{

preserve

local pdfname = "`sav'" + upper(subinstr("`var'","by_group_","",1))

if `save_dta' == 1 local download download(datasets/dta_`var')

keep if !missing(`var')

decode `var', gen(`var'_string)

replace `var'_string = "{bf:"+ `var'_string + "}"

if "`model'" == "" local model random

local lbe: variable label `var'

if "`title'" == "" local title = "{bf: `lbe'}, size(*0.8)"

sort `var'_string author

gen study_number = _n

su study_number

local studies = ""

forvalues i=1/`r(max)'{

local study_no = study_number[`i']

local study_name = `studylabel'[`i']

local pre = "; "

if `i' == 1 local pre = ""

local studies = "`studies'" + "`pre'`study_no' = `study_name'"

global studies = `"`studies'"'

}

local legendo_new = ""

if `legend_adapt' == 1{

levelsof `var', local(level)

local label_text = ""

local counter = 1

foreach l of local level{

local lbe_value: label (`var') `l'

local label_text = `"`label_text'"' + " `counter' " + `""`lbe_value'""'

local ++counter

}

local legendo_new = `"legend(position(6) order(`label_text') col(2) size(*1.0))"'

}

local metadta = `"metadta tp fp fn tn, `extra_stuff' studyid(`studylabel') sortby(`var') model(`model') dp(2) `download' sumtable(all) by(`var'_string) stratify soptions(xtitle("False positive rate") bubbleid bubbles xlabel(0(0.2)1, format(%3.1f)) xscale(range(0 1)) ytitle("Sensitivity") `legendo_new' yscale(range(0 1)) ylabel(0(0.2)1,grid format(%3.1f)) graphregion(color(white)) plotregion(margin(medium)) xsize(15) ysize(15)) foptions(graphsave(metadta_`var') note(`note') title("") texts(`size') grid graphregion(color(white)) xlabel(0.5, 0.6, 0.7, 0.8, 0.9, 1) diamopt(color(`color')) pointopt(msymbol(s)msize(1)) olineopt(color(`color') lpattern(dash)))"'

order `studylabel' tp fp fn tn `var', first

noisily list `studylabel' tp fp fn tn `var'

noisily `metadta'

matrix define SPEC = e(absoutsp)

matrix define SENS = e(absoutse)

matrix define H = e(vcovar)

graph save "output/`var'_scatter.gph", replace

graph export "output/`pdfname'_s.pdf", replace as(pdf)

graph export "output/`pdfname'_s.jpg", replace width(3000) quality(100) as(jpg)

rm "output/`var'_scatter.gph"

graph use metadta_`var'.gph

graph play "helpfiles/format_axis0_5"

*** get rows of graph and resize

levelsof `var' , local(lev)

local lev_counter = 0

qui foreach l of local lev{

local ++lev_counter

su `var' if `var' == `l'

local numb = r(N)

if `numb'>1 local ++lev_counter

local lev_counter = `lev_counter' + `numb'

local ++lev_counter

}

local no_studies = `lev_counter'

local ysize = (3.865 + 7.52 / 17 * (`no_studies' +1)) /2.53

resize , ysize(`ysize') xsize(7.5)

local ysize_exp = round(`ysize'*500)

graph export "output/`pdfname'.pdf", replace as(pdf)

graph export "output/`pdfname'.jpg", replace width(3750) height(`ysize_exp') quality(100) as(jpg)

rm "metadta_`var'.gph"

restore

}

end

cap prog drop import_dataset

**program import_dataset**

syntax , filename(string) [type_on(integer 1)]

version 16.0

qui import excel "`filename'", sheet("codebook") firstrow clear

local dumdum = "Variablename Variablelabel Labelname Labeldefinition Variabletype"

foreach vor of local dumdum {

qui capture confirm variable `vor'

if _rc {

di in red "`vor' does not exist or is written wrong!"

qui local any_error = 1

}

else {

qui tostring `vor', replace

qui replace `vor' = "" if `vor' == "."

}

}

display as txt "START IMPORTING EXCEL FILE:"

display as txt ""

qui local any_error = 0

qui gen length_var = length(Variablename)

qui su length_var

if `r(max)' > 32 {

display("")

display in red "Error: Variable(s) too long!"

tab Variablename if length_var > 32

qui local any_error = 1

}

tempvar spec_char any_spec_char

qui egen `spec_char' = sieve(Variablename), char(0123456789abcdefghijklmnopqrstuvwxyz_ABCDEFGHIJKLMNOPQRSTUVWXYZäöüÄÖÜ)

qui gen `any_spec_char' = length_var - length(`spec_char')

qui drop length_var

qui su `any_spec_char'

if `r(max)' > 0 {

display("")

display in red "Error: Variable(s) containing invalid characters (spaces, /, -,. , ... etc.)!"

tab Variablename if `any_spec_char' > 0

qui local any_error = 1

}

qui if `any_error' == 1 {

exit

}

qui local total_vars = _N

qui gen id_dummy123 = _n

// qui drop if missing(Variablelabel)

qui save codebook_file, replace

qui import excel "`filename'", sheet("data") firstrow clear

local how_many = _N

unab varlist2 : _all

qui gen id_dummy123 = _n

qui merge 1:1 id_dummy123 using codebook_file

qui drop _merge

local error_flag = 0

local miss_list = ""

qui foreach var of varlist `varlist2' {

// display("`var'")

qui gen dumy_123 = strpos(Variablename,"`var'")

qui su dumy_123

if `r(max)' == 0 {

local miss_list = "`miss_list'" + " `var'"

}

qui drop dumy_123

}

if length("`miss_list'") > 1 {

local error_flag = 1

di in red "The following variables are not found in codebook and are labeled with the variable name without labeling values:"

di in red "`miss_list'."

}

local miss_list = ""

qui forval i = 1/`total_vars' {

local var_name = Variablename[`i']

if strpos("`varlist2'","`var_name'") == 0 {

local miss_list = "`miss_list'" + " `var_name'"

}

}

if length("`miss_list'") > 1 {

local error_flag = 1

di in red "The following variables in the codebook sheet are NOT found in the data sheet and are ignored:"

di in red "`miss_list'."

}

qui rename (Remark Variablename Variablelabel Labelname Labeldefinition Variabletype) (=_123)

forval i = 1/`total_vars' {

local any_error = 0

local var_name = Variablename[`i']

local var_label = Variablelabel[`i']

local var_labelname = Labelname[`i']

local var_labeldef = Labeldefinition[`i']

local var_type = Variabletype_123[`i']

*** Assign variable label

display as txt "Labeling: `var_name'"

capture label var `var_name' "`var_label'"

qui if _rc {

di in red "Error in labeling `var_name' with '`var_label''. Is Variable present in Codebook AND Data sheet?"

local any_error = 1

local error_flag = 1

}

*** Assign variable type if option is on

if `type_on' != 0 {

if strpos("`var_type'", "binary") !=0 local var_type byte

if strpos("`var_type'", "integer") | strpos("`var_type'", "int") !=0 local var_type int

if strpos("`var_type'", "float") !=0 local var_type double

if strpos("`var_type'", "string") ==0 capture recast "`var_type'" `var_name', force

if strpos("`var_type'", "date") >0 {

capture format `var_name' %td

if _rc {

di in red "Error in type of variable (`var_type') for variable `var_name'."

local any_error = 1

local error_flag = 1

}

if strpos("`var_type'", "datetime") >0 capture format `var_name' %tc

}

else if _rc {

di in red "Error in type of variable (`var_type') for variable `var_name'."

local any_error = 1

local error_flag = 1

}

}

*** Assign variable value label

if "`var_label'" == "" {

capture label var `var_name' "`var_name''"

if _rc {

di in red "Error in labeling `var_name'. Is Variable present in Codebook and Data sheet?"

local error_flag = 1

}

}

if `any_error' == 0 {

if Labelname[`i'] == "none" | Labelname[`i'] =="" | Labelname[`i'] ==" " {

display as result "Check."

}

else {

if `"`var_labeldef'"' == "" {

di in red "Error in defining the label `var_labelname' for variable `var_name' as definition is missing! All labels have to be defined - otherwise remove label name."

local error_flag = 1

}

else

{

capture label define `var_labelname' `var_labeldef', replace

if _rc {

di in red "Error in defining the label `var_labelname' with the definition: " `"`var_labeldef'"' " - for `var_name'!"

local error_flag = 1

}

else {

capture label values `var_name' `var_labelname'

if _rc {

di in red "Error in labling `var_name' with `var_labelname'!"

local error_flag = 1

}

else {

display as result "Check."

} // rc 2

} // rc 1

} // label none else

}

}

} // any_error

if `error_flag' == 1 {

di in red " "

di in red "Any ERROR/s occured! Details see above."

}

else {

display as result " "

display as result "IMPORT SUCCESSFUL."

}

drop id_dummy123 Remark_123 Variablename_123 Variablelabel_123 Labelname_123 Labeldefinition_123 Variabletype_123

erase codebook_file.dta

qui drop if _n > `how_many'

end

## Diagnostic performance metrics

input str40 author_year int(N tp fp tn fn)

end

sort author_year

gen prev = (tp+fn)/N

gen sens = tp/(tp+fn) // recall

gen spec = tn/(tn+fp)

gen ppv = tp/(tp+fp) // precision

gen npv = tn/(tn+fn)

gen f1 = 2*tp/(2*tp+fp+fn)

foreach var of varlist prev sens spec ppv npv{

gen `var'_l = .

gen `var'_u = .

}

local tot = _N

forval c=1/`tot'{

// prev 95% CI

cii proportions (N[`c']) (tp[`c']+fn[`c']), wilson

replace prev_l = r(lb) in `c'

replace prev_u = r(ub) in `c'

order prev prev_l prev_u, last

// sens 95% CI

cii proportions (tp[`c']+fn[`c']) (tp[`c']) , wilson

replace sens_l = r(lb) in `c'

replace sens_u = r(ub) in `c'

order sens sens_l sens_u, last

// spec 95% CI

cii proportions (tn[`c']+fp[`c']) (tn[`c']) , wilson

replace spec_l = r(lb) in `c'

replace spec_u = r(ub) in `c'

order spec spec_l spec_u, last

format sens spec ppv npv %4.2f

// ppv 95% CI

cii proportions (tp[`c']+fp[`c']) (tp[`c']), wilson

replace ppv_l = r(lb) in `c'

replace ppv_u = r(ub) in `c'

order ppv ppv_l ppv_u, last

// npv 95% CI

cii proportions (tn[`c']+fn[`c']) (tn[`c']), wilson

replace npv_l = r(lb) in `c'

replace npv_u = r(ub) in `c'

order npv npv_l npv_u, last

local ++c

}

foreach var of varlist prev* sens* spec* ppv* npv* {

replace `var' = `var' *100

}

format prev* sens* spec* ppv* npv* %4.1f

clonevar prev_perc = prev

label var prev_perc "Incidence (%)"

label var ppv "Positive predictive value (%)"

label var npv "Negative predictive value (%)"

label var sens "Sensitivity (%)"

label var spec "Specificity (%)"

br

## Graphical abstract

cap prog drop graph_scatter

program def graph_scatter

version 18.0

syntax , var_y(name) var_x(name) name_var(name) [ weight(name) colorpalette(string) color_points(string) legendi(integer 0) legendi_size(real 0.5) legendi_col(integer 8) *]

*** Stuff

local palette30 = `" "31 119 180" "255 127 14" "44 160 44" "214 39 40" "148 103 189" "140 86 75" "227 119 194" "127 127 127" "188 189 34" "23 190 207" "174 199 232" "255 187 120" "152 223 138" "255 152 150" "197 176 213" "196 156 148" "247 182 210" "199 199 199" "219 219 141" "158 218 229" "0 109 44" "166 54 3" "99 99 99" "49 130 189" "117 107 177" "107 174 214" "82 84 163" "189 189 189" "140 140 140" "0 0 0" "'

*** Settings

local total_obs = _N

local twoway_text = ""

local legend_text = ""

qui gen _dummy123 = _n

if "`colorpalette'" == "" local colorpalette = `"`palette30'"'

qui forval i = 1/`total_obs'{

local name = `name_var'[`i']

colorpalette `colorpalette', nograph

if "`color_points'" == ""{

local col = `"color("`r(p`i')'") mlabcolor("`r(p`i')'")"'

local colori = `""`r(p`i')'""'

}

else {

local col = "mlabcolor(`color_points') color(`color_points')"

local colori = `"`color_points'"'

}

local legend_text = `"`legend_text'"' + "`i' " + `""`i': `name'""'

local r = weight[`i']

local y_val = `var_y'[`i']

local x_val = `var_x'[`i']

local lr = `x_val' - `r'

local ur = `x_val' + `r'

local circle = `"(function y = `y_val'+sqrt(`r'*`r' - (x-`x_val')^2), range(`lr' `ur') color(`colori')) (function y = `y_val'-sqrt(`r'*`r' - (x-`x_val')^2), color(`colori') range(`lr' `ur') )"'

local twoway_text = `"`twoway_text'"' + `"(scatter `var_y' `var_x' if _n== `i', `col' mlabel(_dummy123)) "'

}

local lbe_y: variable label `var_y'

local lbe_x: variable label `var_x'

if `legendi' == 0 local legend_text="legend(off)"

if `legendi' == 1 local legend_text=`"legend(order(`legend_text') position(6) col(`legendi_col') size(*`legendi_size'))"'

*** Graph

twoway `twoway_text' , ytitle("`lbe_y'") xtitle("`lbe_x'") `legend_text' `options'

qui drop _dummy123

end

input str40 Author int(N TP FP TN FN) ///

float(Prev Prev_LCI Prev_UCI ///

Sens Sens_LCI Sens_UCI ///

Spec Spec_LCI Spec_UCI ///

PPV PPV_LCI PPV_UCI ///

NPV NPV_LCI NPV_UCI ///

F1)

end

rename _all, lower

sort author

gen no = _n

order no, first

local lbe: variable label ppv

replace author = "v.d. Wittenboer (2024)" in 25

replace author = subinstr(author," et al.","",1)

//gen weight = 1

//graph_scatter, name_var(author) var_y(prev) var_x(ppv) ylabel("0(20)100") xlabel("0(20)100") legendi(1) name(ppv_leg, replace) legendi_size(0.9) legendi_col(6)

//drop weight

label var prev "{bf:Fracture Incidence [%]}"

label var sens "{bf:Sensitivity [%]}"

label var spec "{bf:Specificity [%]}"

label var npv "{bf:Negative Predictive Value [%]}"

label var ppv "{bf:Positive Predictive Value [%]}"

foreach var of varlist sens spec npv ppv{

local lbe: variable label `var'

gen weight = `var'_uci - `var'_lci

graph_scatter, name_var(author) var_y(prev) weight(weight) var_x(`var') xtitle("") ylabel("0(20)100") xlabel("0(20)100") legendi(0) name(`var', replace) title("`lbe'", size(*0.8))

drop weight

}

graph combine sens spec npv ppv, col(2) name(combined, replace) iscale(*1.2)
